# Mechanical Modeling and Experimental Validation of a Front-Push Orthopedic Brace: Compressive–Shear Force Characterization Under Controlled Misalignment

**DOI:** 10.3390/bioengineering13050491

**Published:** 2026-04-23

**Authors:** Mirko Zisi, Vincenzo Ricci, Alessandro Rocchi, Vincenzo Canali

**Affiliations:** 1 Habitus et Motus, Via Nazionale 144, 40046 Alto Reno Terme, Italy; 2 VR Compositi di Ricci Vincenzo, Via Casale 20, 43035 Felino, Italy; info@vrcompositi.it; 3 Postura e Sport Srl, Via Alberto Savinio 26F, 43123 Parma, Italy; alessandro.rocchi71@gmail.com (A.R.); info@posturaesport.com (V.C.)

**Keywords:** scoliosis brace, thoraco-lumbo-sacral orthosis, biomechanical modeling, orthopedic device validation, spinal biomechanics, corrective torque, mechanotransduction

## Abstract

Scoliosis is a three-dimensional spinal deformity that may affect musculoskeletal alignment, respiratory mechanics, and neuromotor control. Rigid thoraco-lumbo-sacral orthoses (TLSOs) remain the primary conservative treatment during skeletal growth. Most brace systems rely on three-point pressure mechanisms that primarily generate lateral compression forces, while the contribution of shear components to corrective biomechanics has been insufficiently quantified. This study presents the experimental and analytical validation of the Canali Front-Push Orthopedic Brace, a rigid orthotic system designed to generate controlled compressive and shear forces through a frontal thrust mechanism and anterior rib cage engagement. By applying anterior force, the device reduces the frontal-plane lever arm, thereby limiting the mechanical moment that contributes to transverse plane rotation. An instrumented four-segment torso model derived from the internal CAD geometry of the brace was developed to independently measure upper compression, lower compression, and intersegmental shear forces. Controlled misalignment conditions (0 mm, 2 mm, and 4 mm) were introduced to simulate asymmetric engagement of the orthosis. Three load cell configurations (200 N and 500 N capacity) were tested. Mechanical endurance of the rack–latch fastening system was also evaluated. A predictive shear–misalignment relationship was derived and experimentally validated. Peak compressive forces reached approximately 370 N, while shear forces increased from less than 40 N under symmetric alignment (D0) to approximately 170 N under maximal misalignment (D4). Shear activation demonstrated near-linear proportionality to imposed geometric asymmetry (R^2^ > 0.94). Following cyclic loading, the fastening system stabilized mechanically around 300 N. Measurement repeatability showed a coefficient of variation below 5%. These findings demonstrate that the brace produces predictable and controllable shear activation while maintaining high mechanical repeatability. The results provide a quantitative biomechanical framework for understanding shear-induced corrective mechanics in scoliosis bracing and support future studies integrating computational modeling and clinical validation. The proposed mechanical framework may contribute to the development of next-generation orthotic strategies aimed at controlling spinal rotation through vector modulation rather than purely compressive correction.

## 1. Introduction

Scoliosis is a 3D deformity of the spine that severely affects the quality of daily living. In severe cases, scoliosis affects the musculoskeletal, respiratory, and nervous systems. Over 600,000 people suffering from scoliosis are being treated every year [1]. Although the exact cause is often unknown, scoliosis is generally classified depending on its etiology: idiopathic, congenital, or neuromuscular [2]. Idiopathic scoliosis can further be subdivided according to the age of onset as infantile (age 0–3), juvenile (age 4–9), or adolescent (age 10 up to skeletal maturity) [3,4]. Congenital scoliosis is a result of embryological malformation; thus, children are typically diagnosed at a very early age. Neuromuscular scoliosis is associated with secondary factors such as spinal cord trauma, cerebral palsy, spina bifida, or muscular dystrophy and can occur later in life [5]. Among these three groups, idiopathic scoliosis tends to be the most prevalent worldwide with approximately 2–4% of children between 10 and 16 years of age being diagnosed.

Initially, scoliosis is screened for via physical examination but only fully diagnosed by either CT scan, MRI, or X-ray. Based on the degree angle, the severity of scoliosis is determined. Curves of 10° or less are considered mild; between 10 and 50°, it is considered moderate, while above 50° is severe [6]. Curves under 20° usually only require monitoring and thus no therapeutic intervention. Curves between 20 and 40° tend to require some form of bracing. Severe scoliosis often requires surgery, typically spinal fusion. Some risk factors for developing scoliosis include gender, age, ethnicity, and family history.

The general goal of bracing is to maintain the curve below 50° upon patient maturation. Although effective, bracing tends to prevent curves from worsening rather than permanently correcting or improving [6]. The rate of surgery after bracing is between 11 and 42.5%, depending on the previous treatment methods employed. If treatment was rather conservative, there is a greater chance of surgery [7]. Surgical options are considered when a curve exceeds 45° in immature patients and 50° in mature. The goal of surgery is to halt the progression and improve spinal curvature and balance.

The use of bracing was reduced in the early 20th century due to innovations in surgical treatment [8]. However, it again attracted attention when complications started to appear in surgical treatments in the late 20th century. Several rigid braces have been developed in the past with the aim of either correcting the Cobb angle or halting its progression. Milwaukee [9,10,11,12], Boston [13,14,15], Lyon [16,17], and Chêneau [18,19,20] braces are some of the often-applied rigid braces to treat scoliosis. These braces have different correction principles, and they are developed to treat different scoliosis curves. Braces require patients to wear them for 18 to 23 h per day to be most effective [21]. To increase patient compliance, nighttime bracing, such as Charleston brace [22] and Providence brace [23], was introduced. These part-time braces had an aggressive correction effect and were mostly used for single curve thoracolumbar scoliosis. A few soft braces, such as SpineCor [24], SpinealiteTM [25,26], and TriaC [27], have also been developed in the past to enhance comfort and halt the Cobb angle progression.

Although rigid braces are considered to be more effective in the treatment of scoliosis [28]. There are certain shortcomings associated with the rigid braces: (i) the rigid static nature of these braces largely limit mobility of the spine and can result in muscle atrophy, spine stiffness, and flat back issues; (ii) rigid braces affect cardiopulmonary efficiency; (iii) reduction in the physiotherapy exercise potential; (iv) long construction time; (v) socio- economic implications; (vi) rigid braces causes abnormal bone deformation and skin breakdown [29]. Soft braces, on the other hand, are more compliant and enhance comfort but have a less corrective effect. Soft braces can be used to halt the progression of the Cobb angle and, in some cases, correct it if the severity of the curve is not too excessive, in specific growth phases [30]. Several clinical studies [31,32] have been carried out to compare the outcome of the soft and rigid braces. There is not enough evidence to deduce an explicit conclusion on the effectiveness of the interventions.

Traditional TLSO braces focus primarily on lateral compression [33,34]. However, shear forces contribute to vertebral rotational correction [35,36] and anterior column engagement may influence sagittal mechanics. Refs. [37,38] and mechanical asymmetry can modulate corrective vectors.

Recent studies have investigated simplified mechanical and computational modeling approaches to improve the understanding of orthotic system behavior and force transmission mechanisms, emphasizing the importance of integrating experimental measurements with analytical modeling frameworks [39]. The combination of experimental data and computational modeling allows a more comprehensive interpretation of how corrective forces are generated, transmitted, and distributed within brace–torso systems.

In parallel, recent advances in biomechanical modeling have increasingly focused on finite element methods to simulate brace–torso interaction and vertebral response under corrective loading conditions. These models highlight the role of both compressive and shear force components in influencing spinal deformation, load distribution, and rotational behavior. Nevertheless, despite the growing use of computational models, experimental validation of shear force generation in orthotic systems remains limited, particularly under controlled mechanical conditions. This gap highlights the need for experimental studies capable of quantifying shear and compression components and relating them to simplified biomechanical models.

The Canali Front-Push Brace introduces controlled frontal thrust capable of inducing both compressive and shear components.

The aim of this study was to experimentally quantify compressive and shear forces generated by the brace, develop a predictive mechanical model of shear activation, evaluate the repeatability and mechanical stabilization of the tightening system, and assess the proportional relationship between applied force and geometric misalignment.

## 2. Materials and Methods

The experimental methodology consisted of three main phases: (1) material characterization of the brace components, (2) force measurements using an instrumented torso model under controlled misalignment conditions, and (3) development of an analytical biomechanical model to estimate induced rotational moments. Additional methodological details and extended datasets are provided in the Supplementary Materials.

The mechanical characterization of the neoprene interface layer, the lacing system, and the rack–latch tightening mechanism highlights a hierarchical stiffness distribution within the orthotic structure. The neoprene layer exhibited a very low Young’s modulus (E ≈ 0.401 MPa), confirming its role as a compliant interface capable of distributing loads and absorbing part of the mechanical energy transmitted by the brace.

In contrast, the lacing system showed a significantly higher stiffness (E ≈ 220–232 MPa, K ≈ 55–58 N/mm), indicating that the laces act as the primary tensile elements responsible for transmitting the tightening forces across the orthotic structure.

The rack–latch mechanism demonstrated an intermediate mechanical behavior, with stress–strain curves indicating an effective stiffness in the range of 95–105 MPa. Repeated loading cycles revealed an initial peak force of approximately 373 N, followed by progressive stabilization around 300 N, suggesting mechanical settling of the system components and defining a reliable operational load threshold for the tightening mechanism.

Together, these results reveal a functional stiffness hierarchy within the orthotic device. The tightening force is primarily generated and regulated by the rack–latch mechanism, transmitted through the high-stiffness laces, and finally distributed to the thoracic interface through the compliant neoprene layer. This mechanical architecture ensures efficient force transmission while maintaining controlled load distribution at the body–brace interface, which is essential for both corrective effectiveness and patient comfort.

### 2.1. Testing Device

Mechanical testing of brace materials and components was performed using a MaCh5 Smart Universal Testing Machine (MaCh3D, Parma, Italy). The MaCh5 is a compact electromechanical testing platform capable of displacement-controlled, load-controlled, and combined loading modes, and was selected for its compatibility with custom fixtures and for its load and displacement resolution, which are appropriate for the force ranges investigated in the present study. The main technical specifications of the device are summarized in Table X. The machine has a maximum load capacity of 5 kN, a load measurement accuracy of ±0.05 N, a displacement measurement accuracy of ±0.24 × 10^−3^ mm, and a stroke range of 100 mm. The crosshead speed is continuously adjustable between 0.1 and 30 mm/min, and data acquisition was performed at the sampling rates specified for each test. Prior to each test session, the zero-offset of both the load cell and the displacement encoder was verified and reset according to the manufacturer’s procedure.

### 2.2. Neoprene

**Figure 1:**
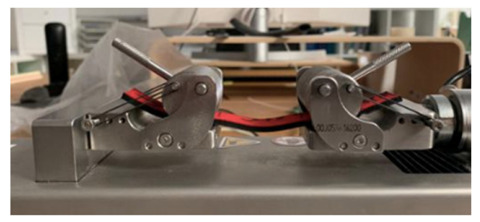
Neoprene.

Neoprene (Figure 1) was used as the inner interface layer of the torso model, onto which the rigid structural components of the orthotic system were mounted. This compliant layer was selected to simulate the soft contact interface between the brace and the thoracic surface.

Tensile testing of the neoprene layer was performed to estimate an equivalent elastic modulus under application-relevant conditions. Rectangular specimens were cut from the neoprene sheet after removal of the composite shells, while residual adhesive and the cotton surface layer were intentionally retained. This choice was made because the aim of the test was not to determine the nominal material properties of neoprene alone, but rather to evaluate the effective mechanical response of the interface layer as used in the orthotic system. Specimens measured 145 × 20 mm with a thickness of 3 mm. Tests were performed at a constant crosshead speed of 20 mm/min with a sampling rate of 10 Hz. The nominal gauge length was 45 mm, and tests were stopped at a total crosshead displacement of 50 mm. Roller-type grips were used. As the eccentric roller clamping mechanism induced a compressive pre-load during mounting, a slightly increased effective gauge length and an initially curved specimen configuration were observed. This effect was corrected during post-processing by applying foot-offset correction and gauge length adjustment. A total of five specimens were tested. During clamping, a slight reduction in pre-tension was observed, resulting in minor outward curvature of the specimen. This effect is attributable to the mechanical characteristics of the gripping system and the high compliance of the material. The phenomenon was considered during post-processing and did not affect the reliability of the measurements. Tensile tests were performed at a constant crosshead speed of 20 mm/min. The measured Young’s modulus of the neoprene samples was

E = 0.401 ± 0.044 MPa

This value confirms the high compliance and energy absorption capacity of the material, which is consistent with its role as a cushioning and load-distributing interface within the orthotic system.

**Figure 2:**
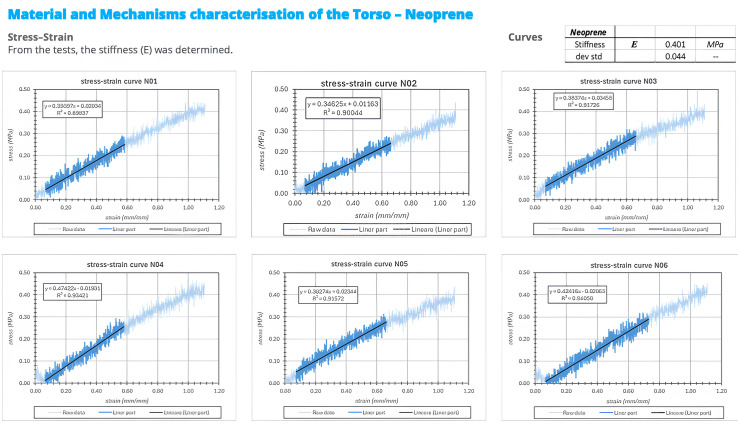
Force–displacement curves.

**Figure 3:**
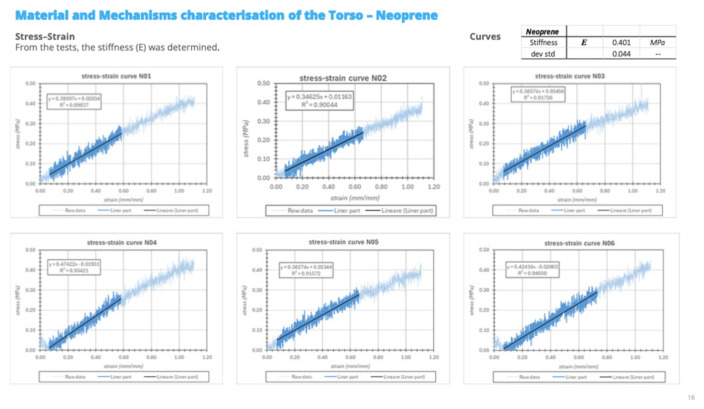
The corresponding stress–strain curves. The symbols -- mean “standard deviation without explicit unit”.

Force–displacement curves (Figure 2) showed a consistent and quasi-linear response across all tested samples, indicating stable mechanical behavior and good repeatability of the material properties. The corresponding stress–strain curves (Figure 3) confirmed the high compliance of the neoprene layer, with a measured Young’s modulus of 0.401 ± 0.044 MPa, demonstrating the capacity of the material to act as a compliant interface capable of distributing loads and absorbing part of the mechanical energy transmitted by the orthotic structure.

### 2.3. Laces

**Figure 4:**
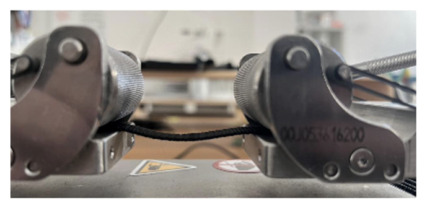
Laces.

Laces (Figure 4) were used to connect the rigid structural components of the torso to the neoprene interface layer. Their primary function is to maintain the structural integrity of the orthotic assembly and to transmit tensile forces that stabilize the rigid elements while providing lateral support to the neoprene interface.

Two rectangular specimens with dimensions of 92 × 10 × 1 mm were prepared for tensile testing. The effective gauge length between the grips was 40 mm.

As observed with the neoprene specimens, a slight loss of initial tension occurred during clamping, leading to minor outward curvature of the sample. This behavior is attributable to the flexibility of the material and the mechanical characteristics of the gripping system. The effect was considered during data processing and did not compromise the validity of the measurements.

Tensile tests were conducted to evaluate the elastic response of the lacing system. The measured Young’s modulus ranged between

E = 220–232 MPa

The corresponding spring constant was estimated as

K = 55–58 N/mm

These values indicate relatively high stiffness compared to the neoprene interface layer, allowing the laces to efficiently transmit tensile forces generated by the tightening mechanism.

The nylon straps were tested directly, without additional specimen preparation, because of their reduced dimensions (92 × 10 mm, thickness 1 mm). Only two test pieces were available. Tests were conducted at a constant crosshead speed of 5 mm/min and a sampling rate of 10 Hz, with a nominal gauge length of 40 mm. Roller-type grips were used. As observed for the neoprene specimens, a minor compressive effect occurred during clamping and was corrected during post-processing using the same foot-offset procedure. Load and crosshead displacement were recorded throughout testing.

**Figure 5:**
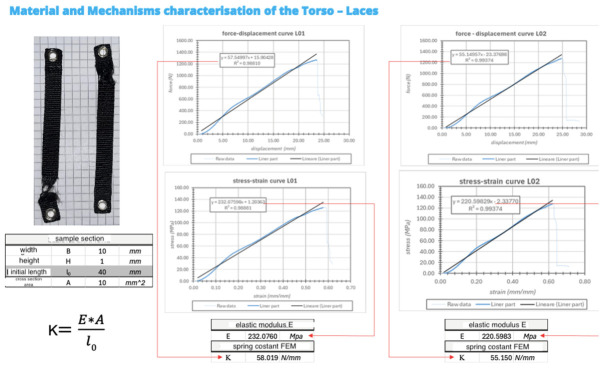
Tensile test. The blue lines indicate the points measured during the test, including noise, oscillations, and imperfections, and represent the material’s behavior. The solid black line is a mathematical simplification, approximating the most linear part of the behavior, while the dashed line indicates the portion of the graph chosen for the calculation. The symbol * represents multiplication.

Tensile tests (Figure 5) performed on the lacing system showed a linear force–displacement response, indicating elastic behavior within the tested range. The corresponding stress–strain curves yielded a Young’s modulus between 220 and 235 MPa, confirming the relatively high stiffness of the laces compared to the neoprene interface layer. The estimated spring constant ranged between 55 and 58 N/mm, demonstrating that the lacing system efficiently transmits tensile forces generated by the tightening mechanism while maintaining predictable elastic behavior.

### 2.4. Rack–Latch System

**Figure 6:**
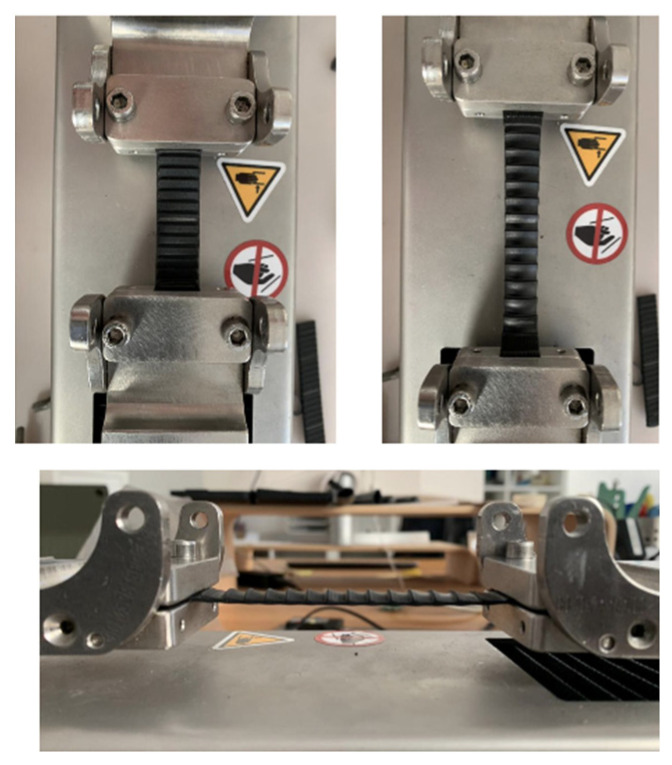
Rack–latch system.

Racks (Figure 6) were used in combination with their corresponding latch mechanisms to secure and clamp the torso structure. This system represents the primary tightening mechanism responsible for generating and maintaining the compressive forces within the orthotic assembly.

Two different test types were performed on the ratchet system: (i) stiffness characterization of the ratchet strap and (ii) measurement of the maximum static load sustained by the ratchet buckle before disengagement.

For ratchet strap stiffness, specimens were cut directly from an available strap. The strap had a width of 14.5 mm and an average thickness of 2.4 mm, corresponding to an average cross-sectional area of 34.8 mm^2^. Two specimens of length 80 mm were prepared. Clamp-type grips were used instead of roller grips because the higher forces involved and the saw-tooth profile of the strap made roller grips unsuitable. Tests were conducted at 5 mm/min with a sampling rate of 10 Hz and a nominal gauge length of 40 mm. A slight initial bowing effect was observed and corrected during post-processing.

For buckle maximum load testing, the ratchet strap was clamped at its trailing end to the moving crosshead, while the buckle was mounted on a custom PLA 3D-printed support fixed to the stationary frame. After zeroing the load cell, slight pre-tension was applied to ensure proper tooth engagement. A monotonic displacement-controlled test was then performed until tooth escape and re-engagement occurred, which was taken as the end condition. Five repeated loading cycles were performed on the same buckle–strap assembly engaged on the same tooth row. Because of the geometric misalignment between the loading direction and the strap axis, the effective elongation of the system was calculated geometrically from the measured crosshead displacement.

During clamping, a slight loss of initial tension was observed, resulting in minor outward curvature of the specimen. This behavior is associated with the interaction between the rack structure and the gripping system and was accounted for during data processing.

Repeated tensile loading cycles were performed to evaluate the mechanical stability of the rack–latch mechanism. A progressive reduction in the maximum force was observed during the first loading cycles, followed by stabilization of the system:

• Fmax_1_ = 373 N
• Fmax_2_ = 343 N
• Fmax_3–5_ ≈ 300 N (stabilized value)

This behavior is indicative of an initial mechanical settling of the system components, after which the mechanism reaches a stable load-bearing condition. The stabilized force level of approximately 300 N represents a realistic operational threshold for the tightening mechanism and provides an upper safety limit for brace loading during clinical use.

**Figure 7:**
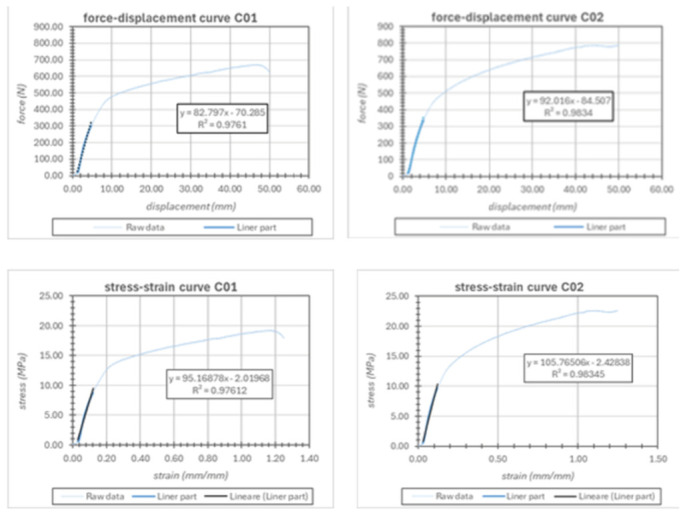
The force–displacement curves of the rack–latch mechanism.

The force–displacement curves of the rack–latch mechanism (Figure 7) showed a rapid initial force increase followed by a quasi-linear elastic region. The corresponding stress–strain curves indicated an effective stiffness ranging between approximately 95 and 105 MPa. Repeated loading cycles revealed an initial peak force of 373 N, followed by progressive stabilization around 300 N, suggesting the mechanical settling of the system and defining a reliable operational load threshold for the tightening mechanism.

### 2.5. System-Level Mechanical Interpretation

A dedicated test bench was designed to measure the corrective forces exerted by the brace on the trunk. The apparatus was conceived to resolve the total load into three components: upper compressive force, lower compressive force, and transverse shear force associated with asymmetric loading conditions. The biomechanical rationale is that, under symmetric loading, the upper and lower brace reactions are balanced, whereas under asymmetric conditions the difference in reaction forces generates a residual transverse load that, in vivo, would be equilibrated by the spine. The test bench was specifically designed to capture this load distribution. The torso was approximated by four rigid blocks representing trunk quadrants, each free to move independently in the transverse direction. Load cells were mounted between adjacent blocks to resolve the corrective forces, including a specific element dedicated to the measurement of inter-level shear. The rigid blocks were derived from the internal CAD geometry of the brace and mounted on linear guides to allow controlled relative motion.

Three load cell configurations were used during the test campaign. The initial setup (CTC20) employed two compression load cells and one cantilever shear load cell, all with a 200 N full-scale range. Because the force range exceeded this capacity, the setup was progressively redesigned. An intermediate CCC20 configuration, based on three 200 N compression load cells, was used to validate the modified architecture. The final CCC50 configuration, consisting of three 500 N compression load cells, was adopted for the main brace-force characterization. Generic nomenclature was used for the load cells: LC1 and LC2 for the lower and upper compressive channels, respectively, and LCT for the transverse shear-related channel.

## 3. Experimental Results

Brace tests were performed in three main steps: brace mounting, pre-tensioning, and progressive loading. During mounting, the brace was positioned on the test rig with the rear ratchet fixed at a constant initial width and the frontal ratchets inserted but not yet fully tightened. At the end of mounting, all load cell channels were zeroed. During pre-tensioning, the frontal buckles were actuated in a predefined sequence until the target channel showed a minimal non-zero reading, while maintaining all load cell readings below 10 N. The target channel depended on the imposed misalignment condition. Progressive loading was then carried out using the reverse buckle sequence in order to promote load balance. After each actuation, a short dwell time was allowed for stabilization before recording the load cell values. In the CCC50 configuration, the tightening procedure was modified to alternate front and rear loading sequences in order to avoid torsional deformation of the rig at high loads. A preliminary protocol was also used to quantify the contribution of pressure pads by interrupting the loading sequence at approximately 40 N and subsequently activating the pads in controlled screw increments.

The results are organized into three main components: (i) experimental characterization of compressive and shear forces under controlled misalignment conditions, (ii) validation of the predictive shear–misalignment mechanical model, and (iii) biomechanical interpretation of the induced rotational moments generated by the orthotic system.

### 3.1. Nominal Alignment (D0)

Under nominal alignment (D0), the force profile exhibited an oscillat pattern during sequential tightening of the brace mechanism. This behavior reflects the progressive engagement of the tightening system across successive latch positions. Under these symmetric conditions, shear forces remained minimal throughout the tightening process.

Maximum compressive forces were recorded using the CCC50 load cell configuration, reaching approximately 350 N in the upper compartment and 300 N in the lower compartment, while the measured shear force remained below 40 N. These findings indicate that under geometrically symmetric conditions the system primarily generates compressive loading, confirming that shear forces remain negligible in the absence of imposed misalignment.

### 3.2. 2 mm Misalignment (D2)

When a 2 mm geometric misalignment (D2) was introduced between the torso segments, a substantial increase in shear force was observed. Under these conditions, the shear component became comparable to the compressive forces generated by the brace. The tightening process also revealed a progressive redistribution of forces between the upper and lower compressive compartments.

Peak shear forces reached approximately 120 N when measured using the 20 kg load cell configuration, indicating that even moderate geometric asymmetry can significantly activate shear force generation within the orthotic system.

### 3.3. 4 mm Misalignment (D4)

Under maximal misalignment (D4 = 4 mm), shear forces became the dominant component during the early stages of the tightening process. During the initial tightening clicks, the compressive load cells recorded near-zero values, while the shear load cell immediately detected forces exceeding 40–60 N.

As tightening progressed toward the final latch positions, compressive forces gradually increased; however, the shear component remained substantial. At full tightening, peak shear forces exceeded 150–170 N. These results demonstrate that the brace is capable of generating controlled anterior corrective shear forces in the presence of geometric asymmetry.

The results demonstrate that shear forces are negligible under symmetric alignment but increase proportionally with geometric misalignment. At 4 mm asymmetry, shear forces reached up to 170 N, indicating that the brace can generate controlled anterior shear components capable of contributing to rotational correction. At maximal misalignment (D4), the shear component reached approximately 170 N, corresponding to nearly 50% of the compressive force. This resulted in a resultant force vector deviation by approximately 28° from pure compression. Such vector modulation allows the orthosis to generate rotational moments within the biomechanical range reported in spine modeling studies (≈5–20 Nm), suggesting potential relevance for axial rotational control. The brace was tested under four strap configurations: no strap (NS), single strap (SS), double strap (DS), and double bonded strap (DBS). Three displacement conditions were also investigated: D0, representing the symmetric condition without imposed asymmetry; D2, obtained by inserting two 2 mm spacers; and D4, obtained by inserting two 4 mm spacers. In mechanical terms, D2 and D4 represent controlled geometric asymmetries of the test rig used to simulate asymmetric brace–torso engagement. A total of 19 test conditions were investigated, combining load cell configuration, strap configuration, and imposed displacement.

### 3.4. Instrumented Torso Model

**Figure 8:**
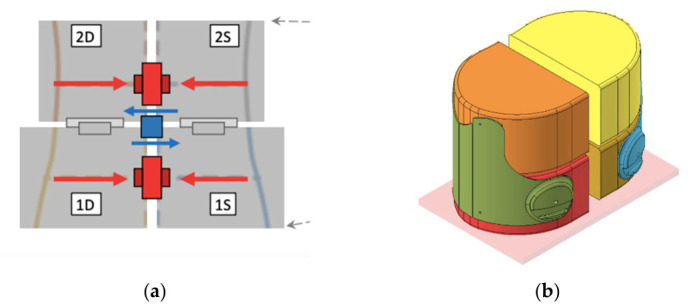
(**a**) Instrumented torso model used to measure upper compressive force, lower compressive force and intersegmental shear force generated by the brace; (**b**) CAD-derived torso geometry segmented into four independent compartments for force measurement. The red arrows are compressive forces, the blue arrows are shear forces and the dashed center line interfaces the segments where the shear is generated.

A geometrically congruent torso model (Figure 8) was derived from the internal CAD geometry of the brace and segmented into four independent compartments, as illustrated in Figure 8a,b. This configuration allowed the independent measurement of the main mechanical variables generated by the orthotic system during tightening.

The measured variables included the upper compressive force (Fc_upper), the lower compressive force (Fc_lower), and the intersegmental shear force (F_shear). To investigate the influence of geometric asymmetry on force generation, three controlled misalignment conditions were introduced between the torso segments: D0 (0 mm) representing nominal alignment, D2 (2 mm) representing moderate asymmetry, and D4 (4 mm) representing maximal misalignment within the tested range.

Each experimental condition was tested in three repeated trials to ensure measurement repeatability and to allow subsequent statistical analysis of the recorded forces.

The imposed misalignment values (2–4 mm) represent controlled geometric asymmetries within the orthotic system and were selected to simulate realistic variations in brace fitting and adjustment. These may arise from differential tightening, component positioning, or intentional modulation of corrective forces. Therefore, misalignment should be interpreted as a mechanical control parameter rather than a direct clinical measurement.

### 3.5. Mechanical Modeling of Shear Activation

To interpret the experimental findings, a simplified mechanical relationship was proposed to describe the activation of shear forces generated by the brace. In this model, the induced shear force assumed to be proportional to the imposed geometric misalignment between the torso segments:

Fshear = ks ⋅ Δd

where Fshear represents the intersegmental shear force, Δd denotes the imposed geometric misalignment, and ks is a shear stiffness coefficient describing the mechanical response of the orthotic system.

Experimental measurements obtained under the three tested misalignment conditions showed a clear increase in shear force with increasing geometric asymmetry. Under nominal alignment (D0 = 0 mm), the measured shear force remained low, with values around 30 N. When a 2 mm misalignment (D2) was introduced, shear forces increased substantially, reaching approximately 120 N. Under maximal misalignment (D4 = 4 mm), peak shear values reached approximately 170 N.

**Figure 9:**
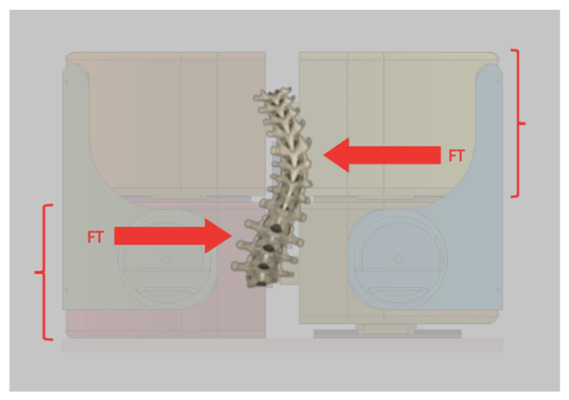
Instrumented torso model used for brace-force measurements. The figure shows the four-segment torso representation mounted on linear guides and instrumented with compression and shear load cells. The system is used to measure global compression and shear forces generated by the brace under controlled misalignment conditions. The red arrows indicate the transverse force, whose torque produces a rotation of the column. The red brackets represent the vertical distance between the two forces.

Linear regression analysis of the experimental data yielded an estimated shear stiffness coefficient of approximately ks ≈ 35–40 N/mm. The model demonstrated a high coefficient of determination ($R^{2}\;$> 0.94), indicating a strong linear relationship between imposed misalignment and shear force generation within the tested activation range, as illustrated in Figure 9.

The linear shear–misalignment relationship proposed in this study should be interpreted as a first-order mechanical model derived from controlled experimental measurements rather than as a full representation of brace–torso biomechanics.

The aim of the model is to describe the global mechanical behavior of the orthotic system observed in the instrumented test apparatus, where force components are measured under controlled geometric misalignment conditions. The experimental setup is designed to resolve global force components—upper compression, lower compression, and inter-level shear—rather than local pressure distributions at the brace–body interface.

Accordingly, the model does not explicitly account for non-linear contact behavior, frictional interactions, local pressure distribution, soft tissue deformation, or the discrete tightening mechanics of the ratchet system. These factors may influence force transmission under real brace-wearing conditions but fall outside the scope of the present mechanical characterization, which focuses on global force generation and load redistribution mechanisms.

The interaction between the brace and the torso is therefore represented at a system level, where the trunk is modeled as a segmented structure and the measured forces represent resultant mechanical interactions between brace segments and trunk segments rather than localized interface pressures.

The purpose of the present model is not to reproduce the full biomechanical complexity of the human spine, but to provide a first-order mechanical interpretation of experimentally measured forces and to establish a quantitative relationship between controlled geometric asymmetry and shear force generation.

Future work should extend the present framework by incorporating non-linear contact modeling, pressure distribution analysis, soft tissue interaction, and subject-specific geometries in order to provide a more detailed representation of brace–torso biomechanics. The present study should therefore be interpreted as a system-level experimental mechanics investigation of force generation and redistribution in an orthotic system, rather than a detailed biomechanical simulation of brace–torso interaction.

### 3.6. Compressive Load Modeling

The total compressive force generated by the brace was defined as the sum of the upper and lower compressive components acting on the torso compartments:

F total = F upper + F lower

**Figure 10:**
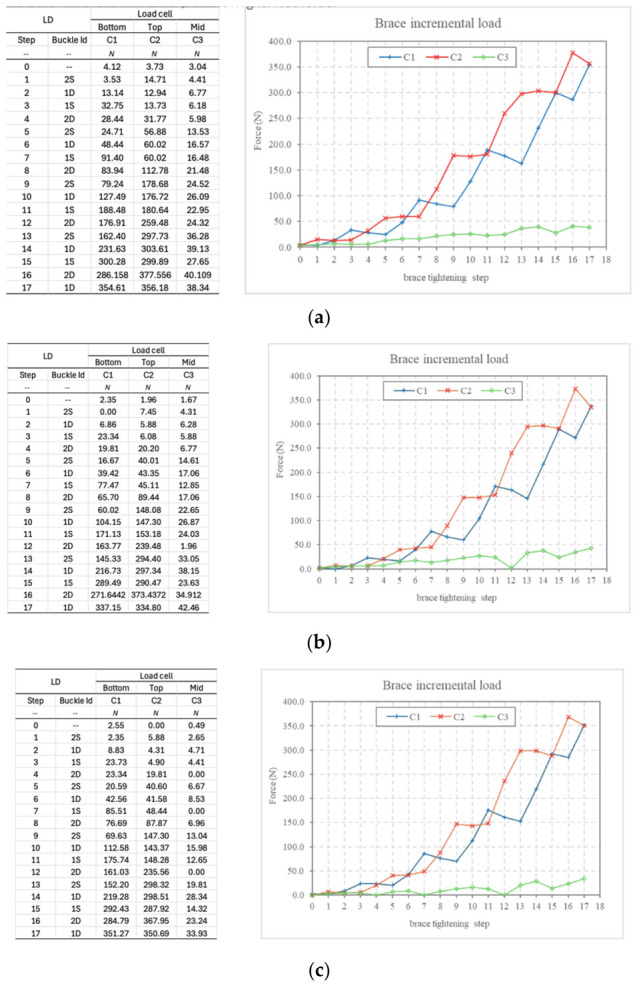
(**a**–**c**) Compressive force evolution during sequential tightening under nominal alignment (D0) using CCC50 load cells. The -- symbol indicates that no buckle has been activated at this stage. This is the initial reference condition, used as a baseline before applying the load.

Based on the experimental measurements, the maximum total compressive force recorded during tightening reached approximately 650–700 N, as illustrated in Figure 10.

However, the distribution of compressive forces between the upper and lower compartments was not constant and varied depending on the imposed misalignment condition. Under nominal alignment (D0), the compressive load was distributed relatively symmetrically between the two compartments. When a 2 mm misalignment (D2) was introduced, a moderate redistribution of compressive forces was observed, reflecting the onset of asymmetric mechanical engagement.

Under maximal misalignment (D4), the early stages of tightening were characterized by a shear-dominant phase, during which compressive forces were initially reduced while shear forces increased rapidly. As tightening progressed, compressive forces gradually increased, resulting in a combined compression–shear loading condition.

These observations indicate that geometric asymmetry modifies not only the magnitude of shear forces but also the temporal distribution of compressive loads during brace tightening.

### 3.7. Statistical Analysis

For each experimental condition, force measurements were repeated multiple times under identical mechanical conditions in order to evaluate measurement repeatability and variability. The number of repetitions represents technical replicates of a controlled mechanical experiment rather than independent biological samples.

For each measured variable (upper compression force, lower compression force, and shear force), mean values and standard deviations (mean ± SD) were calculated. Measurement repeatability was assessed using the coefficient of variation (CV), defined as the ratio between standard deviation and mean value.

To evaluate the effect of geometric misalignment on shear force generation, a one-way analysis of variance (ANOVA) was performed across the three misalignment conditions (D0, D2, and D4). Post hoc pairwise comparisons were conducted using Tukey’s test to identify statistically significant differences between conditions. Statistical significance was set at *p* < 0.05.

In addition, linear regression analysis was performed to evaluate the relationship between imposed geometric misalignment and the resulting shear force. The coefficient of determination (R^2^) was used to quantify the strength of the relationship.

To provide an estimate of measurement uncertainty, 95% confidence intervals were calculated for all measured force components. The uncertainty associated with derived quantities, such as rotational moment, was estimated using standard error propagation methods based on the variability of the measured forces and the uncertainty in the lever arm estimation.

Sensitivity analysis of the shear–misalignment relationship was performed analytically by evaluating the partial derivatives of the shear force and rotational moment equations with respect to geometric misalignment and shear stiffness parameters. This analysis allowed the evaluation of the influence of geometric asymmetry on the resulting mechanical quantities.

Statistical analyses were performed to evaluate measurement repeatability, variability, and the dependence of shear force generation on geometric misalignment under controlled mechanical conditions.

### 3.8. Mechanical Stabilization of the Fastening System

The mechanical behavior of the fastening mechanism was evaluated through repeated loading cycles in order to assess the stability of the tightening system. During the first loading cycle, the mechanism exhibited a peak force of approximately $F_{max,1}$ = 373 N. However, subsequent cycles showed a progressive reduction in the maximum force until reaching a stabilized plateau of approximately Fstable ≈ 300 N.

This reduction in peak force is attributed to the mechanical settling of the components and material accommodation within the tightening system, which occur during the initial loading cycles. After this initial adjustment phase, the system reached a stable operational condition characterized by repeatable load levels.

The decay of the maximum force over successive cycles was modeled using an exponential stabilization function:

$$F_{n\;}={\;F}_{stable}+\;(F_{max,1}-\;{\;F}_{stable})e^{-\lambda n}$$

where *n* represents the loading cycle number, and λ is an empirically derived decay constant describing the rate of stabilization. Based on the experimental observations, the value of λ was estimated to lie in the range of 0.8–1.1

This model provides a simplified description of the mechanical stabilization process occurring in the fastening mechanism during repeated tightening cycles.

### 3.9. Biomechanical Model of Induced Vertebral Moment

To extend the mechanical validation beyond force quantification, a simplified biomechanical model was developed to estimate the corrective rotational moment generated by induced shear forces.

Considering a vertebral segment subjected to anterior shear force ${\;F}_{shear}$, the resulting corrective moment M around the vertebral center of rotation can be expressed as

$$M={\;F}_{shear}\cdot h$$

where M is the induced rotational moment (Nm), ${\;F}_{shear}$ is the measured intersegmental shear force (N) and h is a perpendicular lever arm between applied shear force and vertebral center of rotation (m).

The rotational moment model is based on simplified and idealized boundary conditions and does not account for muscular activity, ligamentous constraints, or subject-specific anatomical variability. Therefore, the estimated moments should be interpreted as first-order mechanical approximations rather than physiological predictions of in vivo spinal behavior.

### 3.10. Lever Arm Estimation

The effective lever arm used for biomechanical modeling was estimated based on typical thoracic anthropometric dimensions reported in the literature. The mean transverse thoracic depth is generally reported to range between 120 and 160 mm, representing the approximate distance between the anterior thoracic surface and the vertebral center of rotation.

The effective lever arm was estimated based on typical thoracic anthropometric dimensions, where the transverse thoracic depth is generally reported in the range of approximately 120–160 mm. A conservative mid-range value of h = 0.10 mh = 0.10 m was adopted as a simplified geometric approximation of the distance between the applied shear force and the vertebral center of rotation.

It should be noted that this parameter is not derived from subject-specific anatomical measurements and does not account for individual variability in thoracic geometry. Therefore, the lever arm should be interpreted as an approximate value used for first-order mechanical estimation

### 3.11. Conceptual Biomechanical Modeling Framework

To provide a mechanical interpretation of the experimentally measured forces, a simplified analytical biomechanical model was developed. The purpose of this model is not to reproduce patient-specific anatomical conditions or to predict clinical outcomes, but to provide a first-order mechanical interpretation of the forces generated by the brace under controlled experimental conditions.

The model represents a simplified thoracic spinal segment subjected to an externally applied shear force generated by asymmetric brace loading. The spinal segment is schematized as a simplified mechanical system consisting of a vertebral body and intervertebral disk modeled using linear elastic behavior. Small-displacement assumptions were adopted in order to allow analytical interpretation of the induced rotational effects.

In the model configuration, the inferior boundary of the segment is assumed as fixed, while the applied shear force acts at an estimated lever arm distance from the vertebral center of rotation. This configuration allows estimation of the rotational moment generated by the measured shear force according to

M = Fshear · h

where F_shear is the experimentally measured shear force and h is the estimated lever arm distance.

Material properties assigned to the spinal components were selected from commonly reported values in thoracic spine biomechanical literature. Cortical bone was modeled with an elastic modulus in the range of 12–17 GPa and Poisson’s ratio of 0.3, cancellous bone with elastic modulus between 100 and 500 MPa and Poisson’s ratio of 0.2, and the intervertebral disk with elastic modulus between 4 and 8 MPa and Poisson’s ratio between 0.4 and 0.45. These values represent physiological mid-range approximations used to obtain a reasonable mechanical representation of the thoracic spinal segment.

The model is therefore intended as a conceptual mechanical framework linking experimentally measured shear forces to potential rotational moments acting on the spinal segment. The objective is to evaluate the mechanical plausibility of shear-induced rotational effects rather than to provide a subject-specific biomechanical simulation.

This simplified analytical framework provides a mechanical basis for interpreting the experimental measurements and for estimating the order of magnitude of rotational moments generated by asymmetric brace loading under controlled mechanical conditions.

## 4. Results

### 4.1. Compression–Shear Interaction

**Figure 11:**
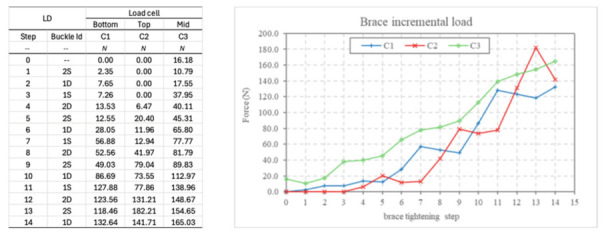
Force evolution during tightening under maximal misalignment (D4). Shear forces dominate the initial tightening phase. The -- symbol indicates that no buckle has been activated at this stage. This is the initial reference condition, used as a baseline before applying the load.

Under the D4 misalignment condition, corresponding to the maximum imposed asymmetry, the interaction between compressive and shear forces exhibited a characteristic evolution during the tightening process (Figure 11). In the initial tightening phase, the mechanical response of the system was dominated by shear forces, while the compressive components remained relatively low. As tightening progressed through successive latch positions, compressive forces gradually increased, leading to a dynamic redistribution between shear and compression components.

The total force acting on the system can be expressed as the resultant of the compressive and shear components:

$$F\;resultant=\;\sqrt{F^{2}compression+F^{2\;}shear}$$

Based on the experimentally measured peak values, the magnitude of the resultant force reached approximately 380–400 N. These observations indicate that, under conditions of significant geometric asymmetry, the brace generates a combined loading condition in which shear forces play a substantial role in the overall mechanical interaction.

### 4.2. Predictive Model Validation

The predictive mechanical model describing shear activation was validated by comparing experimentally measured shear forces with those predicted by the shear–misalignment relationship. The comparison showed a prediction error below 8%, indicating good agreement between the experimental measurements and the analytical model.

Within the tested range of geometric misalignment (0–4 mm), the model accurately captured the increase in shear forces, confirming that the proposed relationship provides a reliable description of shear force generation within the experimental activation range.

### 4.3. Estimated Corrective Rotational Moment

Using the experimentally measured peak shear forces, the corresponding corrective rotational moments acting on the vertebral segment were estimated using the previously defined lever arm relationship.

Under nominal alignment (D0 = 0 mm), the measured shear force was approximately 30 N, corresponding to an estimated rotational moment of approximately 3 Nm.

When a 2 mm misalignment (D2) was introduced, the shear force increased to approximately 120 N, producing an estimated rotational moment of approximately 12 Nm.

Under maximal misalignment (D4 = 4 mm), the measured shear force reached approximately 170 N, corresponding to an estimated rotational moment of approximately 17 Nm. These values indicate a progressive increase in the rotational moment generated by the brace as geometric asymmetry increases.

### 4.4. Result Interpretation

The estimated corrective rotational moment showed a clear dependence on the imposed geometric misalignment. Based on the shear–misalignment model previously described, the rotational moment can be expressed as

M = (ks ⋅ h) ⋅ Δd

where ks represents the shear stiffness coefficient and h the effective lever arm.

Given the experimentally derived value of ks ≈ 35–40 N/mm and the adopted lever arm value of h = 0.10 m, the resulting relationship indicates that the induced rotational moment increases approximately according to

M ≈ 3.5–4.0 Nm/mm ⋅ Δd

This implies that each millimeter of induced geometric asymmetry generates approximately 3.5–4.0 Nm of corrective rotational moment, highlighting the strong mechanical sensitivity of the system to imposed misalignment.

### 4.5. Resultant Force Vector and Mechanical Efficiency

The total corrective force generated by the brace can be described as the resultant of the compressive and shear components:

$$F\;resultant=\;\sqrt{F^{2}\;compression+F^{2}\;shear}$$

Under maximal misalignment (D4), the experimentally measured values were approximately Fcompression ≈ 320 N and Fshear ≈ 170 N, resulting in a resultant force magnitude of approximately 360–380 N.

The ratio between shear and compressive forces can therefore be expressed as

$$\frac{F\;shear}{F\;compression}\approx 0.5$$

This indicates that up to 50% of the corrective force vector may be attributable to shear contribution under maximal asymmetry. This finding is mechanically relevant, as traditional rigid braces are generally considered compression-dominant systems in which shear forces are often assumed to be negligible or unquantified.

### 4.6. Comparison with Biomechanical Literature

Finite element models and biomechanical simulations of thoracic scoliosis have reported corrective rotational moments typically ranging between 5 and 20 Nm, depending on curve magnitude, thoracic stiffness, rib cage coupling, and boundary conditions.

The estimated corrective moments generated by the Canali Front-Push Brace, ranging from 3 to 17 Nm, fall within this biomechanically relevant range. These results suggest that the induced shear forces observed experimentally are not merely secondary effects but may represent mechanically meaningful contributors to vertebral rotational correction.

### 4.7. Theoretical Influence on Cobb Angle Reduction

Although the present study does not include direct radiographic measurements, a simplified approximation can be used to estimate the potential rotational influence generated by the induced moments. Assuming small-angle behavior, the angular displacement can be approximated as

$$\theta \;\approx \;\frac{M}{K\theta }$$

where θ represents the angular rotation, M the induced moment, and Kθ the rotational stiffness of the thoracic segment.

Biomechanical studies report thoracic rotational stiffness values in the range of approximately 20–60 Nm/rad. Using the estimated moment for D4 (M ≈ 17 Nm), the resulting angular displacement can be approximated as

θ ≈ 0.28–0.85 rad

When scaled to the local vertebral segment, this corresponds to a theoretical rotational influence of approximately 1.5–5°. While this simplified estimation does not imply direct Cobb angle reduction, it suggests the mechanical plausibility of rotational modulation generated by the brace.

### 4.8. Vector Decomposition of Corrective Forces

The corrective force generated by the brace can be decomposed into its compressive and shear components according to

F→=Fcz^+Fs x^

where Fc represents the compressive component and Fs the shear component. The magnitude of the resultant force vector is therefore given by

$$|F^{\to }|\;=\sqrt{F^{2}c+F^{2}}s$$

The angle of force application relative to the compressive axis can be expressed as

$$\alpha ={tan}^{-1}\left(\frac{Fs}{Fc}\right)$$

Under maximal misalignment (D4), using the experimentally measured values (170 N shear and 320 N compression), the resulting angle is approximately

α ≈ 28°

This indicates that the brace generates a force vector deviating approximately 28° from pure compression, demonstrating a substantial shear contribution to the overall corrective force.

### 4.9. Comparative Mechanical Framework

Traditional thoraco-lumbo-sacral orthoses are generally characterized by compression-dominant mechanical behavior, which can be approximated as

F ≈ Fc

In such systems, the contribution of shear forces is often assumed to be minimal or remains unquantified. In contrast, the brace investigated in the present study generates a combined corrective force described by

F = Fc + Fs

with a shear-to-compression ratio reaching approximately Fs/Fc ≤ 0.5 Fs/Fc ≤ 0.5 under maximal asymmetry.

Using the experimentally measured peak shear force (170 N) and the estimated lever arm (0.10 m), the induced rotational moment reaches approximately 17 Nm. Assuming thoracic rotational stiffness values of 20–60 Nm/rad, the resulting angular displacement can be estimated as

$$\theta =\frac{M}{K\theta }$$

This leads to theoretical rotational values consistent with those observed in biomechanical simulation studies. These results indicate that the brace operates as a hybrid vectorial corrective system, combining compressive and shear forces to generate rotational moments within physiologically relevant ranges.

### 4.10. Stress Magnitude Plausibility

The magnitude of shear stress applied to the trunk can be estimated using the following relation:

$$\tau \;=\;\frac{F}{A}$$

where F represents the applied shear force and A the effective contact area. Assuming a contact area in the range of 0.015–0.025 m^2^, the resulting shear stress is estimated to lie between 6 and 11 kPa.

These stress levels remain well below thresholds associated with tissue damage and are consistent with therapeutic loading ranges typically reported in brace treatment literature, suggesting that the mechanical forces generated by the brace remain within physiologically tolerable limits.

### 4.11. Confidence Interval

In addition to reporting mean values and standard deviations, 95% confidence intervals (CIs) were calculated for all measured force components in order to provide an estimate of measurement uncertainty and experimental variability. Confidence intervals were calculated using the standard formulation:

$$\mathrm{CI}\;=\;x^{-}\;\pm \;t\cdot x=\frac{SD}{\sqrt{n}}$$

where x− represents the sample mean, SD represents the standard deviation, nn represents the number of technical replicates, and t represents Student’s *t*-value corresponding to the 95% confidence level.

The uncertainty associated with the estimated rotational moment was evaluated using standard error propagation principles. Considering the relationship

M = Fshear ⋅ h

the propagated uncertainty can be approximated as

$$\sigma M=(\sqrt{(h\cdot \sigma F)2+(Fshear\cdot \sigma h)2}$$

where σF represents the variability in the measured shear force and σh represents the uncertainty associated with the lever arm estimation. This formulation provides a first-order estimation of uncertainty in the derived biomechanical quantities and allows interpretation of the estimated moments as order-of-magnitude values rather than exact predictions. A sensitivity analysis of the proposed shear–misalignment relationship

Fshear = ks ⋅ Δd

was also performed analytically. The partial derivatives of the model are

$$\frac{\partial Fshear}{\partial ks}=\Delta d$$

$$\frac{\partial Fshear}{\partial \Delta d}=\mathrm{ks}$$

These relationships indicate that shear force varies linearly with both the shear stiffness coefficient and the imposed geometric misalignment, meaning that small variations in misalignment produce proportional changes in shear force.

Similarly, for the rotational moment derived from the model

M = ks ⋅ h ⋅ Δd

$$\frac{\partial M}{\partial \Delta d}=\mathrm{ks}\cdot h$$

This confirms the strong dependence of the estimated rotational moment on geometric asymmetry and highlights the importance of controlled misalignment in the activation of shear-induced rotational effects.

## 5. Discussion

The present study demonstrates that controlled geometric asymmetry within the orthotic system can modulate the distribution of corrective forces, resulting in a transition from a predominantly compression-based configuration toward a hybrid compression–shear mechanical regime. The experimental results showed a consistent and reproducible behavior, with shear force increasing proportionally with imposed misalignment and exhibiting strong linearity (R^2^ > 0.94) within the investigated range.

Under maximal misalignment conditions, the shear-to-compression ratio approached approximately 50%, indicating that shear represents a substantial component of the overall force system generated by the brace. In parallel, the estimated rotational moments reached values up to approximately 17 Nm, while the resultant force vector deviated by about 28° from pure compression. These findings indicate that the orthosis is capable of generating multi-directional force components within ranges that remain compatible with physiologically tolerable loading conditions. However, these results should be interpreted within the context of controlled mechanical analysis and do not constitute direct evidence of clinical efficacy.

Importantly, this study provides a quantitative biomechanical characterization of force generation and interaction, addressing a gap in the existing literature, where the corrective action of spinal orthoses is often described qualitatively rather than through experimentally measured and modeled force components.

From a mechanistic perspective, the findings suggest that the corrective behavior of the investigated brace may differ from the classical three-point pressure paradigm commonly described in scoliosis bracing. Conventional orthoses are primarily designed to generate lateral forces targeting deformity in the frontal plane. In contrast, the present analysis demonstrates that controlled geometric asymmetry can induce a measurable anterior shear component, thereby introducing an additional directional component to the corrective force system.

The relationship between induced shear and rotational effect can be described, in a simplified form, by the expression

M = Fshear ⋅ h

where Fshear represents the experimentally measured intersegmental shear force and h corresponds to the effective lever arm. Based on peak shear values of approximately 170 N and a conservative lever arm estimate of 0.10 m, the resulting rotational moment may reach values on the order of 17 Nm. These estimates fall within ranges reported in biomechanical studies of thoracic spinal motion segments; however, they should be interpreted as first-order approximations derived a simplified model.

Taken together, these observations support the hypothesis that controlled anterior shear may represent a biomechanically plausible mechanism contributing to rotational modulation of the spine. Nevertheless, this interpretation remains theoretical and hypothesis-generating, and further investigation incorporating subject-specific modeling and clinical validation is required to establish its translational relevance.

### 5.1. Mechanical Energy Consideration and Mechanotransduction Perspective

The mechanical work applied during brace tightening can be approximated as the product of the induced shear force and the displacement occurring during the tightening process, expressed as W = Fshear⋅Δx, where Δx represents the displacement generated during tightening. This formulation suggests that the mechanical stimulus delivered to the trunk depends not only on the magnitude of the applied force but also on the associated displacement, highlighting the combined role of force and deformation in determining the mechanical environment experienced by the musculoskeletal structures. Such interactions may be relevant for the activation of mechanotransduction pathways, through which biological tissues respond to mechanical loading.

To further contextualize the potential translational relevance of the mechanical stimulus generated by the brace, the cumulative mechanical input delivered over time can be conceptualized as a mechanical dose defined by the relation mechanical dose = M ⋅ t, where M represents the induced rotational moment, and t corresponds to the daily application time. Assuming a daily brace usage of approximately three hours, corresponding to t = 10,800 s, and considering a maximal induced moment of approximately 17 Nm, the resulting cumulative mechanical dose can be estimated as Dose ≈ 17 × 10,800, yielding approximately 183,600 Nm⋅s. This simplified formulation introduces a quantitative framework for describing the time-distributed mechanical modulation applied to the trunk, which may be relevant for understanding long-term mechanobiological adaptation processes.

From a mechanobiological perspective, sustained sub-maximal mechanical loading within the range of approximately 3–17 Nm, as observed in the present study, may influence several adaptive processes within the thoracic spine and rib cage system. This simplified estimation does not represent patient-specific biomechanics but provides an order-of-magnitude interpretation of the measured forces. These include the potential modulation of vertebral growth patterns, remodeling of the rib cage structures, neuromotor adaptation of postural control mechanisms, and gradual modulation of axial rotational dynamics of the spine. Such interpretations are consistent with established mechanobiological principles, including those underlying the Hueter–Volkmann law [40,41], which describes the influence of mechanical loading on growth plate modulation and skeletal development. The mechanotransduction perspective presented here should be interpreted as a conceptual framework linking the observed mechanical stimuli to potential biological responses. While sustained mechanical loading in the reported range may be consistent with known mechanobiological principles (e.g., Hueter–Volkmann law), the present study does not provide direct evidence of biological adaptation or tissue remodeling. Therefore, these considerations should be regarded as hypothesis-generating and require further investigation through experimental and clinical studies.

However, preliminary radiological outcomes associated with the same orthopedic device have been previously reported in a retrospective clinical case series investigating an integrated postural reprogramming approach [42]. In that study, radiographic improvements in Cobb angle were documented in patients treated with the Canali orthopedic brace in combination with targeted neuromotor exercises.

While the present study does not include direct clinical or radiographic outcomes, radiological improvements associated with the Canali Postural Method and brace application have been previously reported in a retrospective case series, in which reductions in Cobb angle were observed following an integrated postural reprogramming approach. However, the current work is focused on the mechanical characterization of force generation and does not establish a direct cause–effect relationship between the measured forces and clinical outcomes. Rather, it provides a mechanistic framework that may contribute to the interpretation of previously observed radiological findings. Further studies integrating mechanical measurements with prospective clinical data will be required to investigate a potential dose–response relationship between shear-induced forces and scoliosis correction.

### 5.2. Limitations of the Biomechanical Model

The biomechanical model proposed in this study represents a simplified analytical framework and does not aim to reproduce the full complexity of the human spinal system. Accordingly, several limitations should be acknowledged.

First, the model assumes linear elastic material behavior and simplified boundary conditions, whereas the physiological spine exhibits non-linear, viscoelastic, and time-dependent properties, particularly at the level of intervertebral disks and surrounding soft tissues. Second, the model does not explicitly incorporate the contribution of muscles, ligaments, and rib cage interactions, all of which play a relevant role in spinal load distribution and mechanical stability. Third, the interaction between the brace and the torso is represented in a simplified manner and does not account for pressure distribution, frictional effects, or subject-specific anatomical variability. Finally, the model is not patient-specific and does not include finite element analysis or individualized geometry, which would be required for the quantitative prediction of clinical outcomes.

Despite these limitations, the proposed framework provides a first-order mechanical interpretation of the experimentally measured forces and supports the biomechanical plausibility of shear-induced rotational moments. Future investigations should integrate finite element modeling, soft tissue interaction, and subject-specific simulations to further refine the proposed approach.

A potential source of bias is that the investigated orthosis is protected by a patent held by the author. However, the experimental methodology relies on objective mechanical measurements obtained through calibrated load cells within a controlled instrumented setup, thereby minimizing subjective influence. Moreover, the mechanical framework described in this study—based on the interaction between compressive and shear components—is not device-specific and may be applicable, in principle, to other orthotic systems.

Future studies should include independent validation and direct comparison with established TLSO braces to assess the generalizability of the proposed biomechanical model. Conventional TLSO systems are typically described as compression-dominant, with corrective action primarily attributed to lateral forces. In contrast, the present study highlights the role of controlled shear components, which are not routinely quantified in standard brace designs.

The findings should be interpreted within the scope of mechanical validation and do not constitute evidence of clinical efficacy. While the measured forces and derived rotational moments support a biomechanically plausible mechanism for influencing spinal alignment, their translation into clinical outcomes, such as Cobb angle reduction, requires dedicated prospective studies.

Finally, the derived quantities—including rotational moment, angular estimation, and stress—are subject to uncertainty related to measurement variability and parameter estimation (e.g., force, lever arm, stiffness, and contact area). These values should therefore be interpreted as first-order approximations and order-of-magnitude estimates rather than precise predictive quantities. The observed relationship between induced shear forces and estimated rotational moments supports a plausible mechanical mechanism through which shear may contribute to vertebral rotation modulation; however, this interpretation remains theoretical and hypothesis-generating, rather than an established clinical effect.

## 6. Conclusions

The present study provides a quantitative mechanical characterization of a front-push orthotic system through controlled experimental measurements and simplified analytical modeling. The results demonstrate that the orthosis is capable of generating both compressive and shear forces, with compressive forces reaching approximately 370 N and shear forces up to approximately 170 N under controlled geometric asymmetry conditions.

The experimental results further show a consistent relationship between geometric misalignment and shear force generation, indicating predictable mechanical behavior within the tested activation range. Measurement repeatability was high, and the fastening mechanism exhibited mechanical stabilization after repeated loading cycles, suggesting consistent mechanical performance during repeated brace tightening.

From a biomechanical perspective, the measured shear forces and the corresponding estimated rotational moments support the mechanical plausibility of a corrective mechanism involving both compressive and shear components. The investigated orthotic system may therefore be interpreted as a hybrid compression–shear corrective system rather than a purely compression-dominant orthosis.

However, the present work represents a mechanical validation study and does not include clinical or radiographic outcome data. Therefore, the findings should be interpreted as a mechanical and biomechanical framework rather than evidence of clinical efficacy.

Future studies integrating advanced biomechanical modeling, pressure distribution analysis, subject-specific simulations, and prospective clinical investigations will be necessary to evaluate the clinical relevance and translational implications of the proposed mechanical mechanism. This study shows that brace correction may not be purely compression-driven, but may also involve shear-induced rotational mechanisms generated by asymmetric brace loading.

## 7. Patents

The orthopedic device described in this study, referred to as the Canali Front-Push Brace, is protected by a registered patent held by the author.

## Data Availability

The datasets generated during the current study are available from the corresponding author upon reasonable request.

## Supplementary Materials

The following supporting information can be downloaded at https://www.mdpi.com/article/10.3390/bioengineering13050491/s1, Characterization and Validation of a Front-Push Scoliosis Brace.
