# Supplementary material for: Mechanical Modeling and Experimental Validation of a Front-Push Orthopedic Brace: Compressive–Shear Force Characterization Under Controlled Misalignment
Source: Bioengineering (Basel). 2026 Apr 23;13(5):491. doi: 10.3390/bioengineering13050491 (PMC13203870; doi:10.3390/bioengineering13050491)
Supplement: Supplementary file 1 [file bioengineering-13-00491-s001.zip › bioengineering-4221983-supplementary.pdf]

© MaCh3D s.r.l. Proprietary & Confidential

Characterization and Validation of a Front-Push Scoliosis Brace

Experimental and numerical analysis of the forces generated by a front-push brace for scoliosis correction.

A00	27/01/2026	First release	N. Sulsenti	n.sulsenti@mach3d.it	M.Vettori
Rev	Data	Descr	Author	Contact person	Revised / approved by

© MaCh3D s.r.l. Proprietary & Confidential

Introduction and Development of the Torso

- *Design of Equipment for Functional Testing and Characterization of the Brace*

© MaCh3D s.r.l. Proprietary & Confidential

Introduction

The purpose of this document is to share the design choices related to a measurement setup for the resultant forces exerted by the brace under study.
Direct measurement of the resultant forces is useful for validating the computational model and for comparison with values already obtained from measurements using load cells.
The goal is to create a testing instrument capable of measuring the three main forces applied by the brace:

- Upper compression
- Lower compression
- Shear force between the upper and lower segments

The force measurement is intended to be performed using an instrumented torso, divided into four segments as shown in the image below.

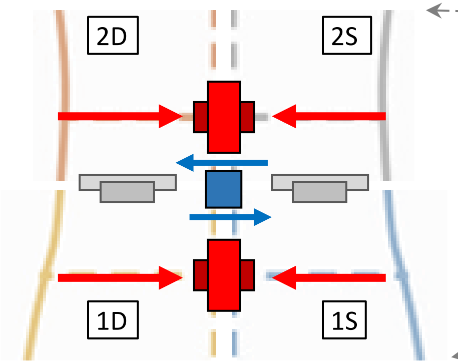

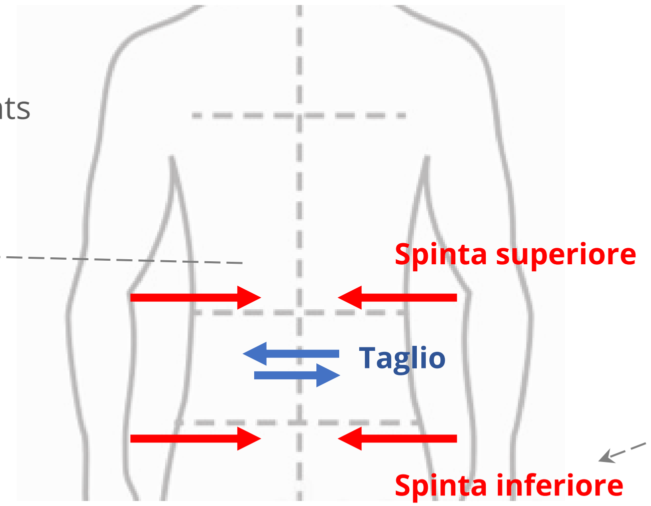

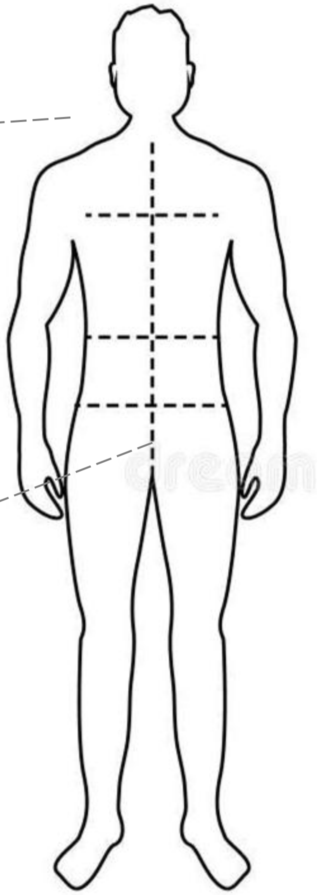

© MaCh3D s.r.l. Proprietary & Confidential

Development of the Torso

The geometry of the stylized human torso was modeled based on the internal surface profile of a CAD model of an orthopedic brace, which was itself obtained from a 3D scan of a prototype.

To enable the measurement and analysis of corrective forces in different regions, the torso was divided into four distinct sections.
Its shape was assumed to coincide with that of the brace, ensuring conformal contact between the two components (brace and torso). Consequently, the torso's cross-section was derived from the nominal geometry of the brace and extruded cylindrically along the vertical (z) axis.

The four sections were assembled while preserving the translational degrees of freedom required for accurate force measurement..

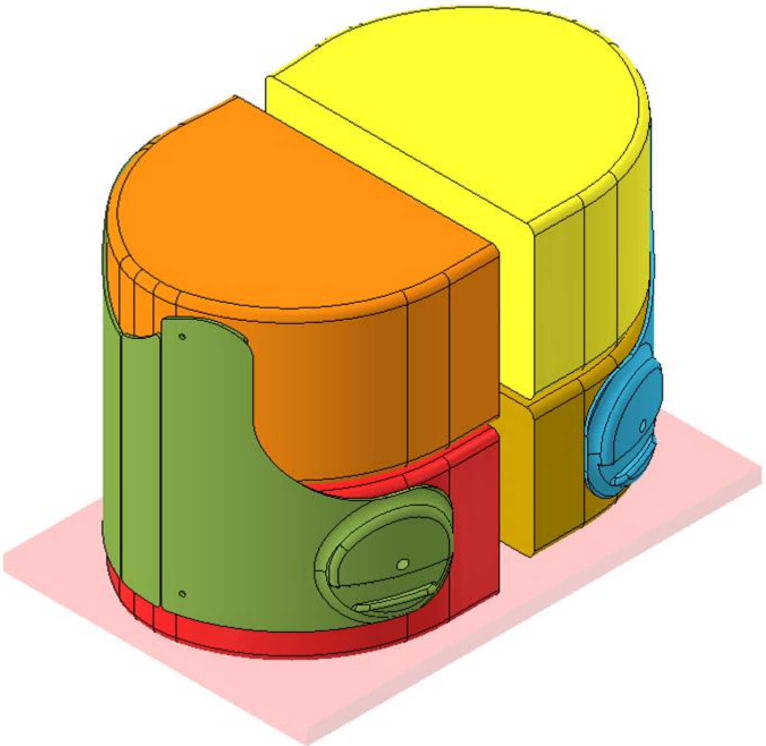

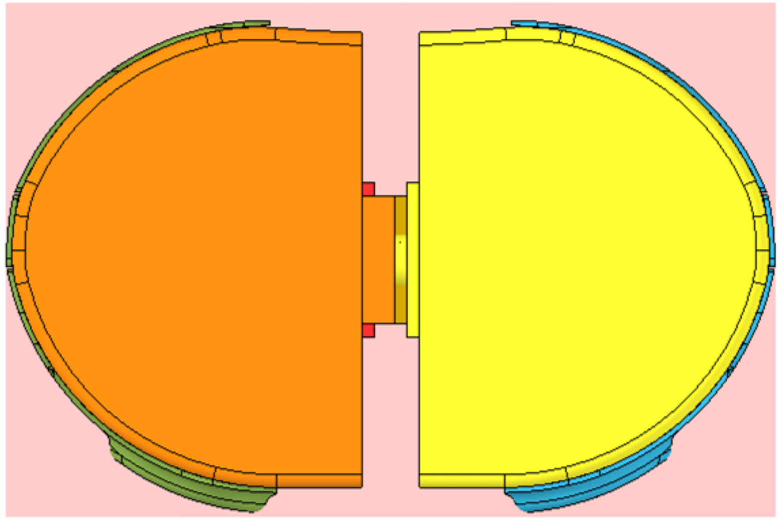

© MaCh3D s.r.l. Proprietary & Confidential

Development of the Torso

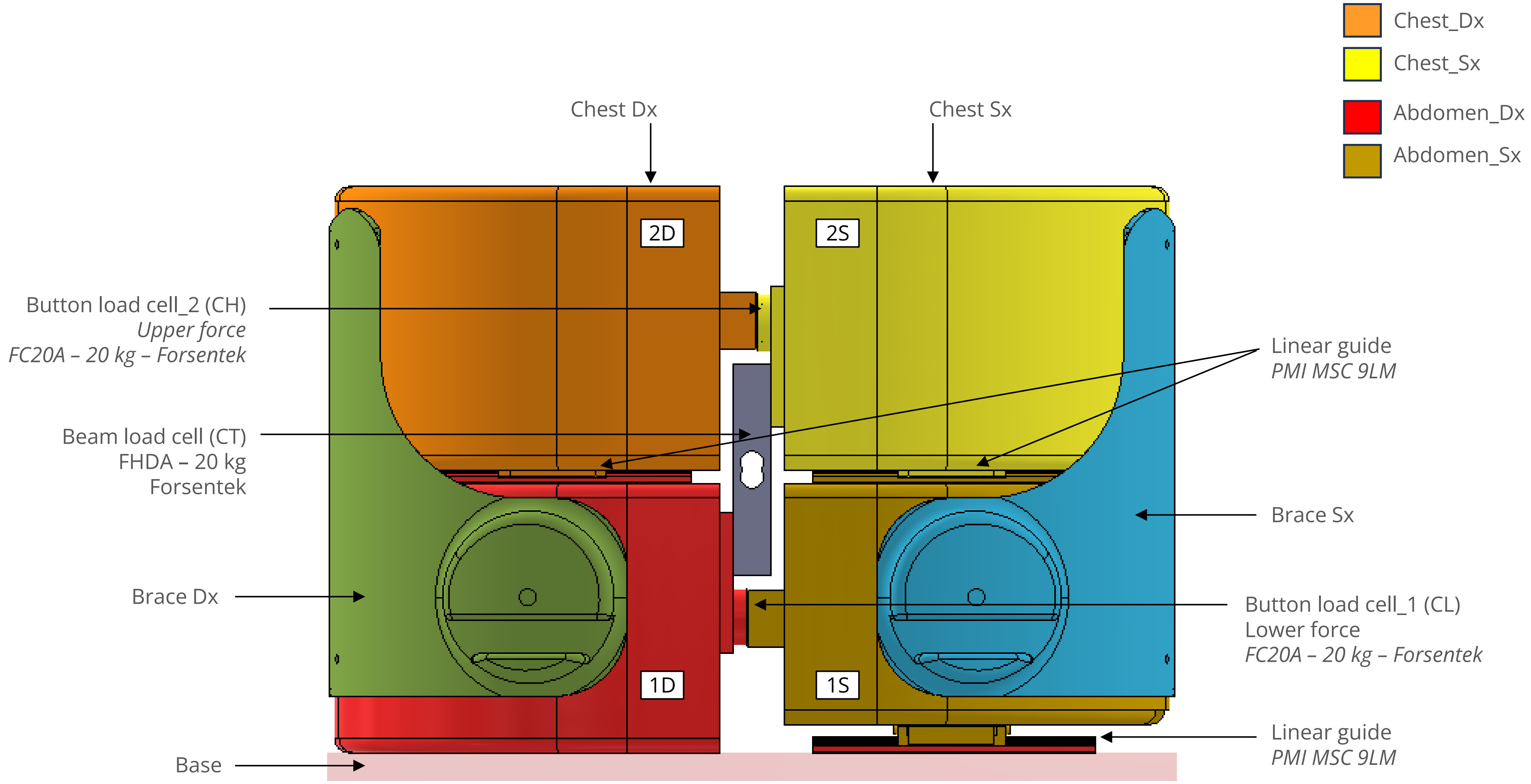

© MaCh3D s.r.l. Proprietary & Confidential

Development of the Torso

Button load cells are mounted on Plate 2, which is fixed to the chest/abdomen. Plate 1 acts as the contact plate for the button load cells and is used for force measurement.

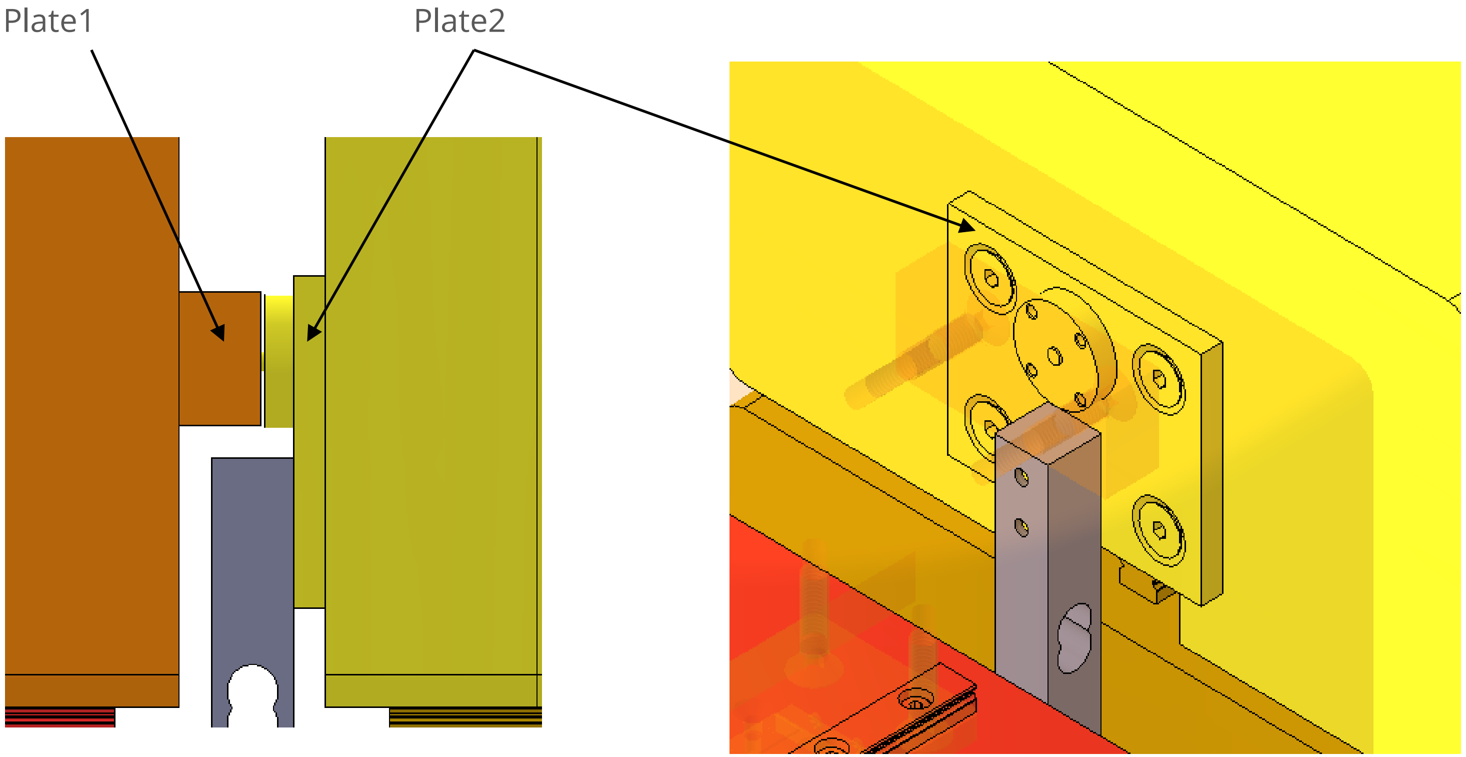

© MaCh3D s.r.l. Proprietary & Confidential

Development of the Torso

2 Button load cells: the upper cell is used to measure the force (F) between the left and right chest (Chest_Sx and Chest_Dx), while the lower cell measures the force (F) between the left and right abdomen (Abdomen_Sx and Abdomen_Dx).

1 Shear load cell: used to measure the shear force (FT) between the right chest (Chest_Dx) and the left abdomen (Abdomen_Sx).

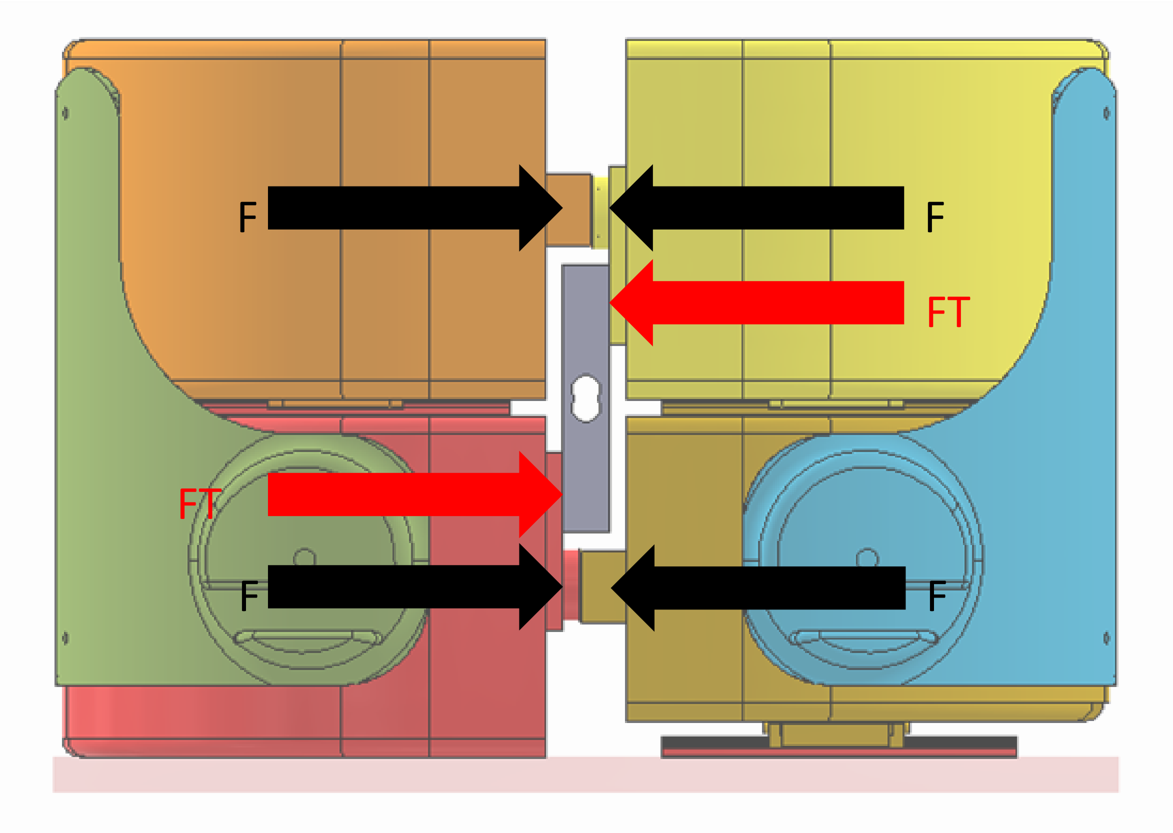

© MaCh3D s.r.l. Proprietary & Confidential

Development of the Torso

Objective: to evaluate the defined "shear" force (FT).
The contact between the torso and the brace must occur at Abdomen_Sx and Chest_Dx.

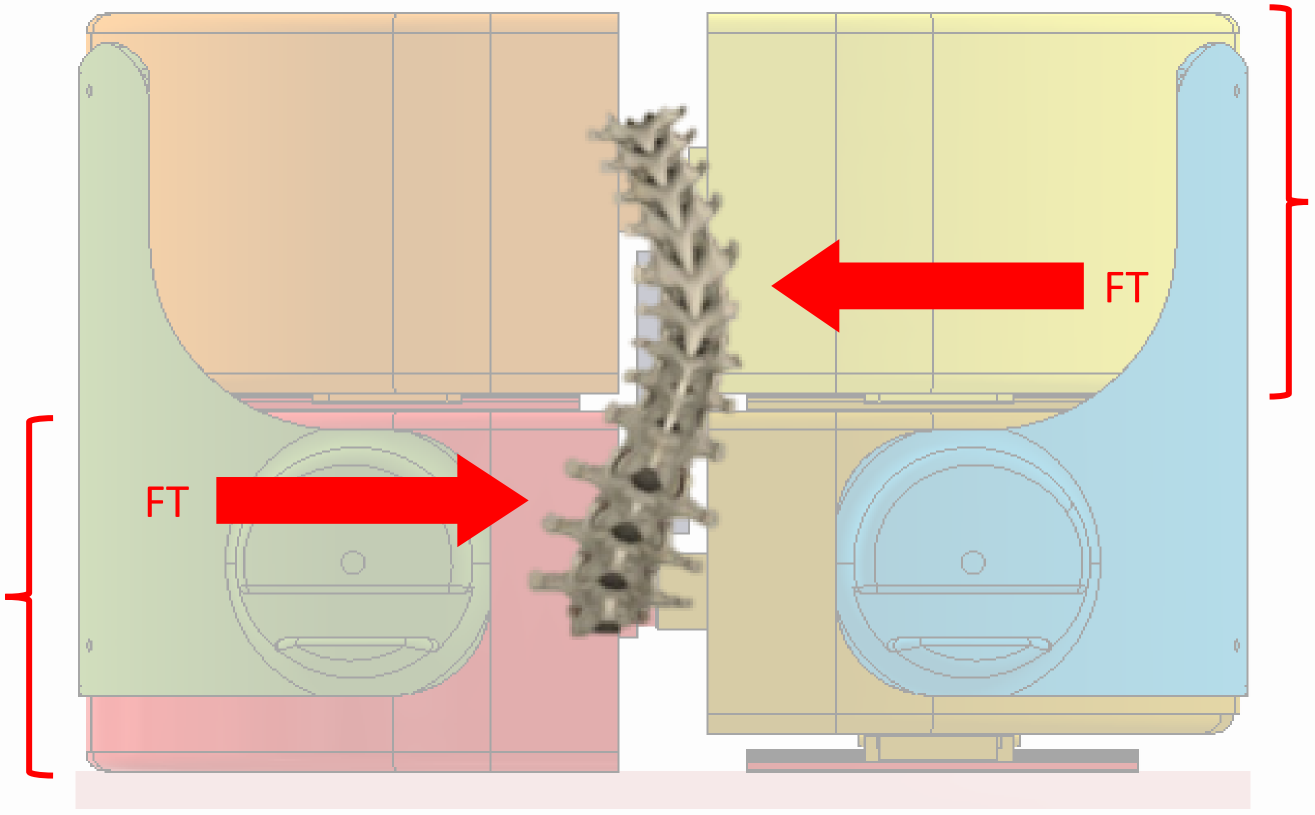

© MaCh3D s.r.l. Proprietary & Confidential

Development of the Torso

The “misalignment” required for the evaluation of FT would be obtained by inserting spacers (not shown) in the positions indicated by the arrows.

The misalignment can also be achieved by 3D-printing thin “skins” to be inserted between the torso and the brace at the desired locations. This solution, shown in the image below, is the one that was eventually adopted for the experimental tests.

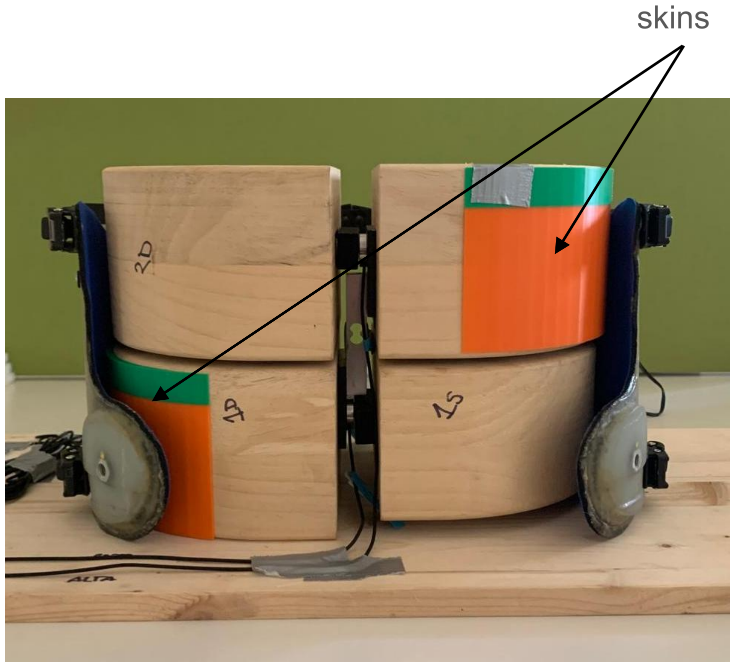

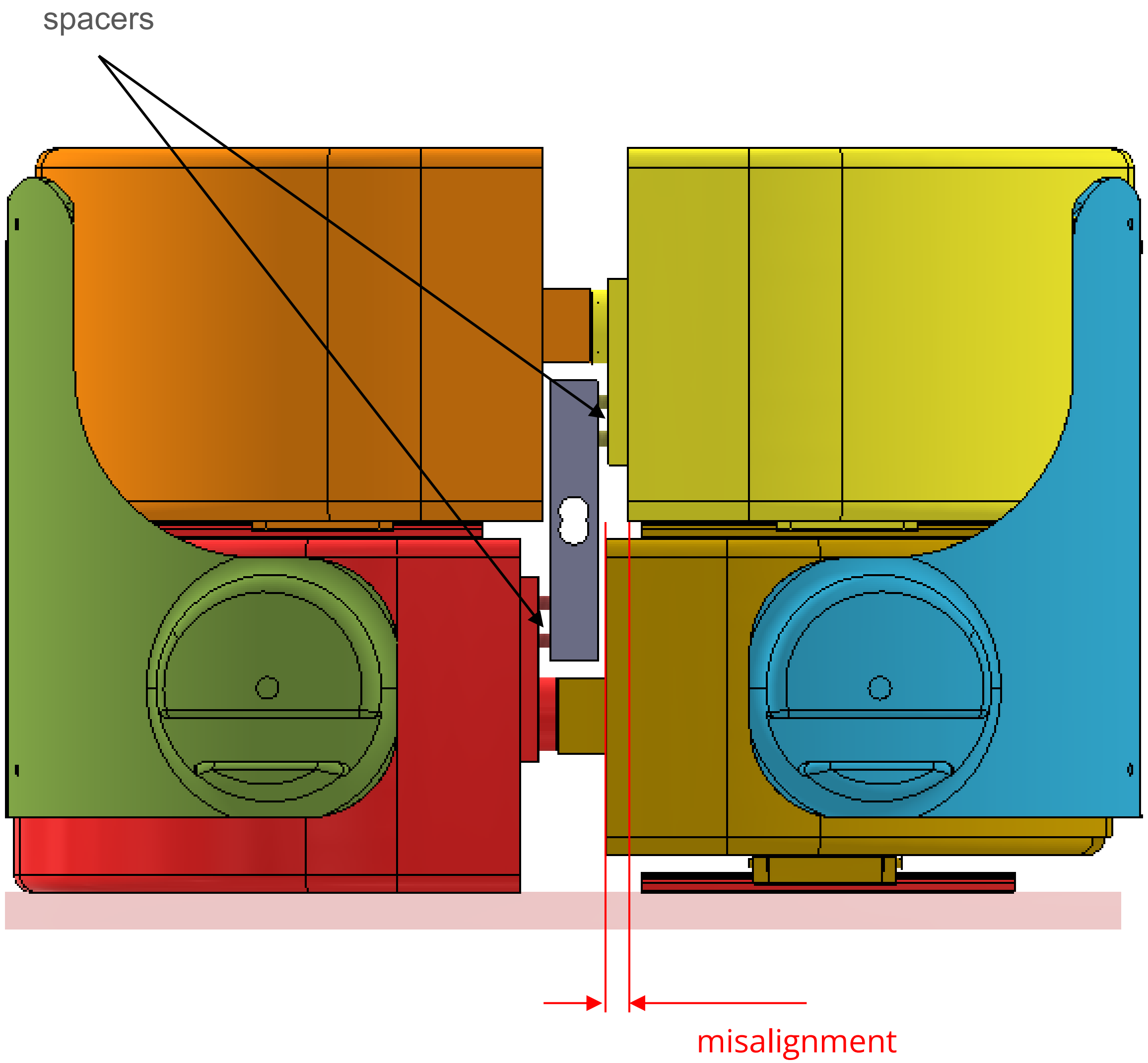

© MaCh3D s.r.l. Proprietary & Confidential

Development of the Torso

If a 20 kg full-scale load on the load cell (LC) proves insufficient, a configuration with three larger button load cells will be used.
The same solution can be applied for a 20 kg load using three identical load cells.

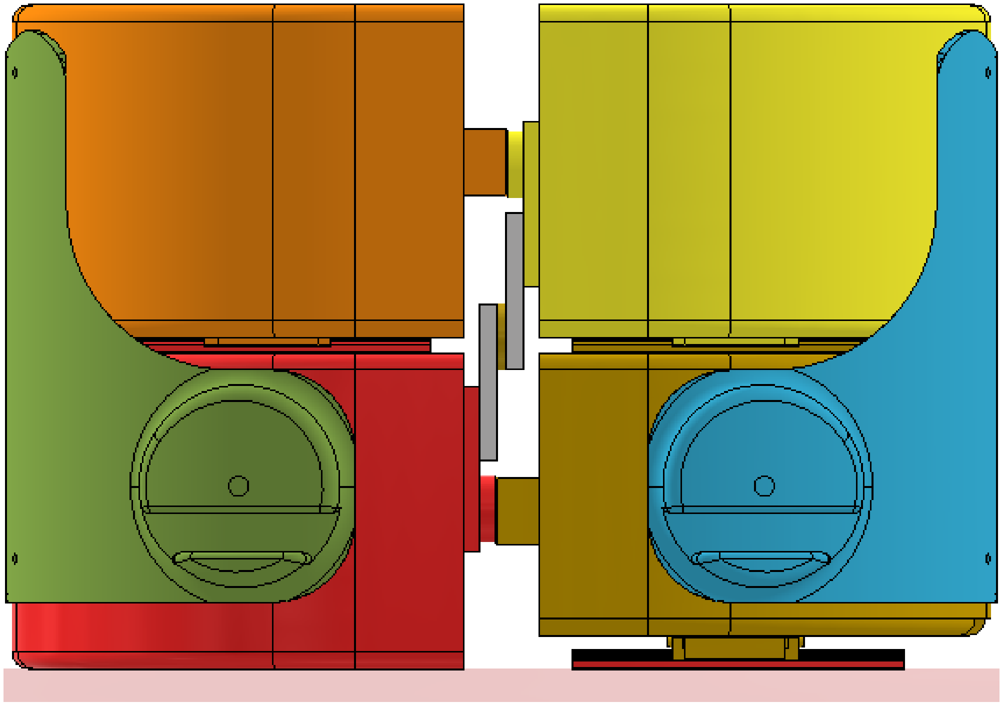

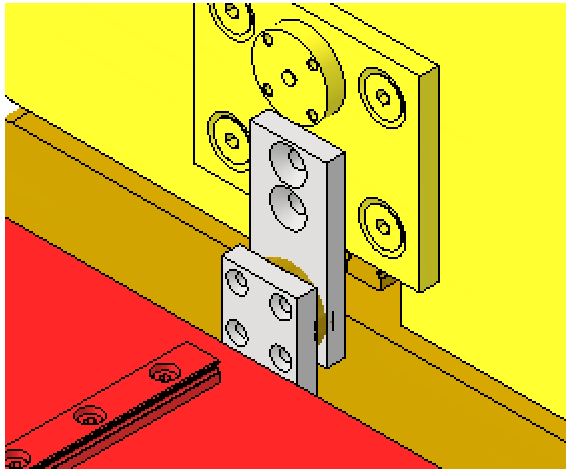

© MaCh3D s.r.l. Proprietary & Confidential

Development of the Torso

In summary, there are three design/construction options.

**(A) 2 Button load cells and 1 Beam load cell (CTC)**	Load range 200N (20kg)<br>A more accurate solution using button load cells for axial force measurement and a shear load cell for shear force measurement.
**(B) 3 Button load cells (CCC-20)**	Load range 200N (20kg)<br>The shear load cell is replaced with a button load cell operated in compression through adapter plates.<br>Subject to a small error due to plate deflection (negligible).<br>Easier to manage because all three load cells are identical.
**(C) 3 Button load cells (CCC-50)**	Same as above, but with an increased load range of 500 N (50 kg).<br>The only possible solution if a higher full-scale capacity is required.

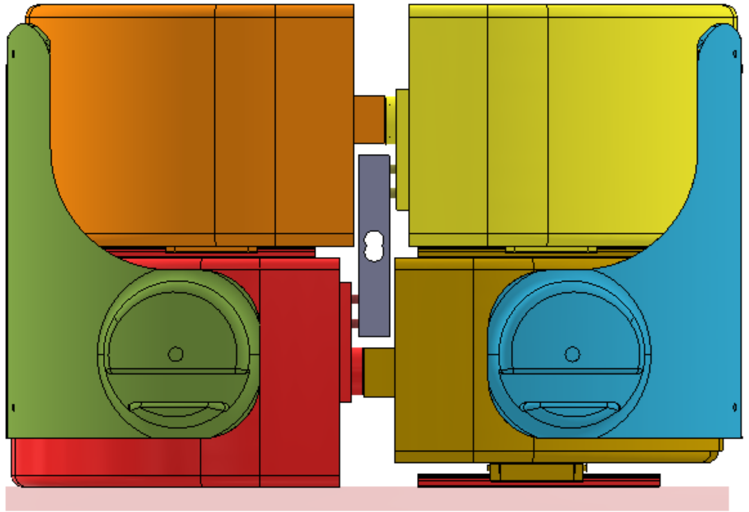

(A)

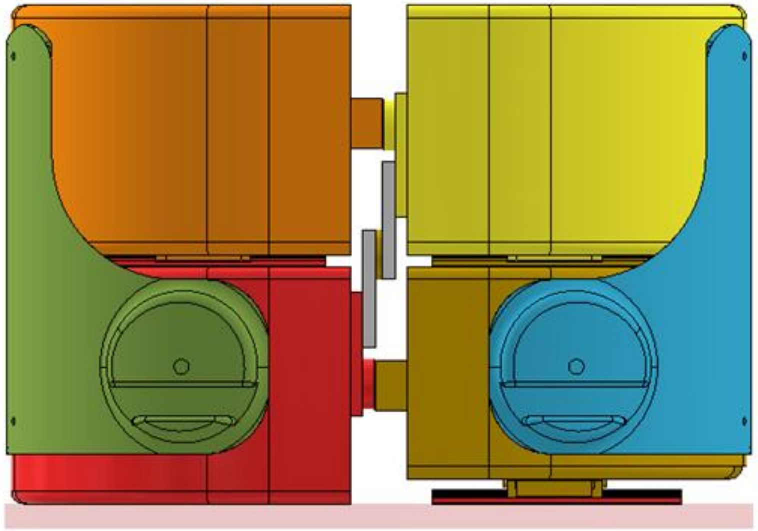

(B) e (C)

© MaCh3D s.r.l. Proprietary & Confidential

Fabrication and assembly of torso test equipment

– *Fabrication and assembly of the components that will constitute the torso for the execution of experimental tests.*

© MaCh3D s.r.l. Proprietary & Confidential

Torso assembly

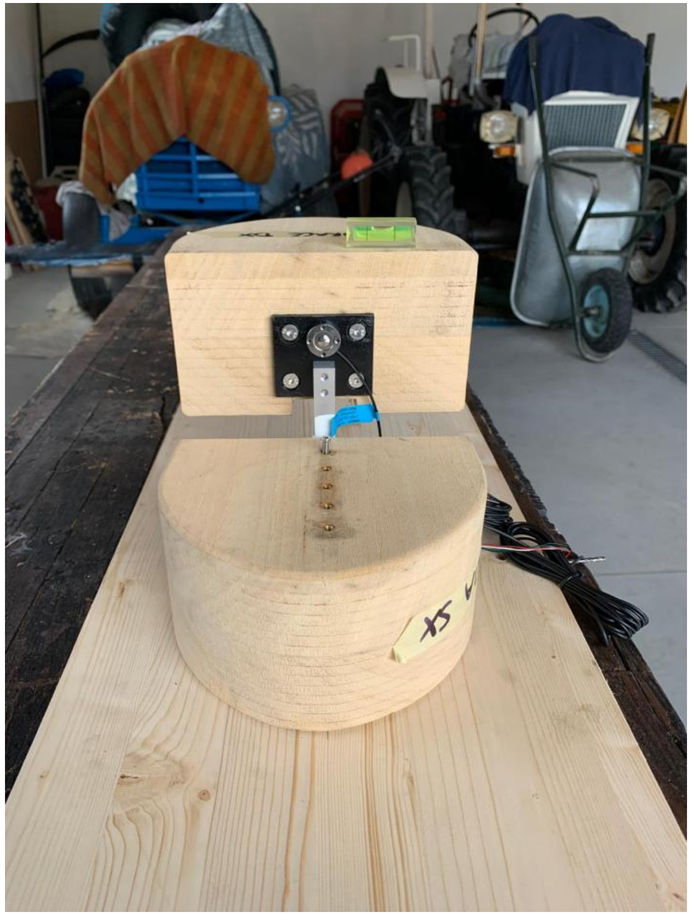

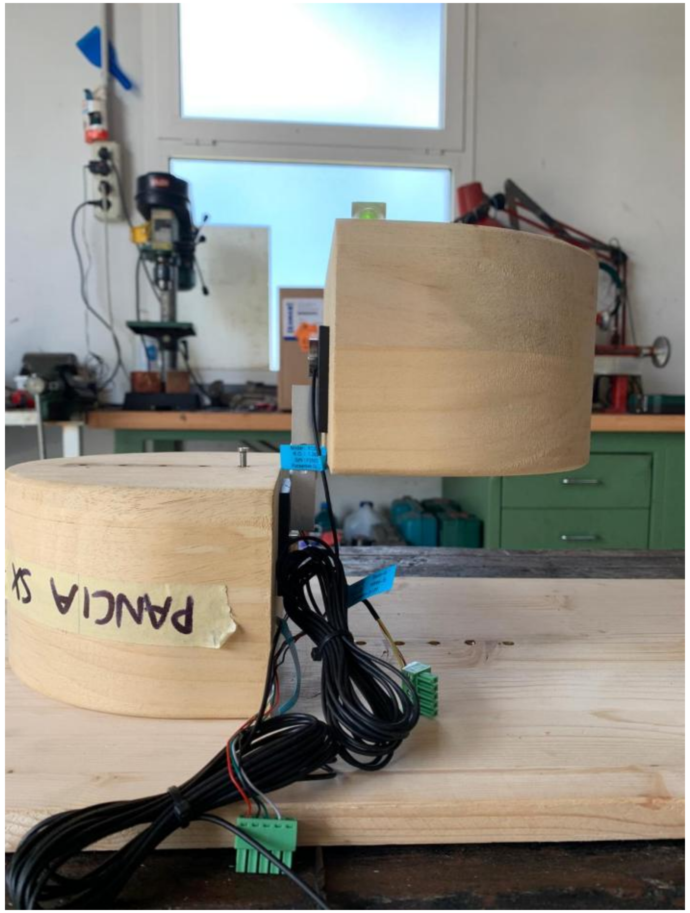

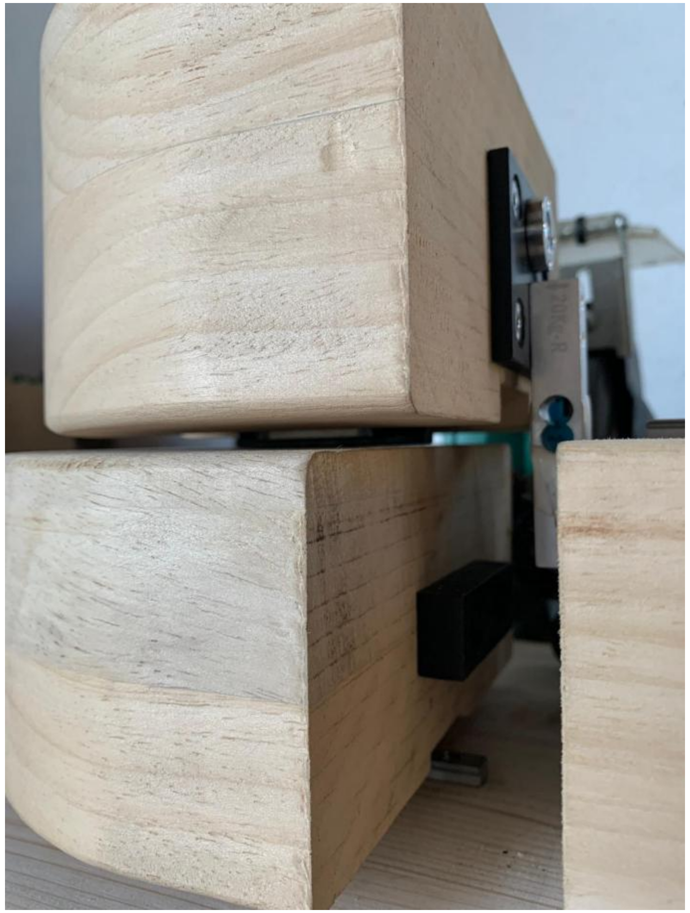

© MaCh3D s.r.l. Proprietary & Confidential

Torso assembly

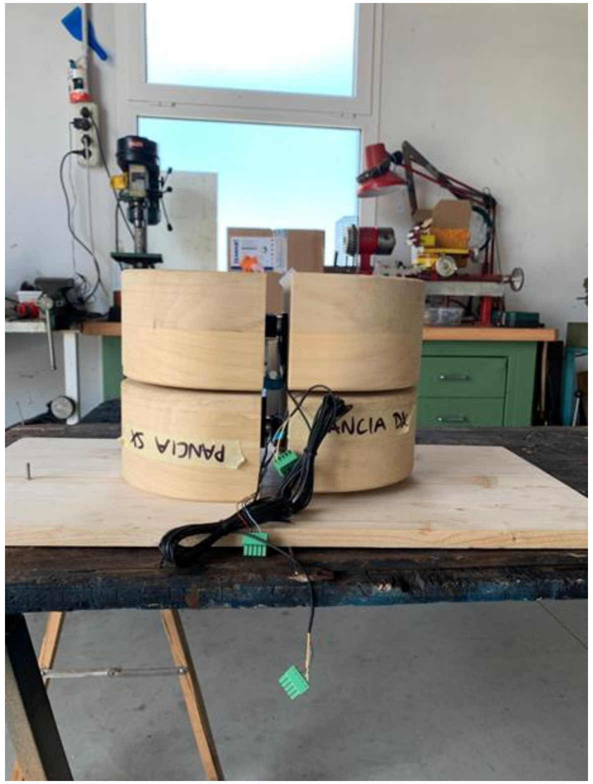

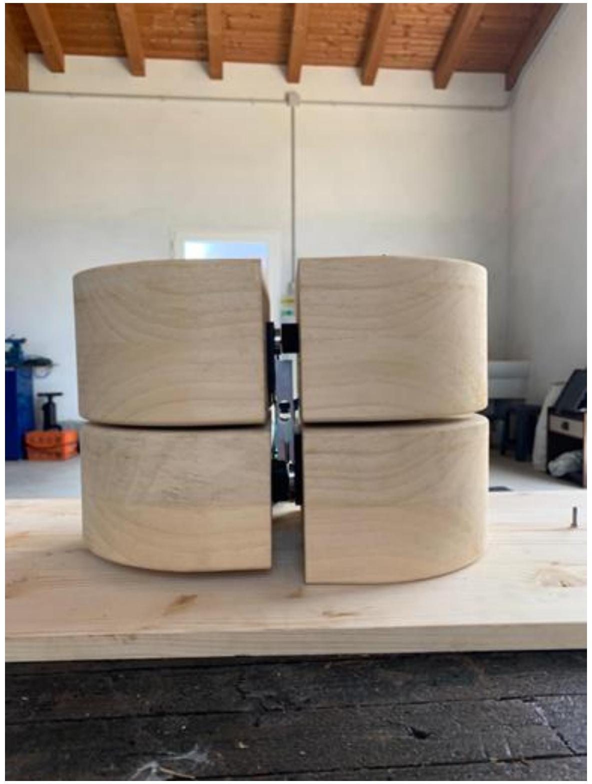

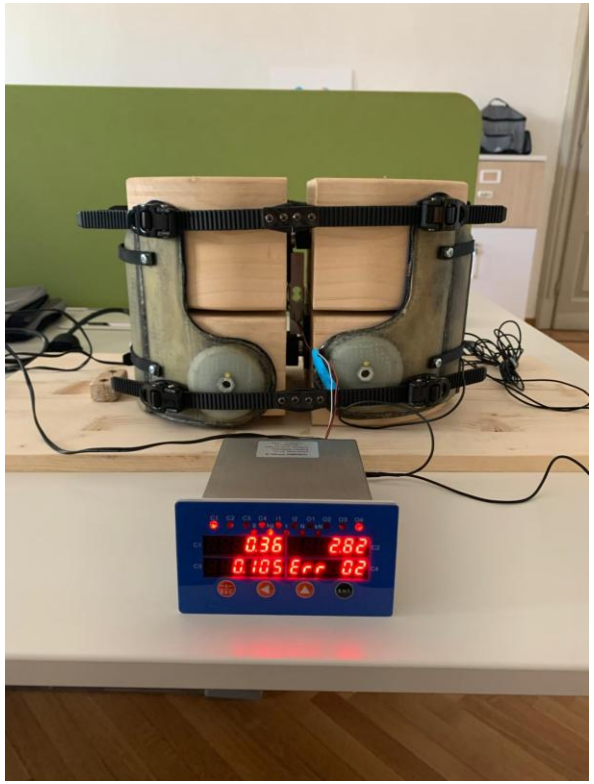

© MaCh3D s.r.l. Proprietary & Confidential

Material characterization of the torso

– *Characterization of the materials and mechanisms used in the construction of the torso, with the aim of evaluating the mechanical properties of these materials to be subsequently used for FEM analysis.*

© MaCh3D s.r.l. Proprietary & Confidential

Material and Mechanisms characterisation of the Torso – Neoprene

Neoprene is used for the inner part of the torso, onto which rigid components that structure the device are then attached.
Five samples with a rectangular geometry of 145 × 20 × 3 mm were prepared. The distance between the two grips (central section) is 45 mm.
It is relevant to note that, at the moment of clamping, the sample tends to lose tension, “bowing out.” This phenomenon is inevitable and is attributable to the way the clamping system operates. However, it is not problematic, as it can simply be taken into account during data processing.

Test parameters:
Speed: 20,000 µm/min
Sampling frequency: 10 Hz
Maximum displacement at the end of the test: 50,000 µm

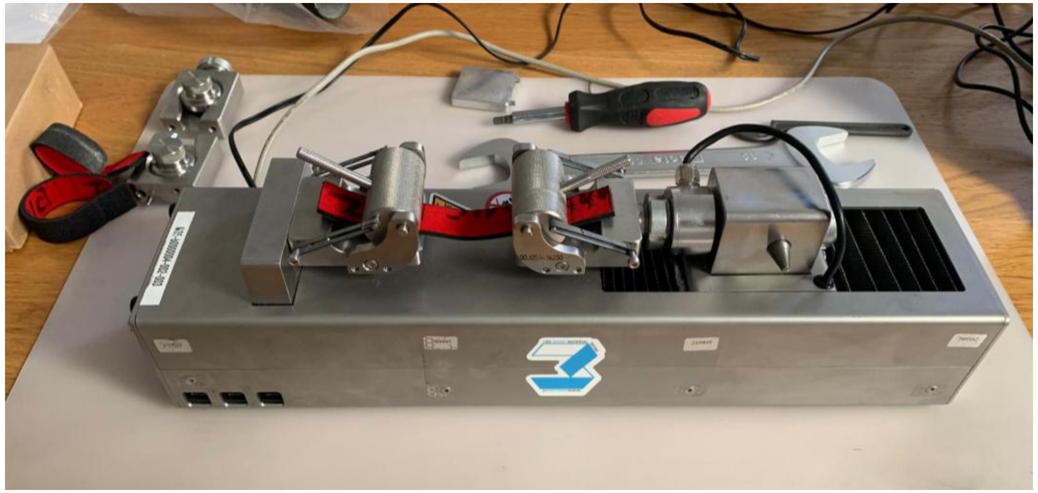

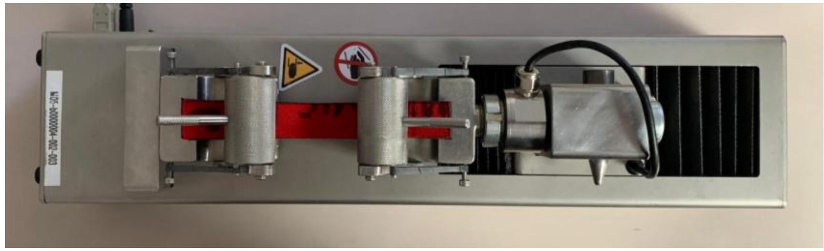

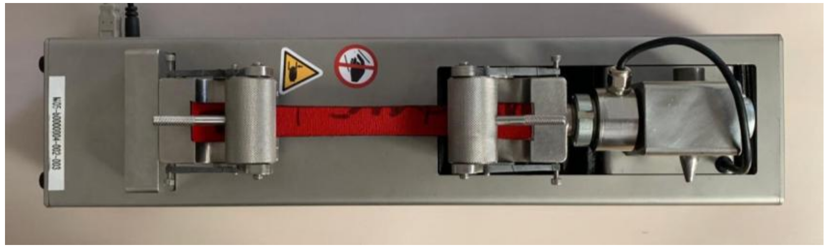

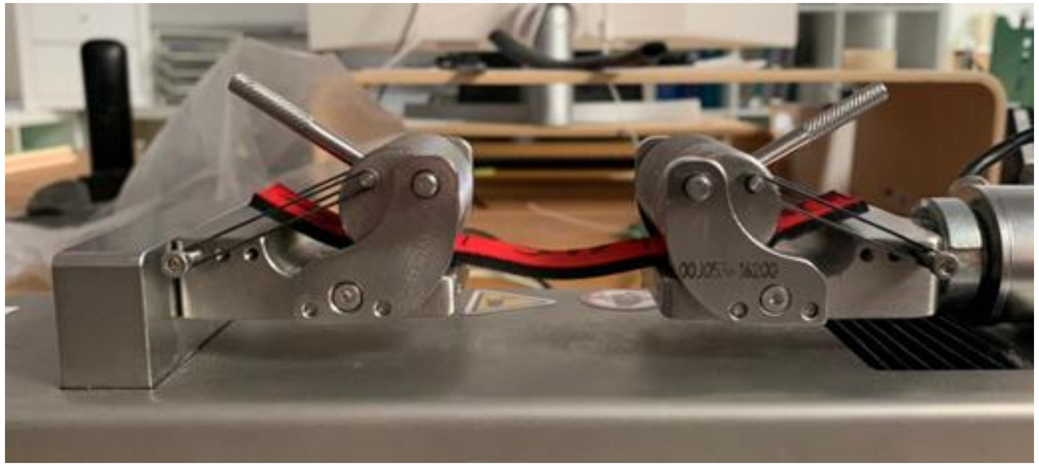

© MaCh3D s.r.l. Proprietary & Confidential

Material and Mechanisms characterisation of the Torso – Neoprene

Force–Displacement Curves

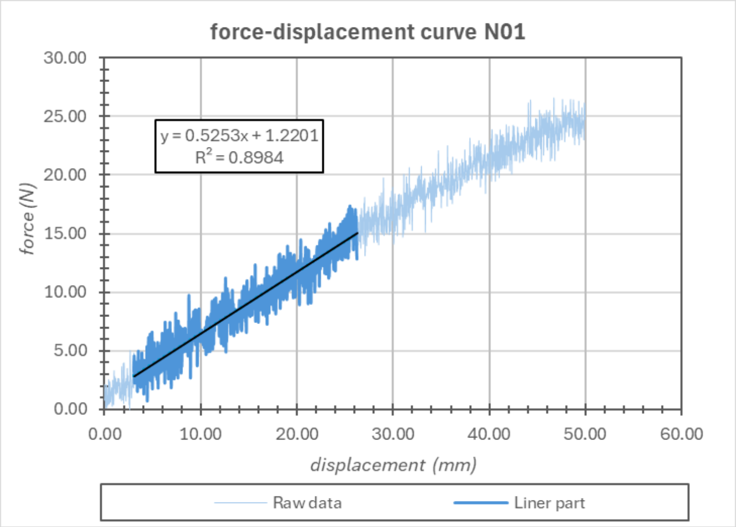

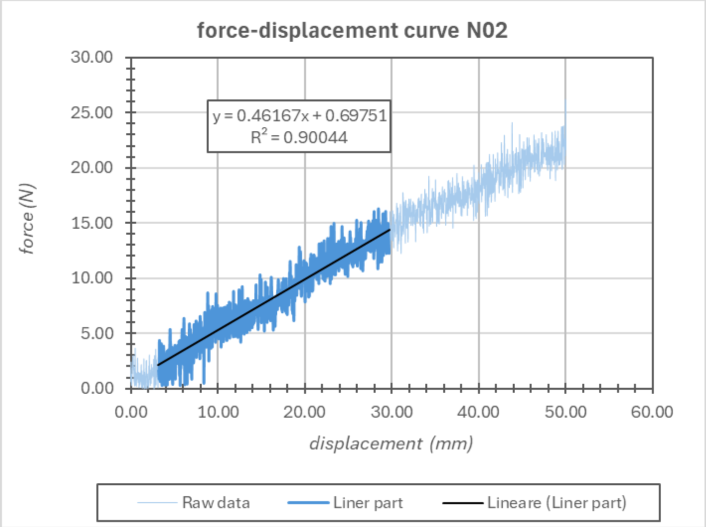

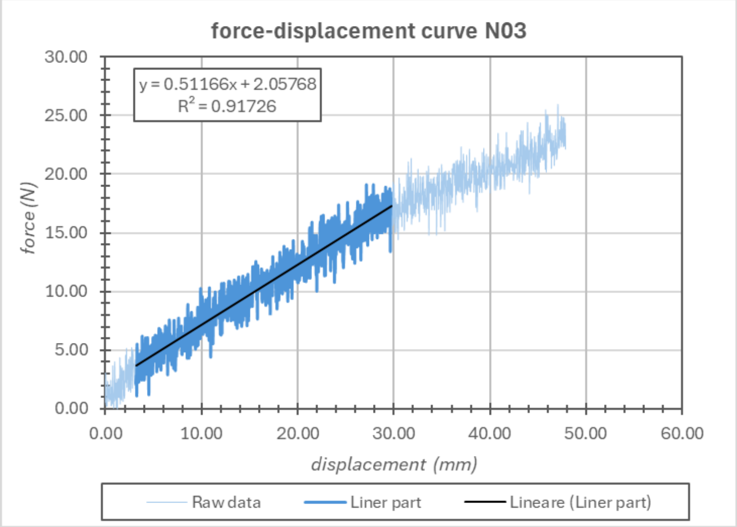

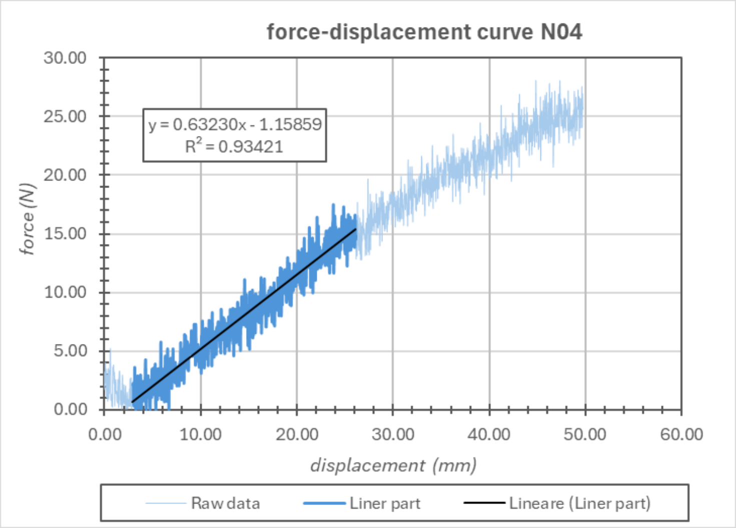

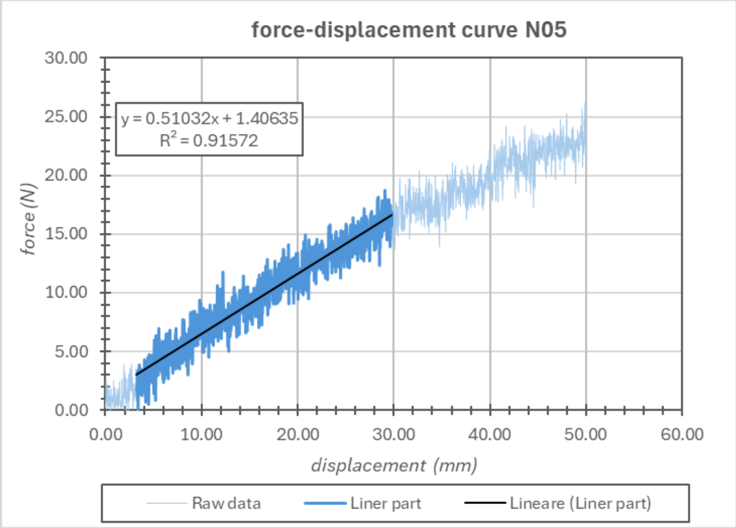

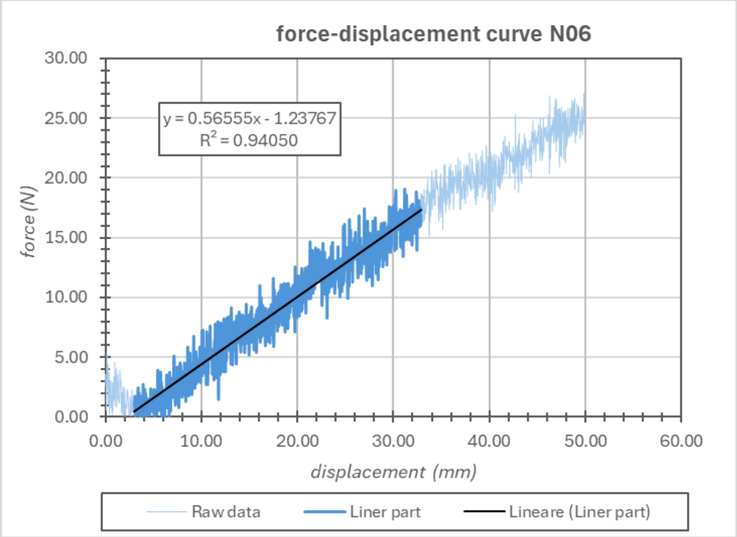

© MaCh3D s.r.l. Proprietary & Confidential

Material and Mechanisms characterisation of the Torso – Neoprene

Stress–Strain

From the tests, the stiffness (E) was determined.

Curves

Neoprene			
Stiffness	E	0.401	*MPa*
dev std		0.044	--

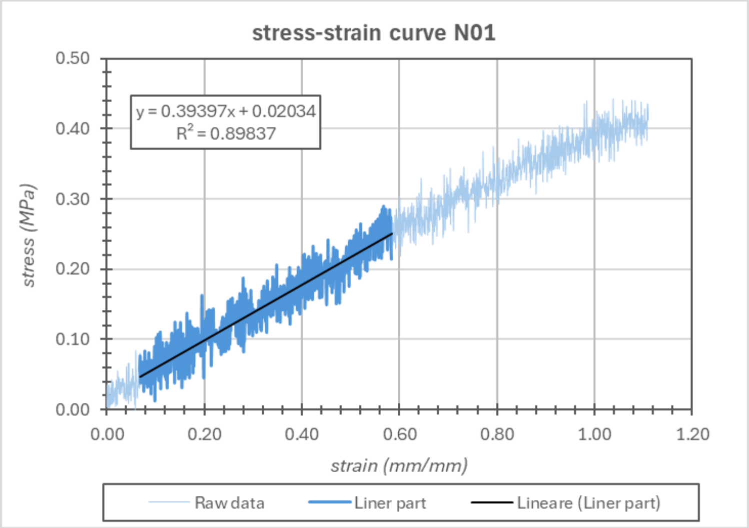

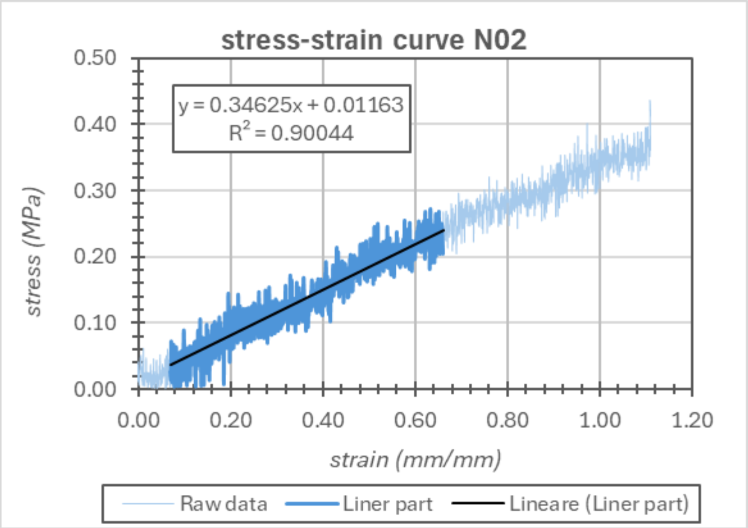

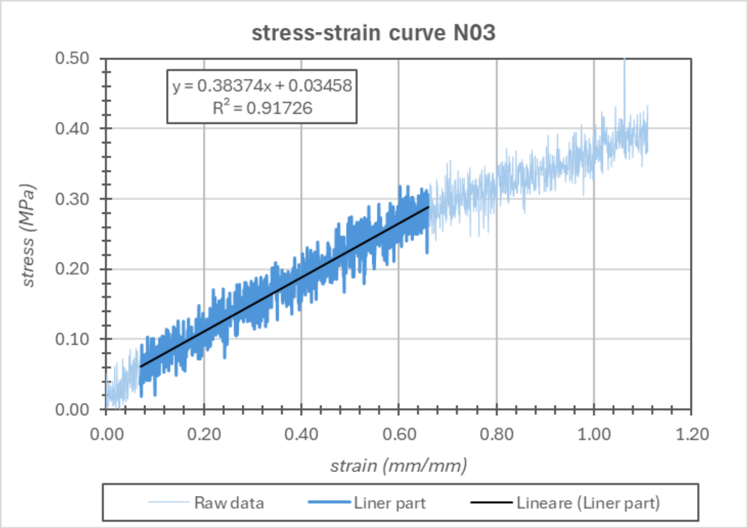

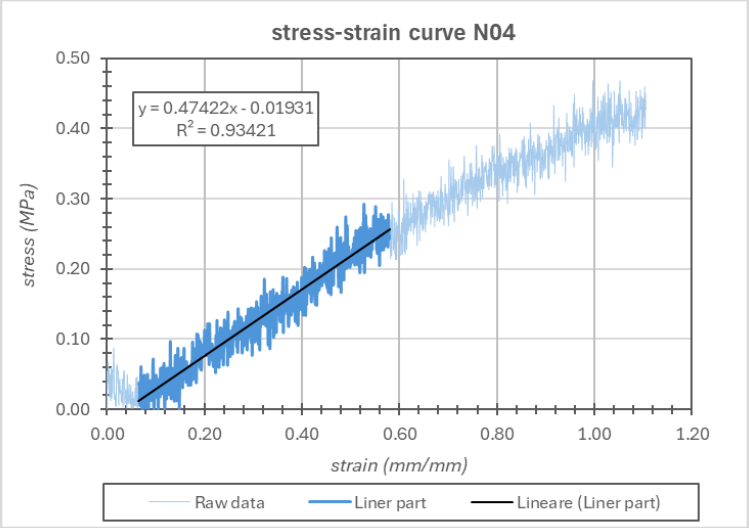

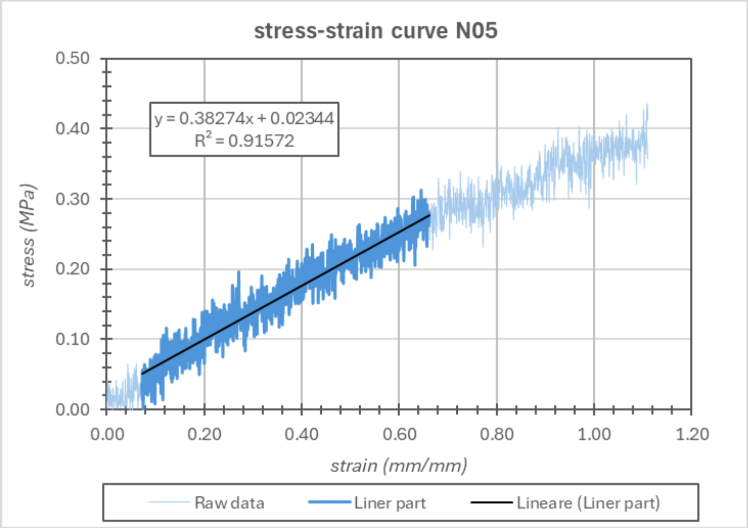

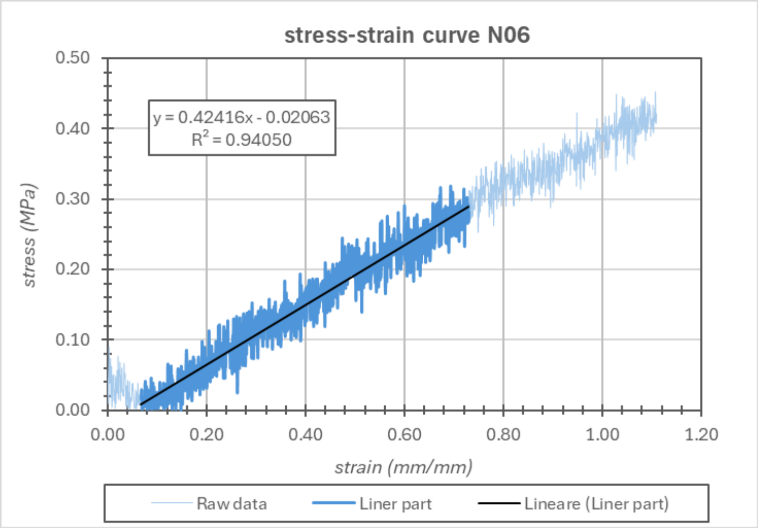

© MaCh3D s.r.l. Proprietary & Confidential

Material and Mechanisms characterisation of the Torso – Laces

Laces are used to connect the rigid parts of the torso to the neoprene. The function of the laces is to hold the rigid parts together, bearing the load to provide lateral support to the neoprene.
Two samples with a rectangular geometry of 92 × 10 × 1 mm were prepared. The distance between the two grips is 40 mm.
It is relevant, also in this case, that the sample tends to lose tension, "bowing out," at the moment of clamping.

Test parameters:
Speed: 5,000 μm/min
Sampling frequency: 10 Hz
Maximum displacement at the end of the test: 50,000 μm

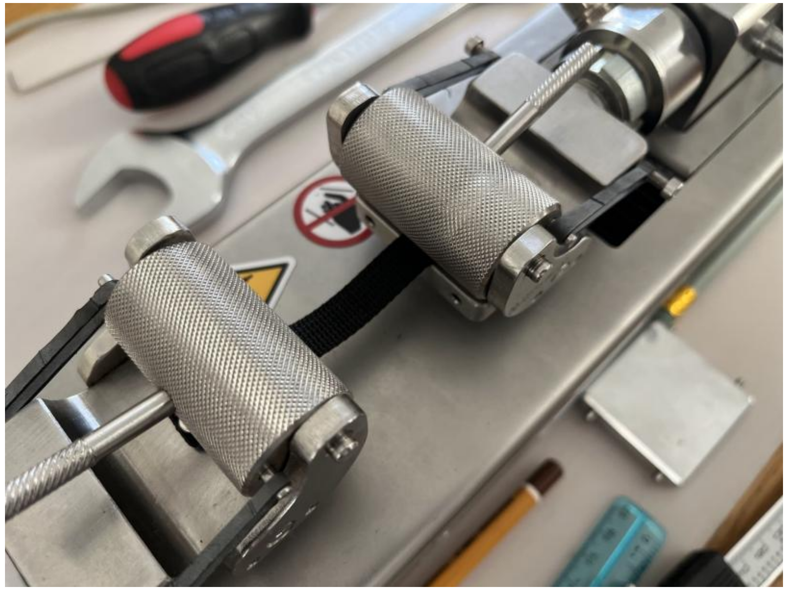

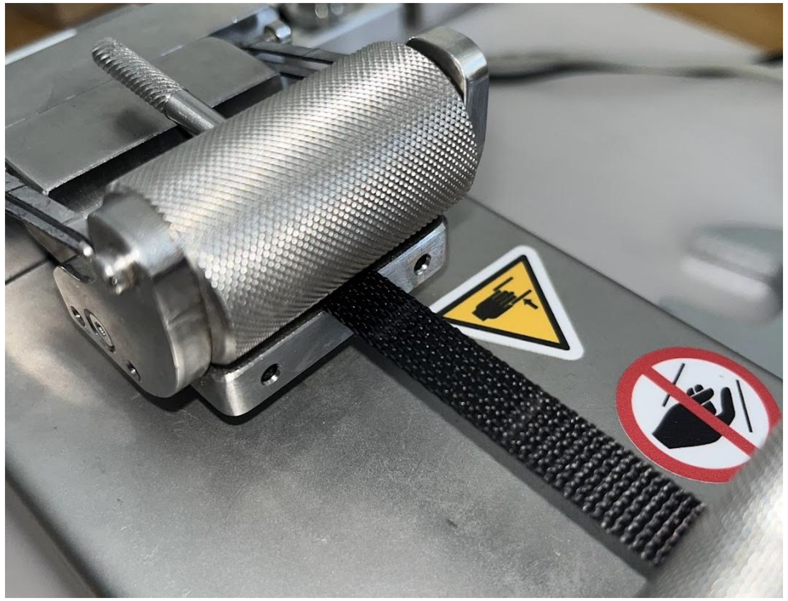

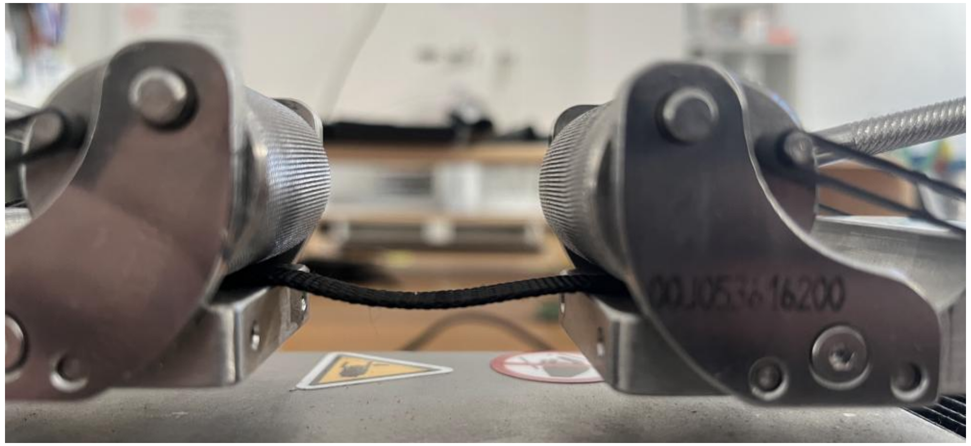

© MaCh3D s.r.l. Proprietary & Confidential

Material and Mechanisms characterisation of the Torso – Laces

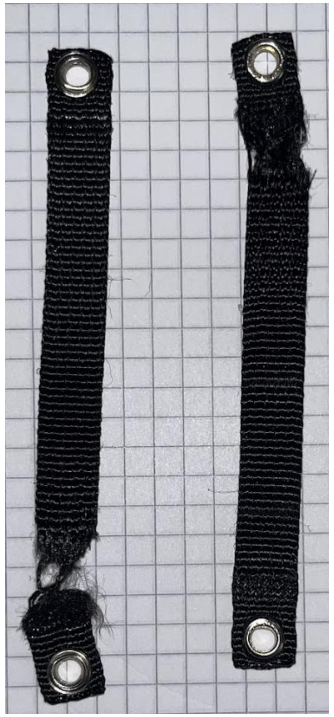

SEZIONE CAMPIONE			
Larghezza	B	10	*mm*
Altezza	H	1	*mm*
Lung. Iniziale	l_0	40	*mm*
Area sez.	A	10	*mm^2*

$$K = \frac{E * A}{l_0}$$

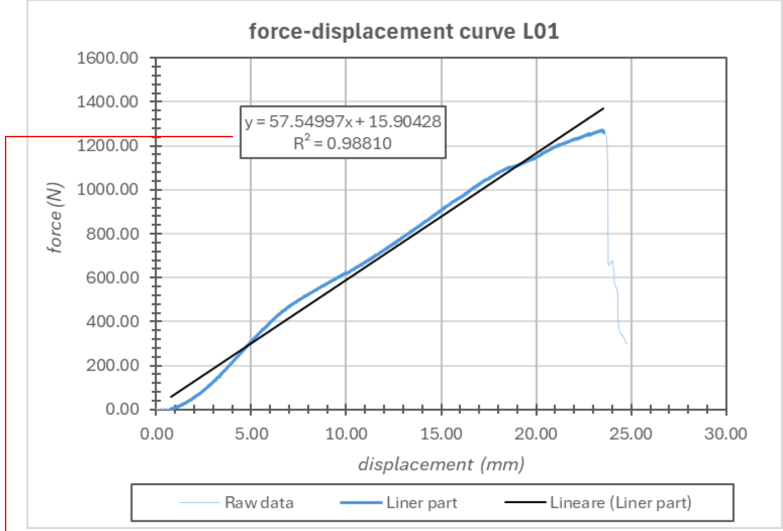

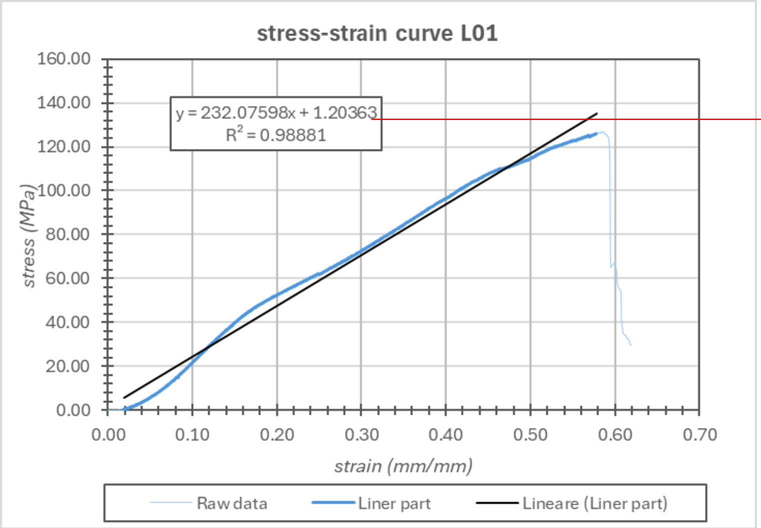

Modulo elastico E		
E	232.0760	*Mpa*
Costante elastica molla FEM		
K	58.019	*N/mm*

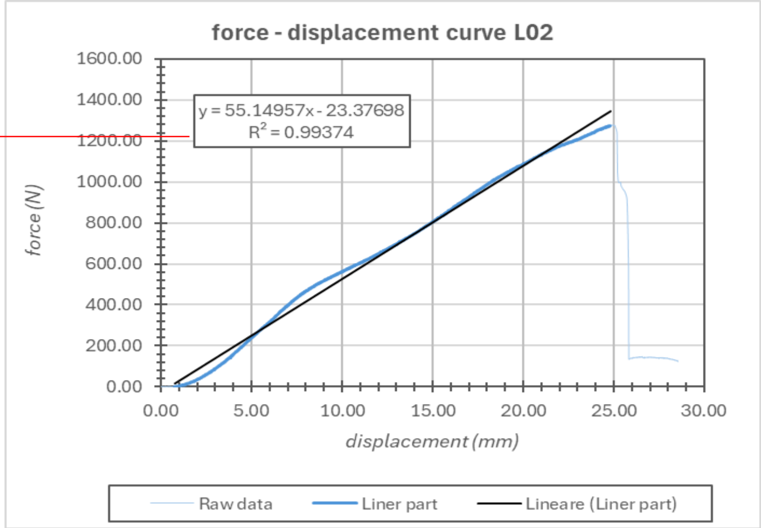

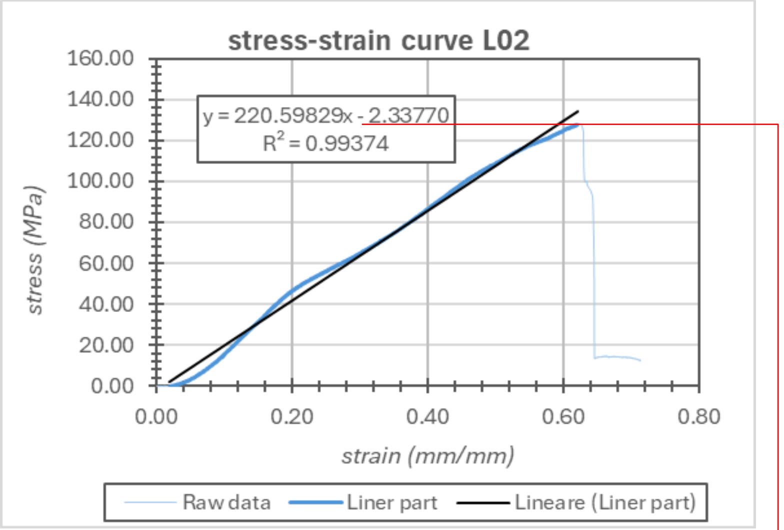

Modulo elastico E		
E	220.5983	*Mpa*
Costante elastica molla FEM		
K	55.150	*N/mm*

© MaCh3D s.r.l. Proprietary & Confidential

Material and Mechanisms characterisation of the Torso – Racks

Racks are used together with their corresponding latch for clamping the torso.
Two samples with a rectangular geometry of 80 × 14.5 mm were prepared. The distance between the two grips is 40 mm.
It is relevant that, at the moment of clamping, the sample tends to lose tension, “bowing out.”

Test parameters:
Speed: 5,000 μm/min
Sampling frequency: 10 Hz
Maximum displacement at the end of the test: 30,000 μm

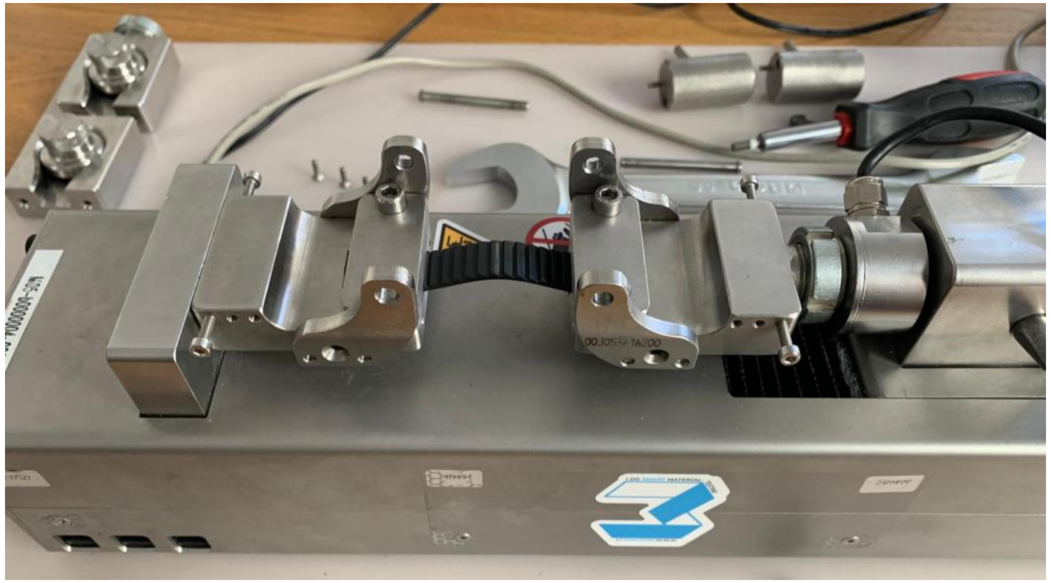

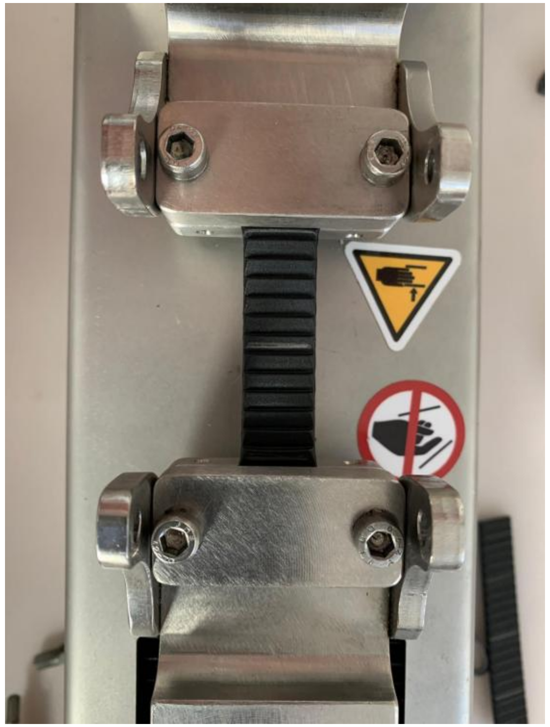

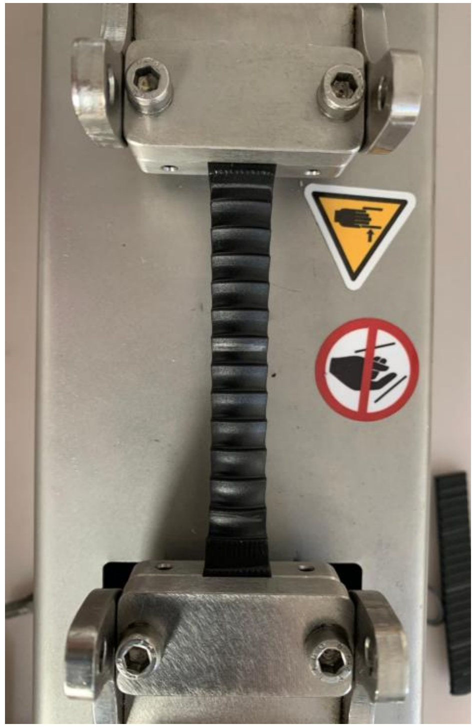

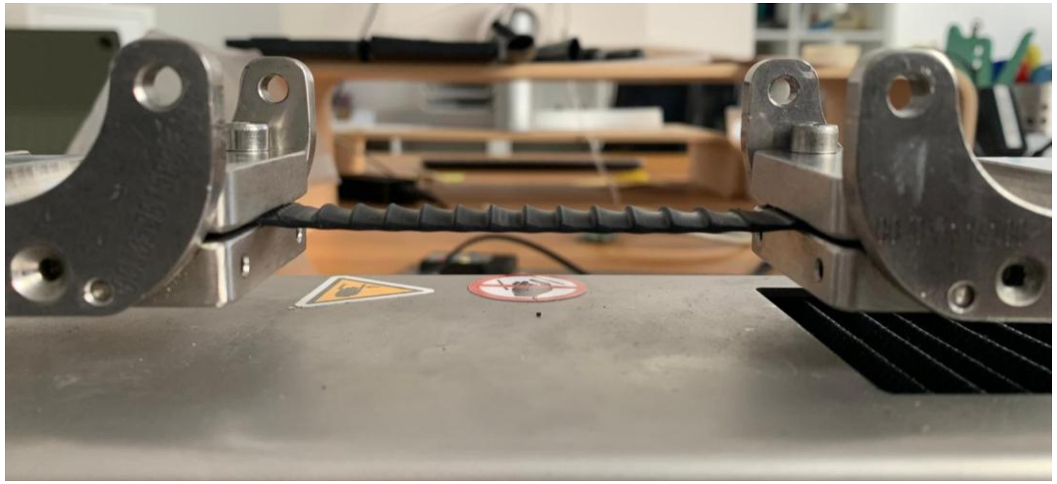

© MaCh3D s.r.l. Proprietary & Confidential

Material and Mechanisms characterisation of the Torso – Racks

The test was stopped by setting a maximum displacement of the moving crosshead at 50 mm.

The useful operating range is approximately 370 N, which corresponds to the maximum load at which the latch slips on the rack itself.

For the characterization of the rack, the average cross-section was used, since it is made up of ridges and the cross-section therefore varies between a maximum and a minimum value.

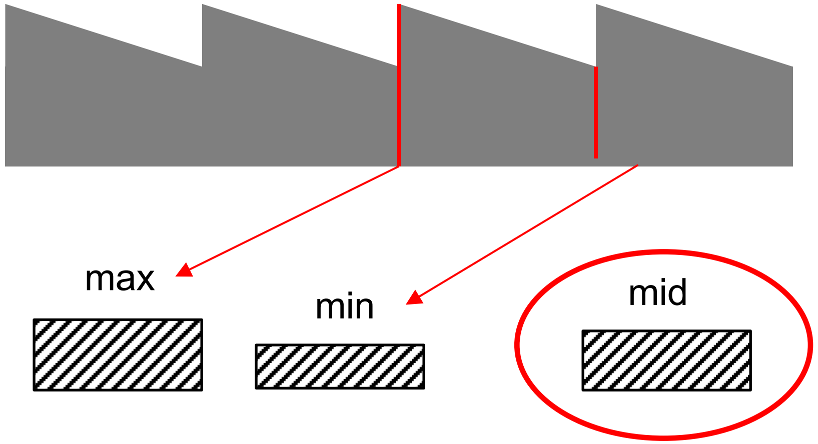

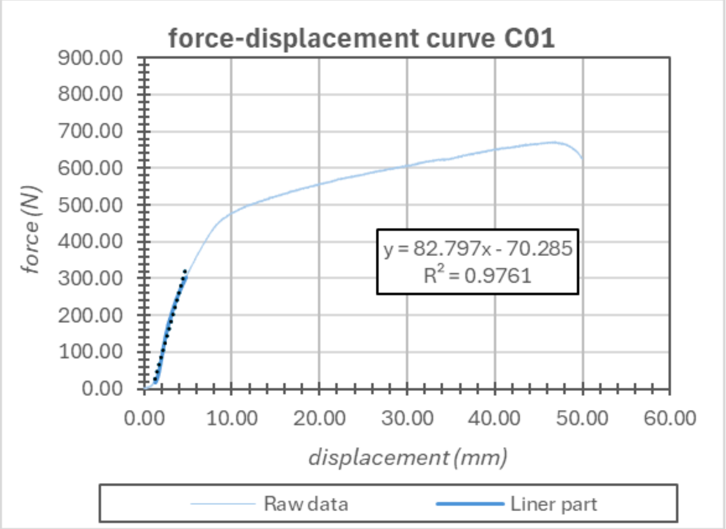

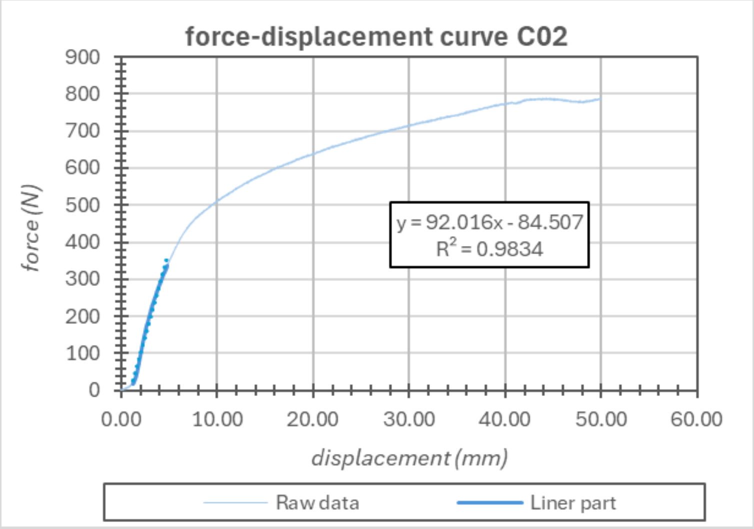

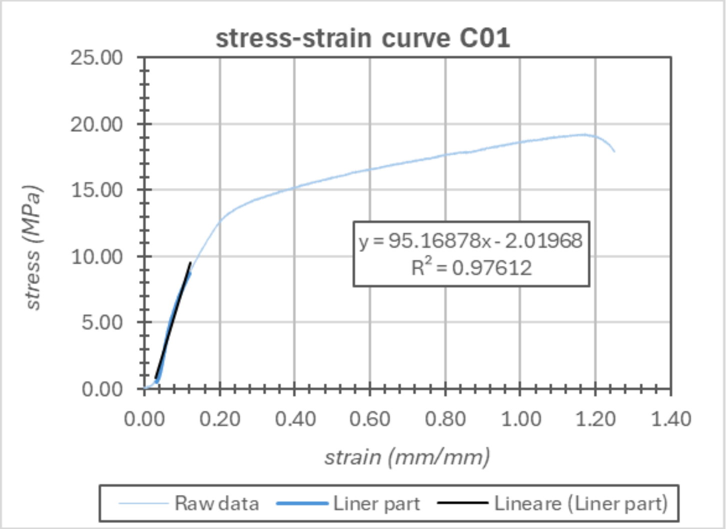

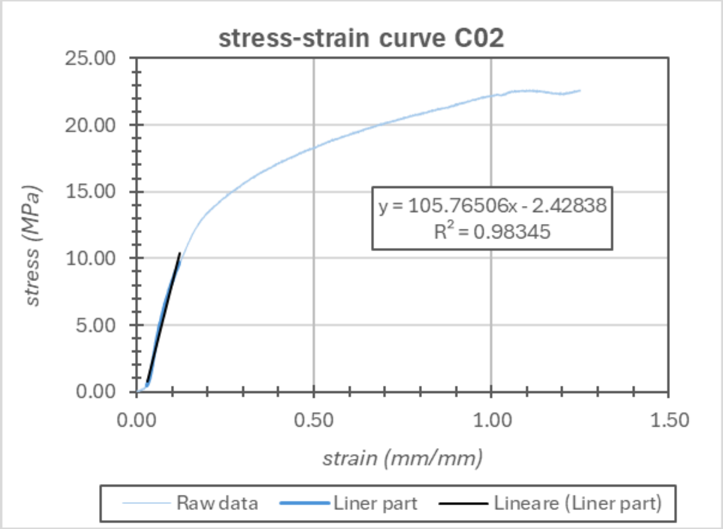

© MaCh3D s.r.l. Proprietary & Confidential

Material and Mechanisms characterisation of the Torso – Latch

Latch which, together with the rack, forms the torso closure system.
Rack dimensions: 120 × 14.5 × 1.8 mm.
A 3D-printed adapter was used to attach the torso latch to the standard MaCh grip.

Test parameters:
Speed: 5,000 μm/min
Sampling frequency: 10 Hz
Maximum displacement at the end of the test: 30,000 μm

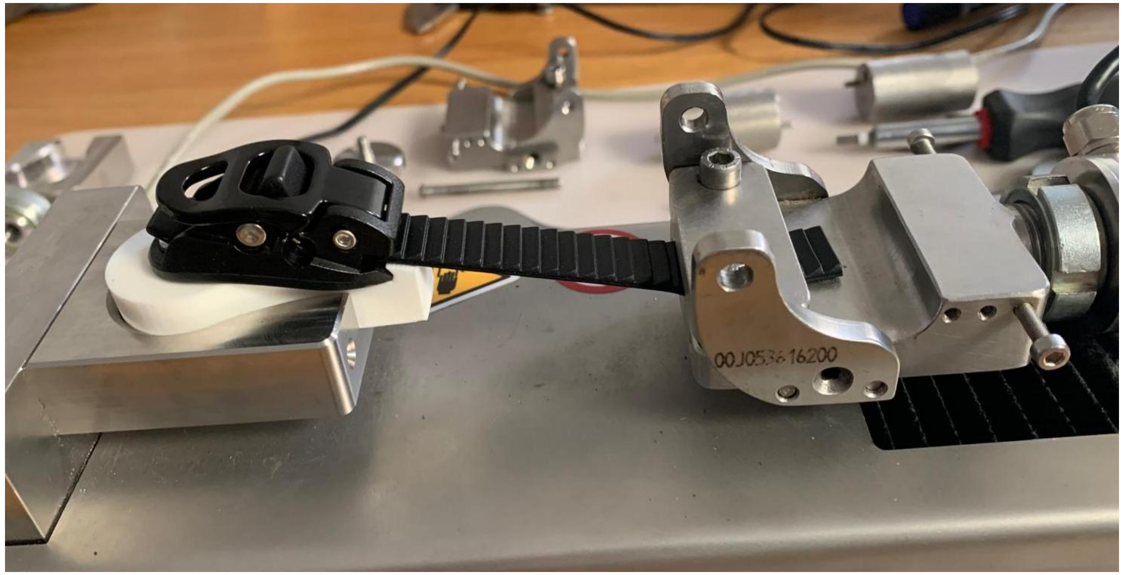

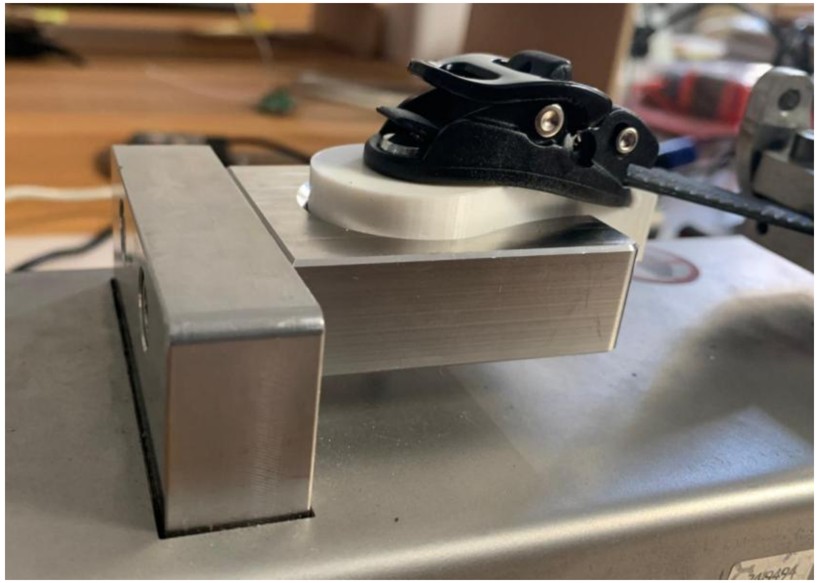

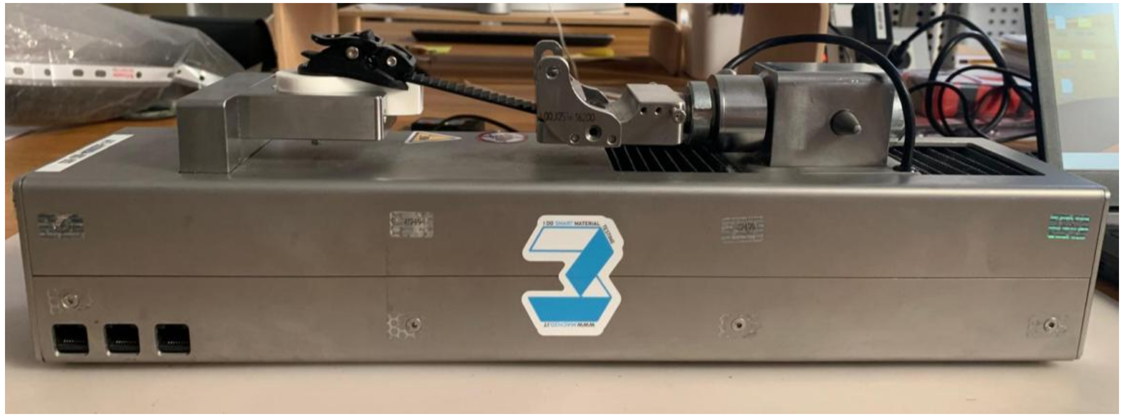

© MaCh3D s.r.l. Proprietary & Confidential

Material and Mechanisms characterisation of the Torso – Latch

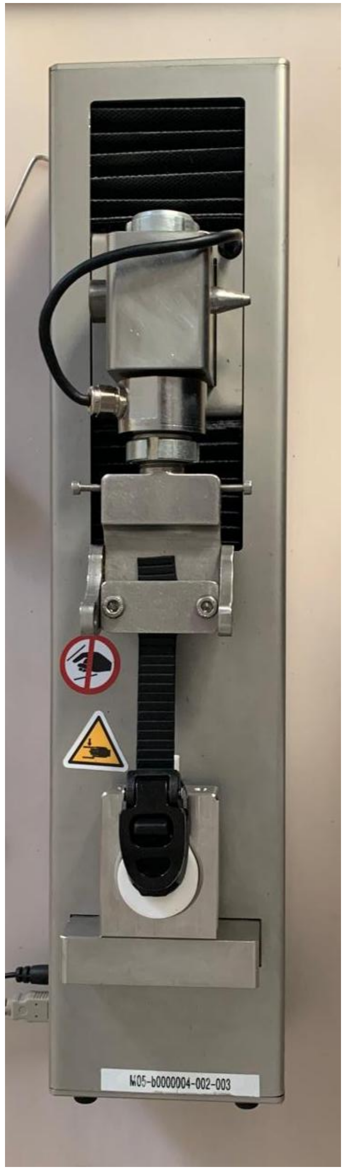

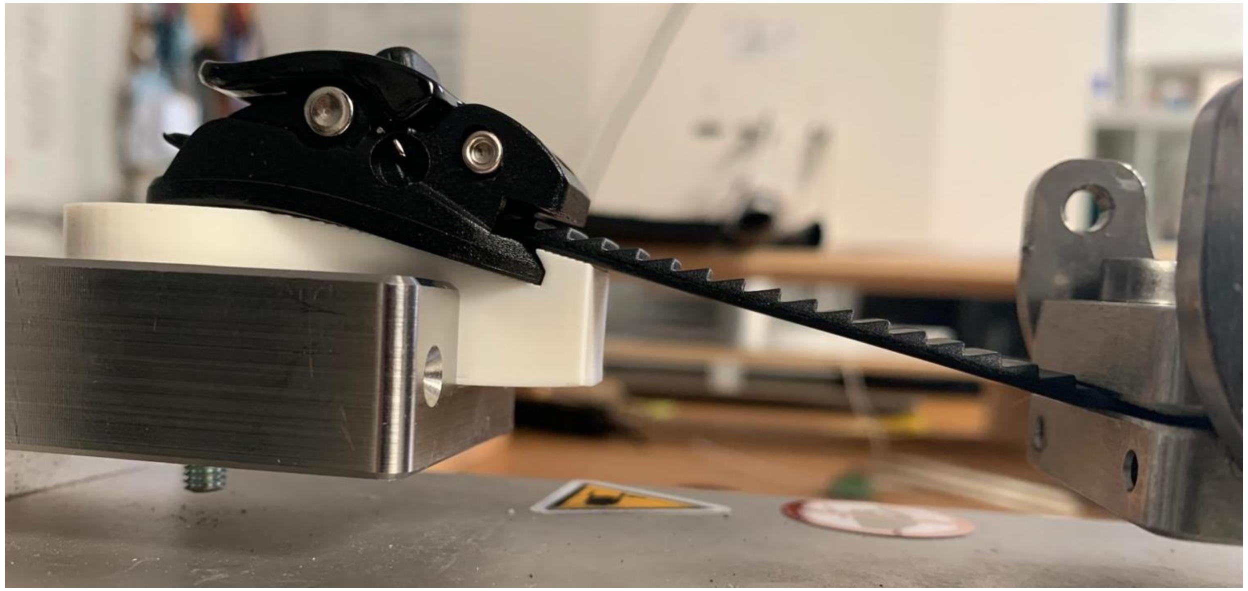

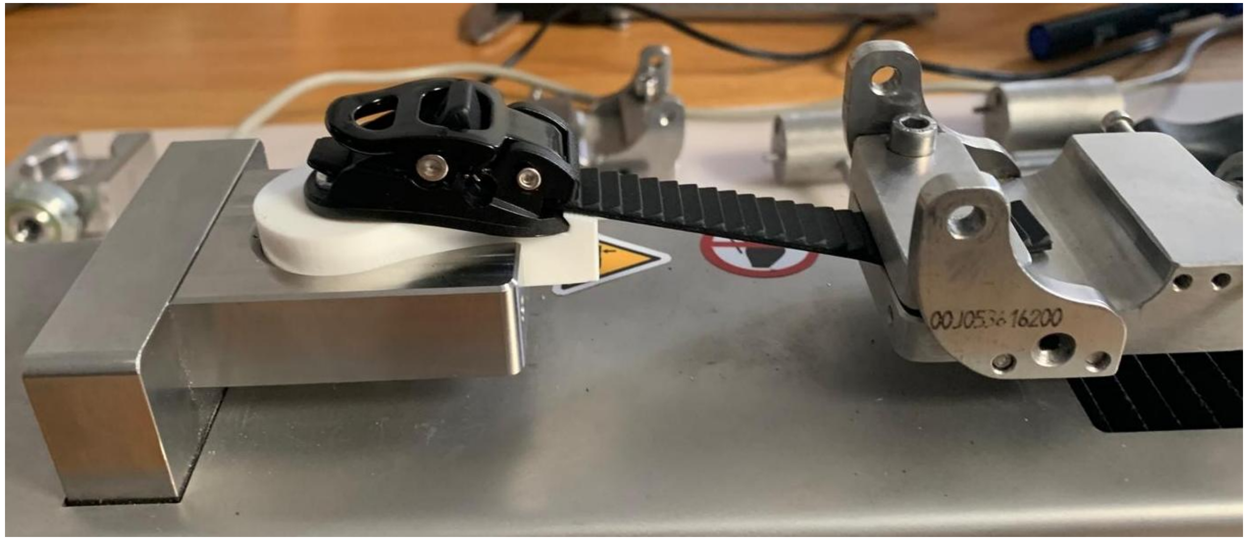

© MaCh3D s.r.l. Proprietary & Confidential

Material and Mechanisms characterisation of the Torso – Latch

For the tests, a single rack and a single latch were used.

Test procedure:
Initial setup with the "tooth engaged."
First pull.
Upon trigger point, the test was stopped and the latch returned to the same "tooth engaged."
Subsequent pulls.

Observations:
The trigger load decreases over the first, second, and third pulls, then stabilizes.
Fmax_1 (blue) = 373 N
Fmax_2 (green) = 343 N
Fmax_3–5 = 300 N (stabilized)

This decrease is attributable to the deformation of the engaged tooth of the rack with the latch, which progressively compromised the engagement efficiency during the tests.
This behavior realistically simulates the effect of wear on the engaged tooth on the force required to release the latch.

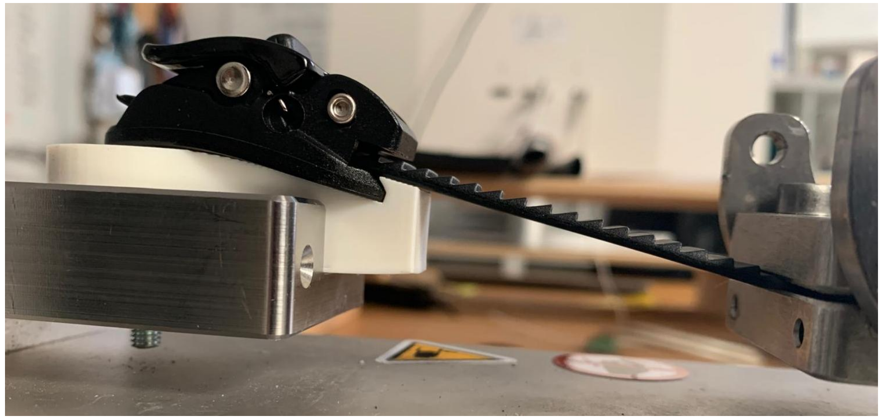

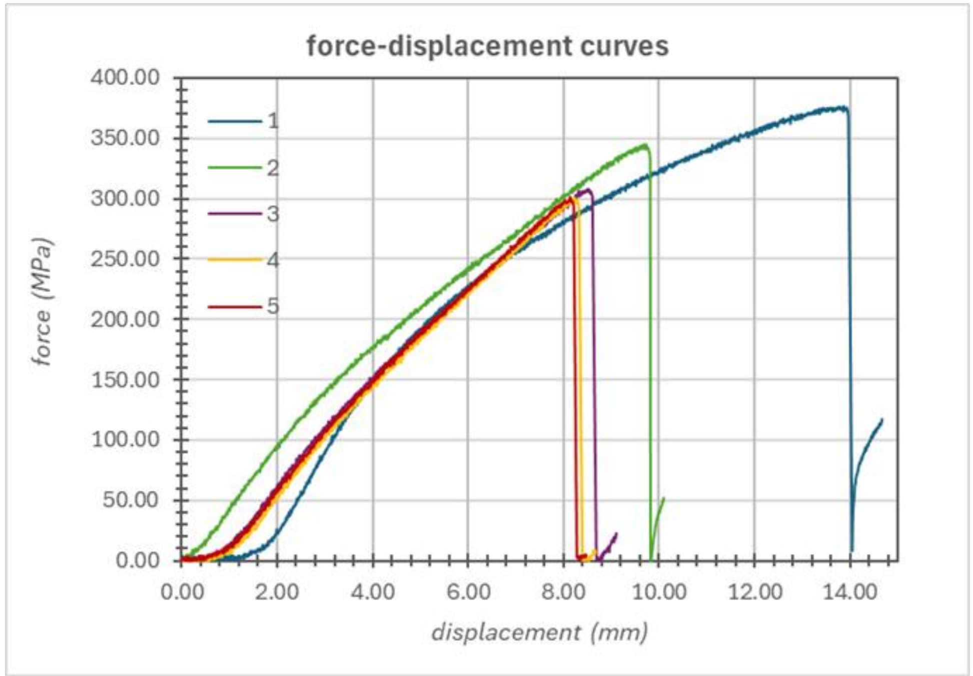

© MaCh3D s.r.l. Proprietary & Confidential

Experimental Setup and Tests Conducted

- *Zeroing procedure and types of tests conducted.*

© MaCh3D s.r.l. Proprietary & Confidential

Experimental Test Procedure

- Each test started from a zeroing condition, in which the goal was to obtain a load cell reading lower than 1 kg, while ensuring that the value was repeatable at the beginning of every test.
- To reach this condition, a defined series of clicks was applied to the upper and lower parts of the torso until the load-cell readings were greater than 0 and less than 1 kg. The sequence used was: 2D 2D 2S 2S – 1S 1D – (2D 1S 1D 2S). The sequence before the parentheses is fixed, while the sequence within the parentheses is the reverse of the loading sequence. In general, the readings from both cells occurred during one of the clicks within the bracketed sequence. If one load cell is reading and the other is not, clicks are applied to the latch of the cell that is not reading.
- Once the zeroing condition was achieved, the clicks were applied following the predefined sequence: **2S – 1D – 1S – 2D.**

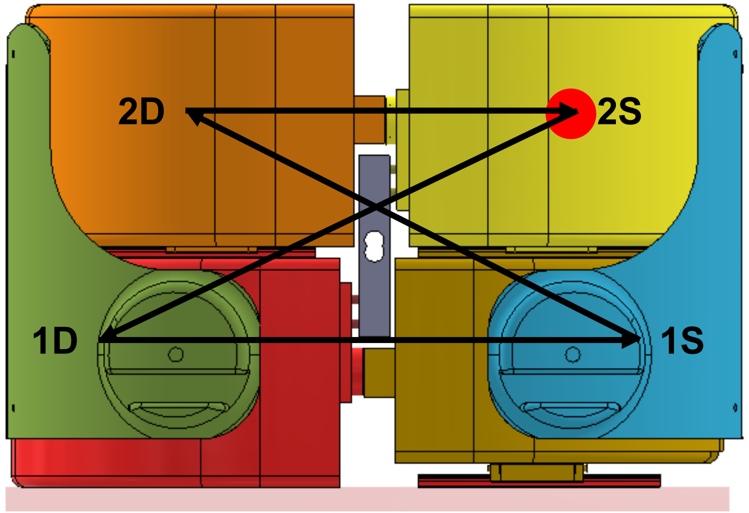

© MaCh3D s.r.l. Proprietary & Confidential

Experimental Test Conducted

- Three different types of Load Cells tested: CTC20, CCC20 and CCC50.
- Three different types of laces tested: LS (Single), LD (Double), LBD (Double bonded) and NL(Whithout laces).
- Two different types of misalignment:D2 (2 mm), D4 (4 mm) and D0 (Aligned). The spacers were applied on 1D and 2S.
- Test conducted:

CTC20	D0	D2	D4
LS			
LD	v	v	v
LDB			
NL	v		

*CTC20 Screw

CCC20	D0	D2	D4
LS	v		
LD	v	v	v
LDB	v		
NL	v		

CCC50	D0	D2	D4
LS	v		
LD	v	v	v
LDB	v	v	v
NL	v		

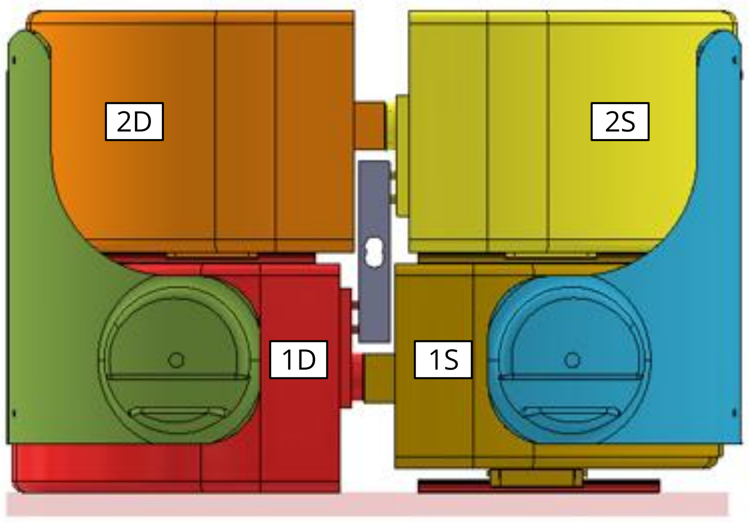

CTC

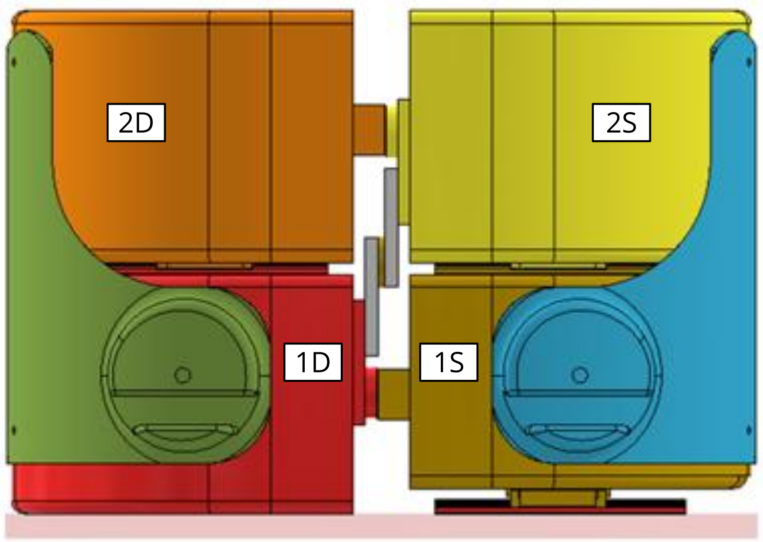

CCC

© MaCh3D s.r.l. Proprietary & Confidential

Experimental Result – 20 Kg Load Cells (CTC)

- *Experimental results of the tests conducted on the torso.*

© MaCh3D s.r.l. Proprietary & Confidential

Test without spacers - CTC20-D0-LD

- Three tests were carried out using the same reference setup, without the use of spacers.
  This configuration makes it possible to evaluate the magnitude of the force that the brace can apply to the torso under nominal alignment conditions.
- From the analysis of the graphs, it can be observed that the shear load cell records lower force values compared to the button load cells. This result can be attributed to the absence of misalignments between the torso sections, confirming the correct functioning of the developed device.
- The forces measured by the two button load cells show an oscillating trend, resulting from the sequential execution of the clicks, one at a time.

LD		Load cell		
		Bottom	Top	Mid
Step	Buckle Id	C1	C2	C3
--	--	*N*	*N*	*N*
0	--	7.06	4.12	2.06
1	2S	6.67	14.71	3.58
2	1D	17.85	13.73	4.41
3	1S	37.27	14.91	5.00
4	2D	34.91	37.07	3.78
5	2S	31.38	78.65	5.30
6	1D	61.19	77.47	7.70
7	1S	96.11	80.81	8.58
8	2D	88.46	134.35	6.52
9	1D	141.22	128.47	11.87

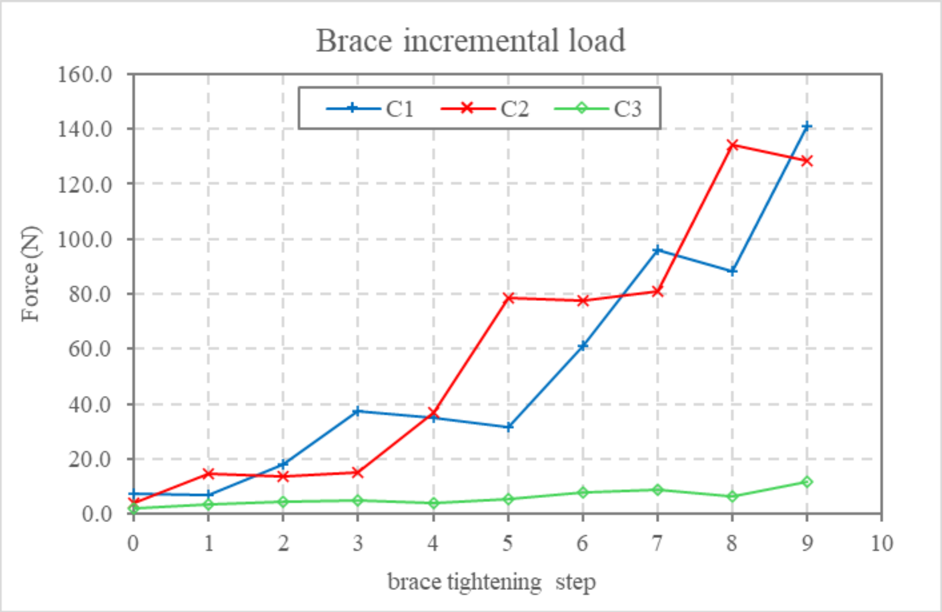

© MaCh3D s.r.l. Proprietary & Confidential

Test without spacers - CTC20-D0-LD

LD		Load cell		
		Bottom	Top	Mid
Step	Buckle Id	C1	C2	C3
--	--	*N*	*N*	*N*
0	--	8.83	2.35	0.00
1	2S	9.41	11.96	0.00
2	1D	17.46	10.98	2.11
3	1S	36.28	13.34	0.00
4	2D	34.13	35.11	0.00
5	2S	30.99	74.53	1.91
6	1D	58.84	73.55	3.78
7	1S	96.89	79.24	2.99
8	2D	88.65	133.37	0.00
9	1D	138.08	129.64	5.25

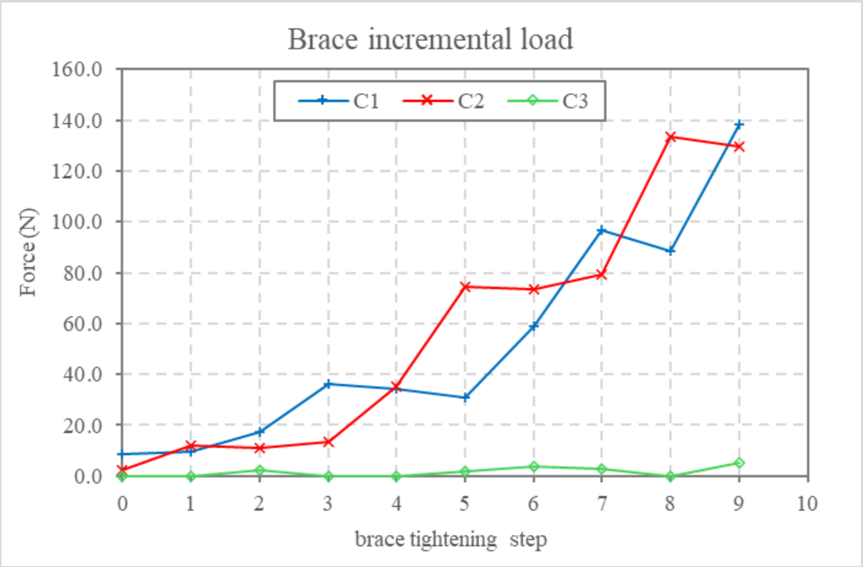

LD		Load cell		
		Bottom	Top	Mid
Step	Buckle Id	C1	C2	C3
--	--	*N*	*N*	*N*
0	--	5.49	0.00	2.30
1	2S	5.49	8.43	3.19
2	1D	14.71	8.04	5.49
3	1S	33.54	9.41	6.13
4	2D	31.77	31.19	4.81
5	2S	27.85	71.59	6.67
6	1D	54.92	69.63	9.32
7	1S	95.91	72.57	11.57
8	2D	87.87	131.80	8.14
9	1D	140.43	128.27	14.56

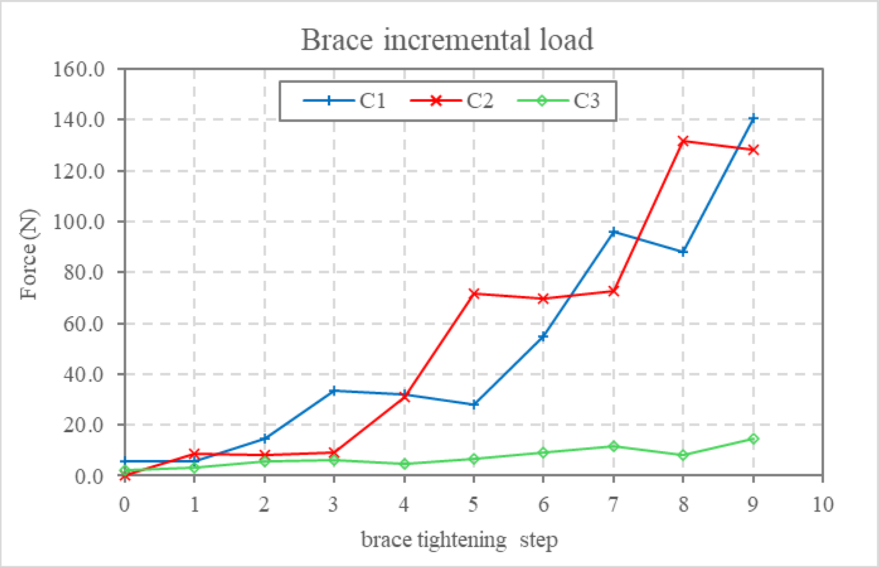

Test without Spacers and Straps – CTC20-D0-NL

© MaCh3D s.r.l. Proprietary & Confidential

- Test was carried out with the brace straps removed in order to assess their influence on the forces measured by the button load cells.
- The results show that the maximum force levels remain unchanged; however, as expected, in the absence of the straps a higher number of clicks is required due to the increased deformation of the neoprene.

NL		Load cell	
		Bottom	Top
Step	Buckle Id	C1	C2
--	--	*N*	*N*
0	--	0.00	4.51
1	1D	2.35	3.14
2	1S	10.40	4.51
3	2D	10.00	12.55
4	2S	11.38	20.20
5	1D	22.16	21.77
6	1S	39.81	23.93
7	2D	39.62	41.58
8	1D	37.07	59.04
9	1S	58.64	62.76
10	2D	71.20	63.15
11	2S	68.06	84.14
12	1D	67.67	100.62
13	1S	102.97	109.05
14	2D	125.13	111.01
15	1D	111.80	118.07
16	1S	114.35	140.04
17	2D	143.18	141.02

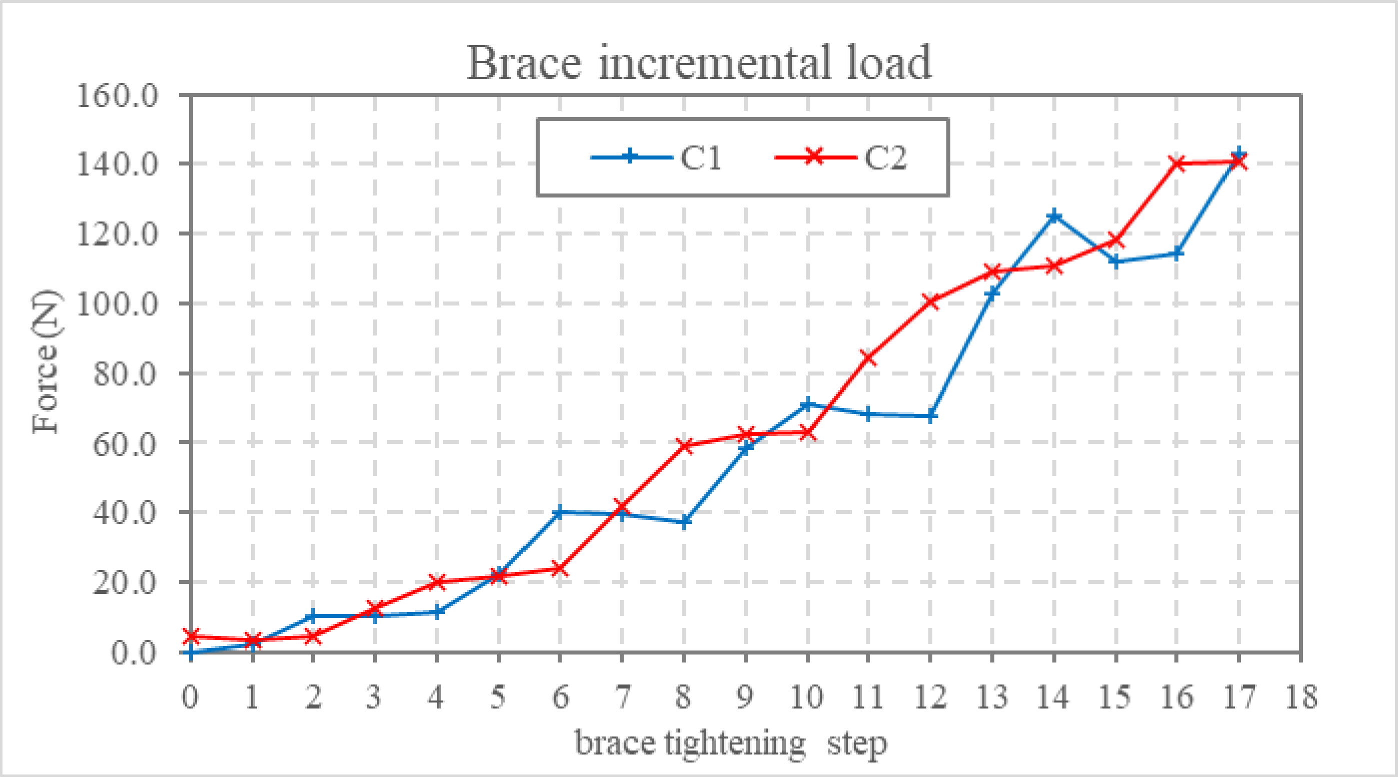

© MaCh3D s.r.l. Proprietary & Confidential

Tests with 2 mm Spacers – CTC20-D2-LD

- Three tests were carried out using 3D-printed spacers with a thickness of 2 mm.
- This setup allows for the evaluation of the shear force that the brace can apply to the torso in the presence of a controlled and limited misalignment.
- By comparing the results with the previous diagrams, it becomes evident that, in this case, the shear force assumes a significantly higher value, confirming the influence of misalignment on the distribution of forces among the load cells.

LD		Load cell		
		Bottom	Top	Mid
Step	Buckle Id	C1	C2	C3
--	--	*N*	*N*	*N*
0	--	0.00	0.00	9.17
1	2S	0.00	0.00	14.27
2	1D	6.28	0.00	18.93
3	1S	18.83	0.00	21.87
4	2D	19.61	3.53	25.50
5	2S	15.10	18.04	32.75
6	1D	33.54	15.10	43.05
7	1S	61.00	14.51	51.93
8	2D	56.88	39.81	54.52
9	2S	50.21	79.63	65.70
10	1D	92.38	76.10	79.14
11	1S	132.39	76.69	88.65
12	2D	127.68	131.61	93.16

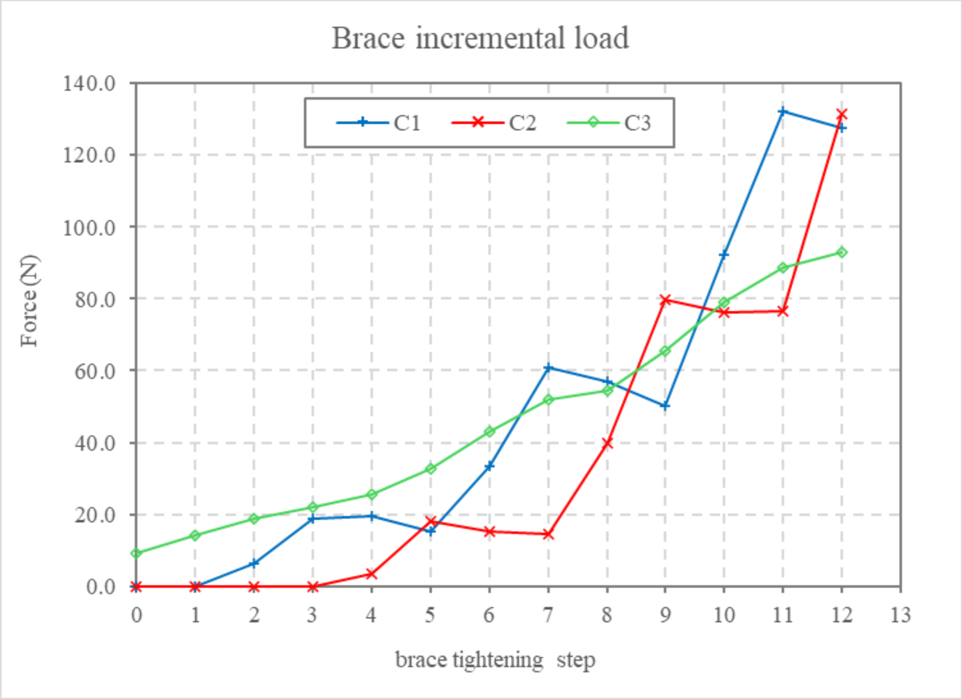

Tests with 2 mm Spacers – CTC20-D2-LD

LD		Load cell		
		Bottom	Top	Mid
Step	Buckle Id	C1	C2	C3
--	--	*N*	*N*	*N*
0	--	5.30	0.00	5.93
1	2S	3.92	0.00	10.30
2	1D	9.81	0.00	14.46
3	1S	20.40	0.00	19.22
4	2D	20.59	3.92	22.36
5	2S	17.65	19.61	28.24
6	1D	35.89	16.48	36.53
7	1S	61.78	15.10	45.55
8	2D	58.84	41.38	49.18
9	2S	52.96	83.16	58.45
10	1D	94.93	76.49	72.57
11	1S	139.84	75.32	84.63
12	2D	132.59	131.61	88.90

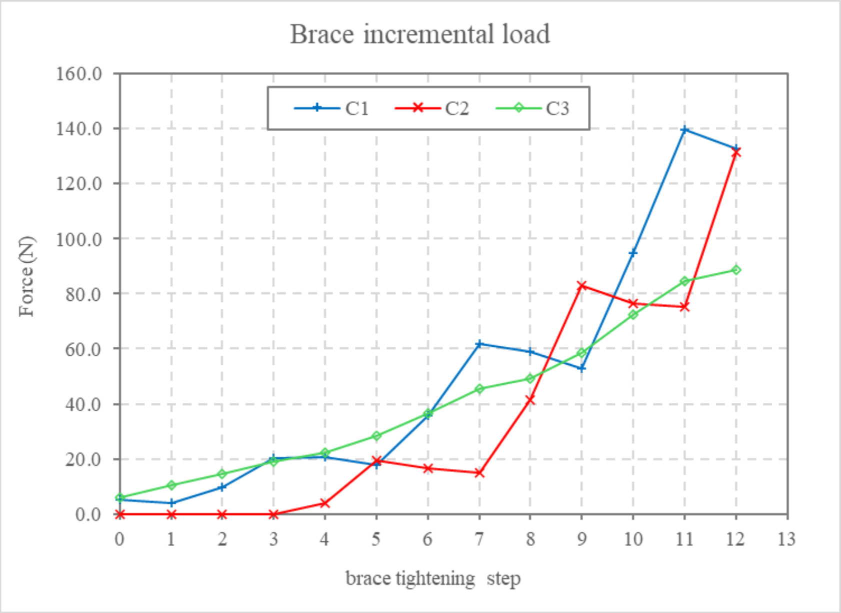

LD		Load cell		
		Bottom	Top	Mid
Step	Buckle Id	C1	C2	C3
--	--	*N*	*N*	*N*
0	--	2.75	0.00	4.90
1	2S	3.92	0.00	5.20
2	1D	10.00	0.00	10.59
3	1S	19.81	0.00	15.40
4	2D	19.61	3.14	19.32
5	2S	16.48	16.67	24.37
6	1D	35.50	13.14	35.75
7	1S	61.10	12.94	44.23
8	2D	59.04	39.62	46.68
9	2S	51.39	79.63	56.39
10	1D	94.14	74.14	73.75
11	1S	140.82	74.73	84.68
12	2D	131.02	134.55	83.36

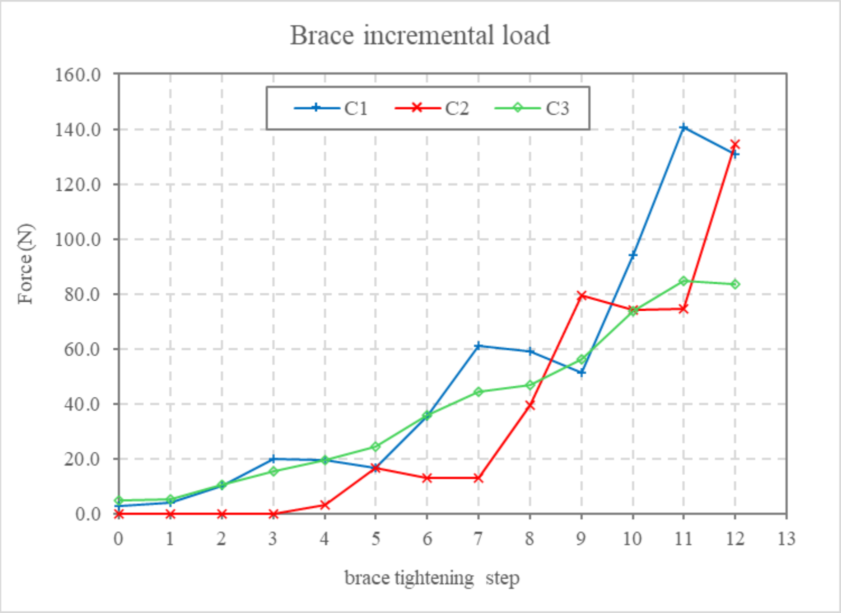

© MaCh3D s.r.l. Proprietary & Confidential

© MaCh3D s.r.l. Proprietary & Confidential

Tests with 4 mm Spacers – CTC20-D4-LD

- Three tests were carried out using the same setup, employing 3D-printed spacers with a thickness of 4 mm.
- This configuration allows for the evaluation of the shear force that the brace can apply to the torso under conditions of pronounced misalignment.
- From the analysis of the diagrams, it can be observed that during the first five or six clicks, the button load cells do not register any significant reading, whereas the shear load cell records consistently high force values. This behavior confirms the predominance of the shear contribution (corrective force) in the presence of a marked misalignment between the torso sections.

LD		Load cell		
		Bottom	Top	Mid
Step	Buckle Id	C1	C2	C3
--	--	*N*	*N*	*N*
0	--	0.00	0.00	12.26
1	2S	0.00	0.00	16.18
2	1D	0.00	0.00	24.12
3	1S	0.00	0.00	30.50
4	2D	0.00	0.00	36.38
5	2S	0.00	5.10	42.61
6	1D	16.08	0.00	55.21
7	1S	37.27	0.00	65.70
8	2D	34.91	8.24	73.11
9	2S	34.32	27.26	80.71
10	1D	68.74	20.40	99.73
11	1S	106.30	26.48	111.50
12	2D	103.95	64.33	120.33
13	2S	101.60	101.20	129.15
14	1D	145.92	95.71	147.10
15	2D	137.88	153.77	148.08

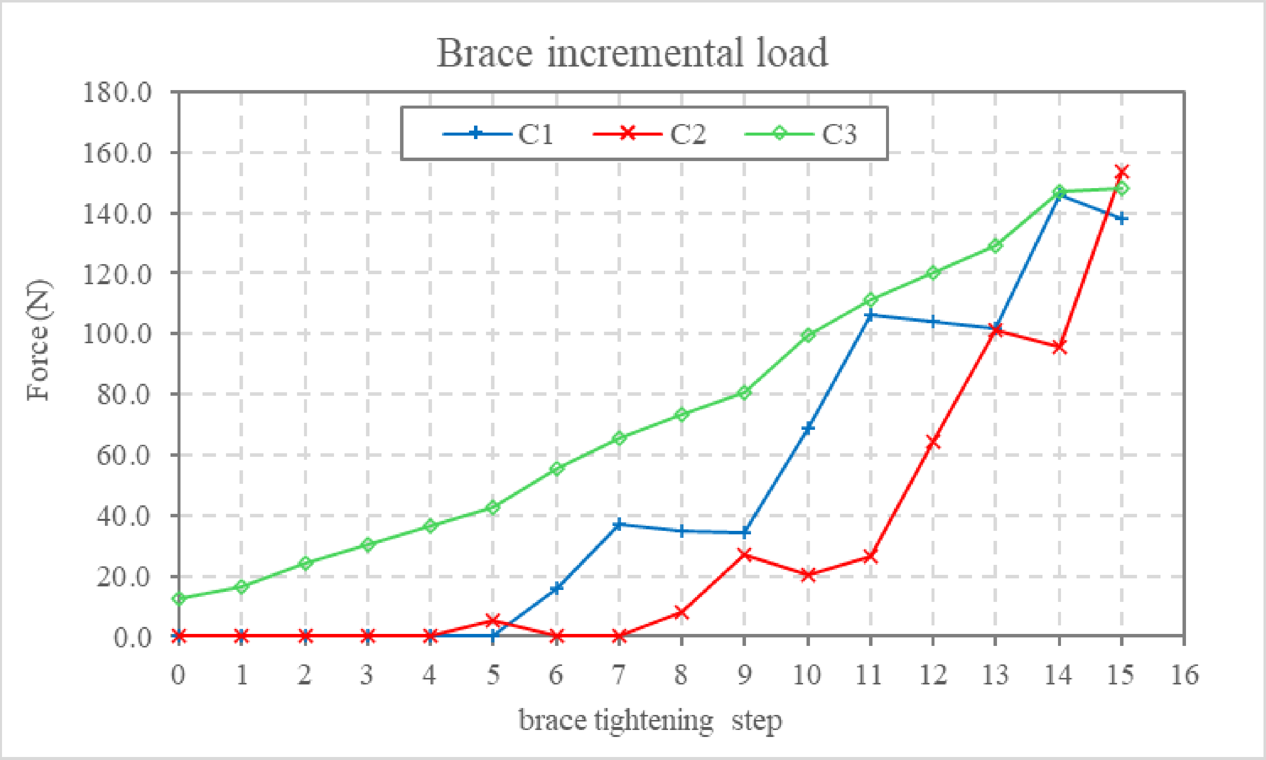

© MaCh3D s.r.l. Proprietary & Confidential

Tests with 4 mm Spacers – CTC20-D4-LD

LD		Load cell		
		Bottom	Top	Mid
Step	Buckle Id	C1	C2	C3
--	--	N	N	N
0	--	0.00	0.00	9.17
1	2S	0.00	0.00	12.75
2	1D	0.00	0.00	23.54
3	1S	5.69	0.00	29.76
4	2D	0.00	0.00	38.05
5	2S	0.00	4.51	43.59
6	1D	14.91	0.00	52.76
7	1S	33.34	0.00	64.53
8	2D	30.99	8.04	72.67
9	2S	31.97	23.93	80.22
10	1D	65.90	20.01	98.07
11	1S	101.01	20.01	113.07
12	2D	97.48	52.17	122.58
13	2S	91.01	96.30	129.94
14	1D	141.80	85.91	155.93
15	2D	132.59	148.28	154.99

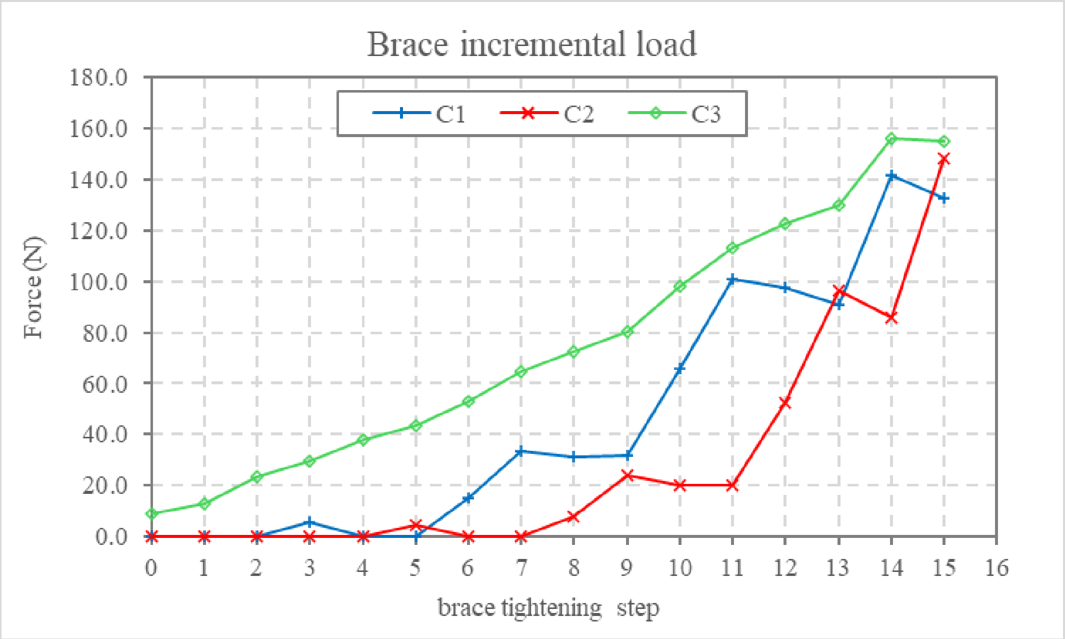

LD		Load cell		
		Bottom	Top	Mid
Step	Buckle Id	C1	C2	C3
--	--	N	N	N
0	--	0.00	0.00	8.43
1	2S	0.00	0.00	14.56
2	1D	0.00	0.00	20.01
3	1S	4.12	0.00	24.96
4	2D	0.00	0.00	36.87
5	2S	0.00	0.00	42.46
6	1D	10.00	0.00	53.96
7	1S	29.22	0.00	66.59
8	2D	25.50	4.90	73.65
9	2S	26.09	21.57	80.86
10	1D	57.47	17.85	97.82
11	1S	97.28	17.26	112.43
12	2D	85.51	52.76	122.88
13	2S	84.93	95.71	130.33
14	1D	133.37	83.94	151.51
15	2D	129.84	143.18	159.85

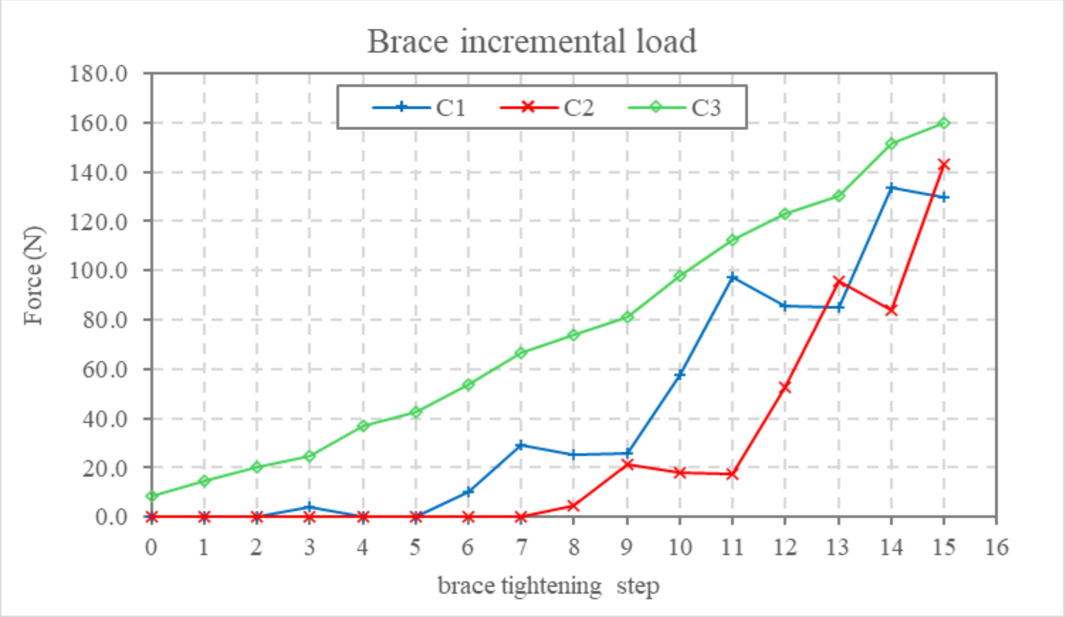

© MaCh3D s.r.l. Proprietary & Confidential

Screw Tests – CTC20-D0-LD

- Tests were carried out to evaluate the influence of the front screws on the force acting on the torso. The tests were conducted starting from a zeroing condition different from that used in the previous experiments.
- The action was applied to a single screw, monitoring the increase in force at each full turn.
- Analysis of the results shows that the force measured by the lower button load cell—corresponding to the screw location—follows an almost linear trend, thus allowing fine and continuous adjustment between the force values associated with individual clicks.

LD		Load cell		
		Bottom	Top	Mid
Step	Buckle Id	C1	C2	C3
--	--	*N*	*N*	*N*
0	--	0.000	0.000	0.000
1	2S	5.296	14.514	0.735
2	1D	15.887	13.729	2.059
3	1S	36.088	15.298	1.912
4	2D	32.950	39.815	1.667
5	g1	41.188	37.461	0.000
6	g2	51.387	36.873	1.324
7	g3	60.997	37.069	2.746
8	g4	70.608	37.265	4.217
9	g5	81.984	38.246	6.325
10	g6	94.928	39.030	7.796
11	g7	108.462	40.600	9.365
12	g8	129.840	42.169	9.758
13	g9	139.058	42.757	11.082
14	g10	152.591	44.130	11.327

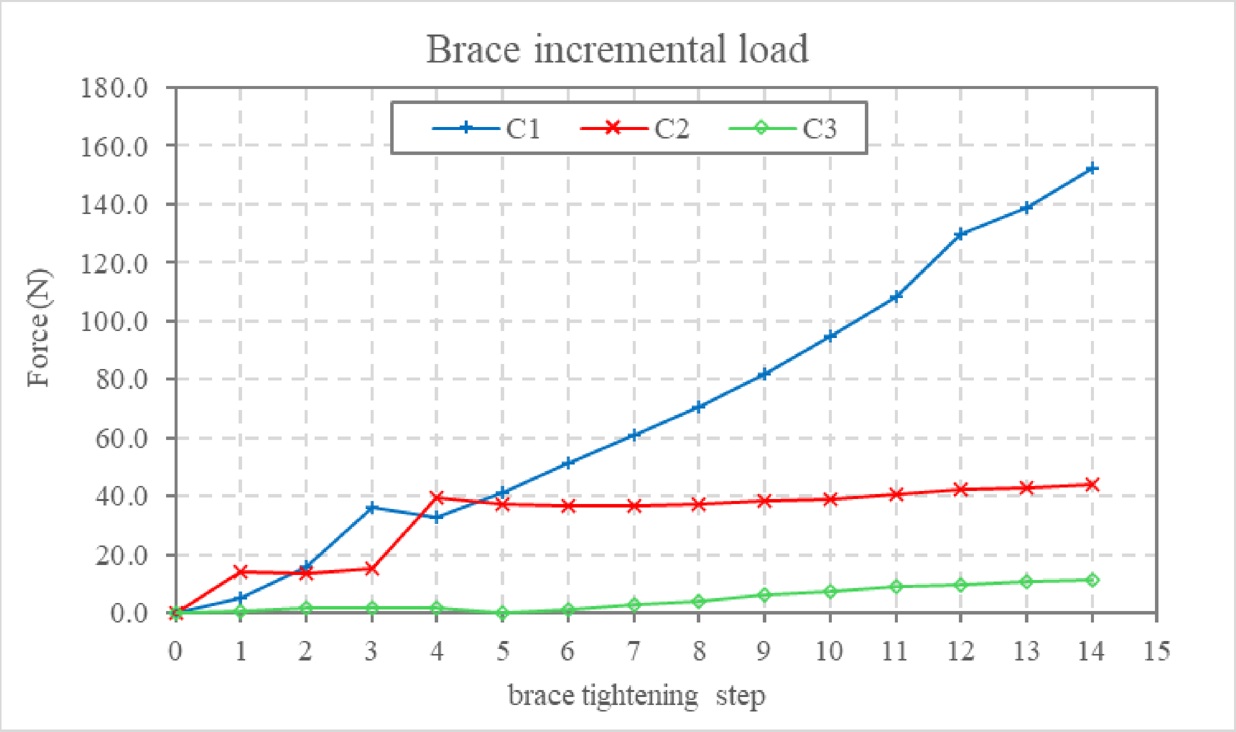

© MaCh3D s.r.l. Proprietary & Confidential

Screw Tests – CTC20-D0-LD

LD		Load cell		
		Bottom	Top	Mid
Step	Buckle Id	C1	C2	C3
--	--	*N*	*N*	*N*
0	--	0.000	0.000	0.000
1	2S	4.119	12.945	0.000
2	1D	13.141	11.964	2.108
3	1S	31.185	13.533	1.422
4	2D	29.224	36.677	1.226
5	g1	36.677	34.716	0.000
6	g2	36.285	34.323	0.735
7	g3	55.113	33.931	1.079
8	g4	64.724	34.716	1.618
9	g5	74.727	35.500	2.942
10	g6	87.671	36.088	4.315
11	g7	101.205	37.461	5.099
12	g8	115.915	38.736	6.080
13	g9	128.663	40.011	7.061
14	g10	143.569	41.580	8.434
15	g11	158.279	42.561	7.845

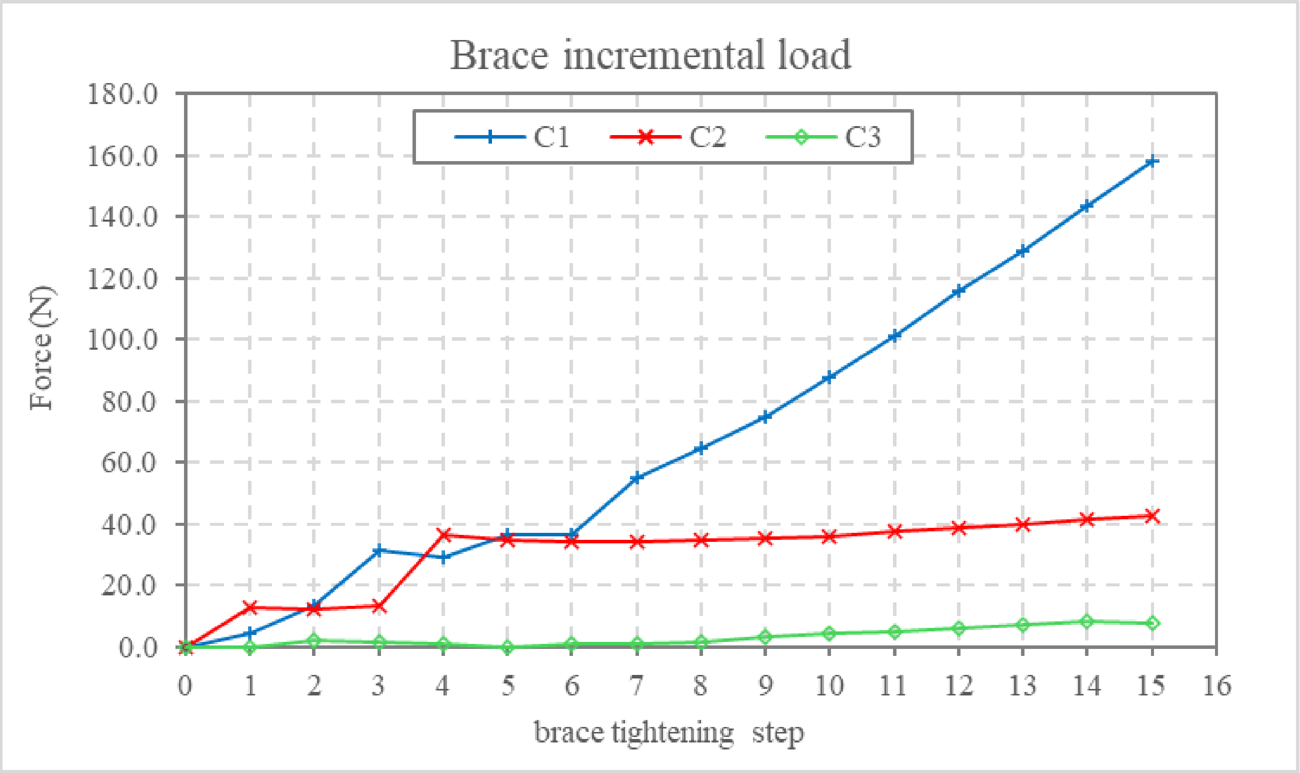

© MaCh3D s.r.l. Proprietary & Confidential

Experimental Result – 20 Kg Button Load Cells (CCC)

– *Experimental results of the tests conducted on the torso.*

© MaCh3D s.r.l. Proprietary & Confidential

Test without spacers - CCC20-D0-LD

- Three tests were carried out using the same reference setup, without spacers and with Double Laces (LD). This configuration allows the magnitude of the force applied by the brace to the torso under nominal alignment conditions to be evaluated.
- Compared to the CTC configuration, three 20 kg button load cells were used in this case. The purpose of the tests was to validate the new load cell arrangement, enabling the subsequent installation of 50 kg load cells and allowing the torso to be loaded up to its limit.
- Analysis of the graphs shows results consistent with the previous configuration (CTC). The forces measured by the top and bottom load cells exhibit an oscillating trend, resulting from the sequential execution of the clicks, performed one at a time. The mid load cell records lower force values compared to the other load cells. This behavior can be attributed to the absence of misalignment between the torso sections, confirming the correct functioning of the developed device.

LD		Load cell		
		Bottom	Top	Mid
Step	Buckle Id	C1	C2	C3
--	--	*N*	*N*	*N*
0	--	6.86	3.33	0.00
1	2S	7.85	12.16	0.00
2	1D	21.38	12.94	0.00
3	1S	46.29	14.12	0.00
4	2D	41.78	36.68	0.00
5	2S	39.42	71.98	0.00
6	1D	76.69	70.41	0.69
7	1S	133.37	73.94	2.65
8	2D	121.21	138.86	0.00

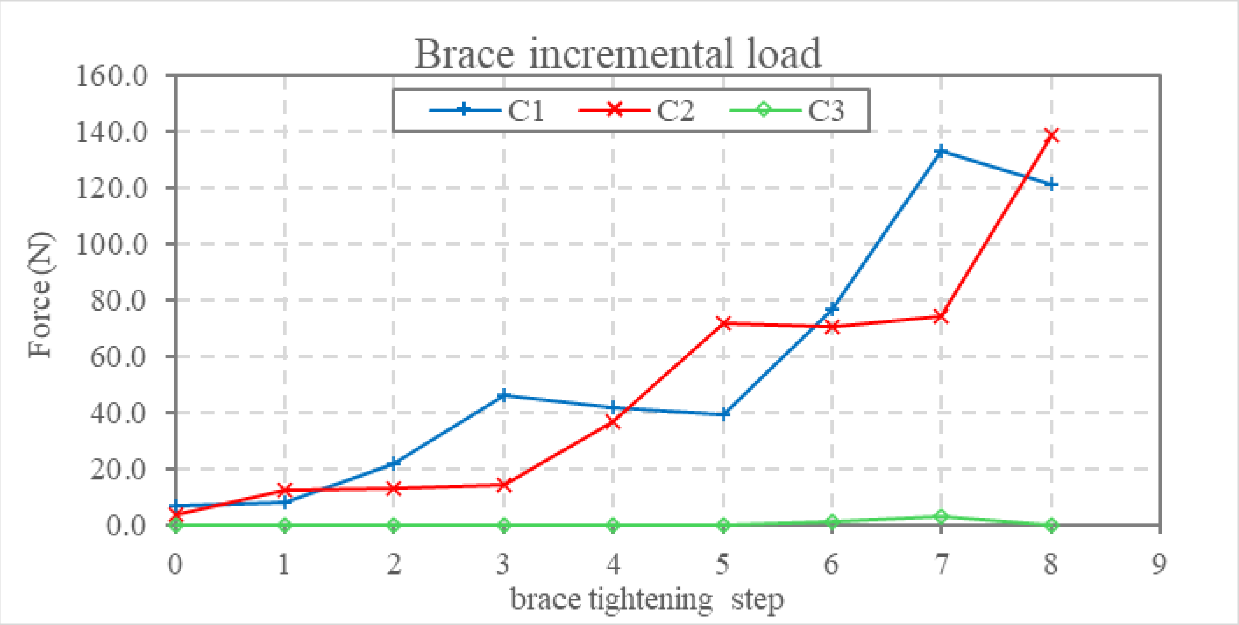

© MaCh3D s.r.l. Proprietary & Confidential

Test without spacers - CCC20-D0-LD

LD		Load cell		
		Bottom	Top	Mid
Step	Buckle Id	C1	C2	C3
--	--	N	N	N
0	--	5.88	2.75	0.00
1	2S	6.28	12.55	0.00
2	1D	18.44	11.38	1.67
3	1S	42.17	12.75	1.86
4	2D	39.62	34.32	0.00
5	2S	37.07	74.33	3.04
6	1D	73.55	71.78	5.20
7	1S	127.58	76.10	6.28
8	2D	120.03	138.27	2.06

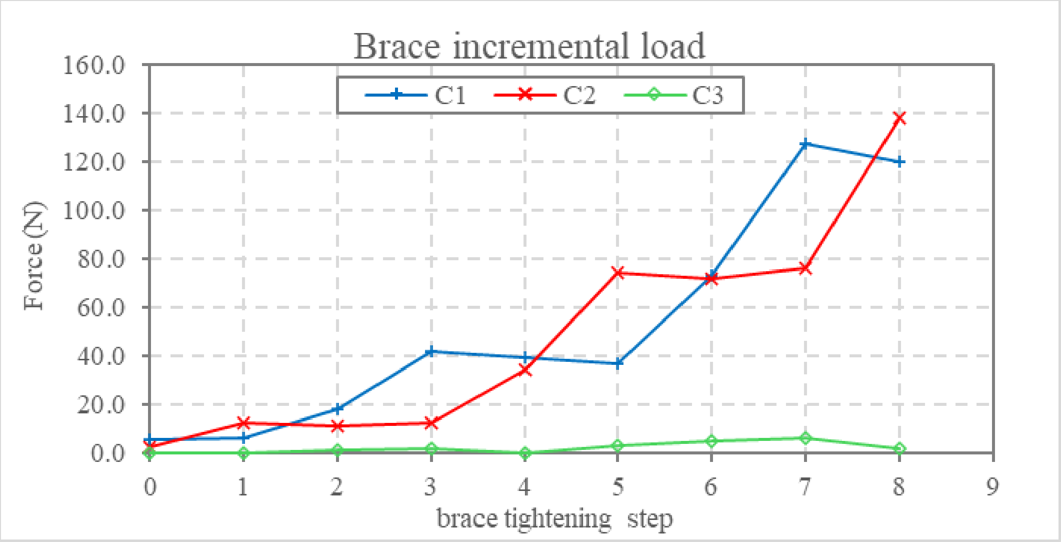

LD		Load cell		
		Bottom	Top	Mid
Step	Buckle Id	C1	C2	C3
--	--	N	N	N
0	--	6.47	2.94	0.00
1	2S	7.26	12.16	0.00
2	1D	20.99	12.36	1.77
3	1S	44.52	13.53	1.86
4	2D	40.80	37.27	0.00
5	2S	36.87	72.96	4.31
6	1D	73.75	71.39	4.90
7	1S	127.88	75.71	4.12
8	2D	112.58	142.20	0.00

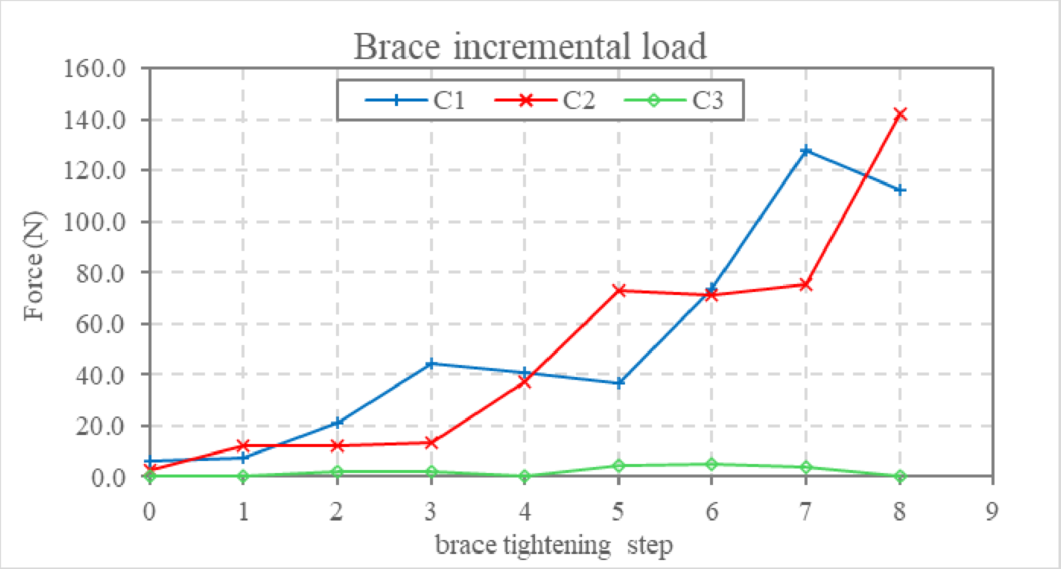

© MaCh3D s.r.l. Proprietary & Confidential

Test without spacers - CCC20-D0-LDB

- Three tests were conducted using the same reference setup, without spacers and with Double Bonded Laces (LDB), in order to assess the influence of different lace configurations on the magnitude of the force applied to the torso.
- Compared to the non-bonded double laces configuration (LD), the bonded laces allows higher force levels to be achieved at the same number of clicks, owing to its lower compliance.

LDB		Load cell		
		Bottom	Top	Mid
Step	Buckle Id	C1	C2	C3
--	--	*N*	*N*	*N*
0	--	6.28	5.30	2.94
1	2S	6.28	21.77	6.77
2	1D	19.22	20.20	6.37
3	1S	45.11	22.36	3.33
4	2D	41.19	58.94	9.51
5	2S	34.91	109.64	11.87
6	1D	74.73	106.30	13.73
7	1S	133.37	109.44	8.63
8	2D	117.68	179.07	19.42
9	1D	172.79	174.95	19.42

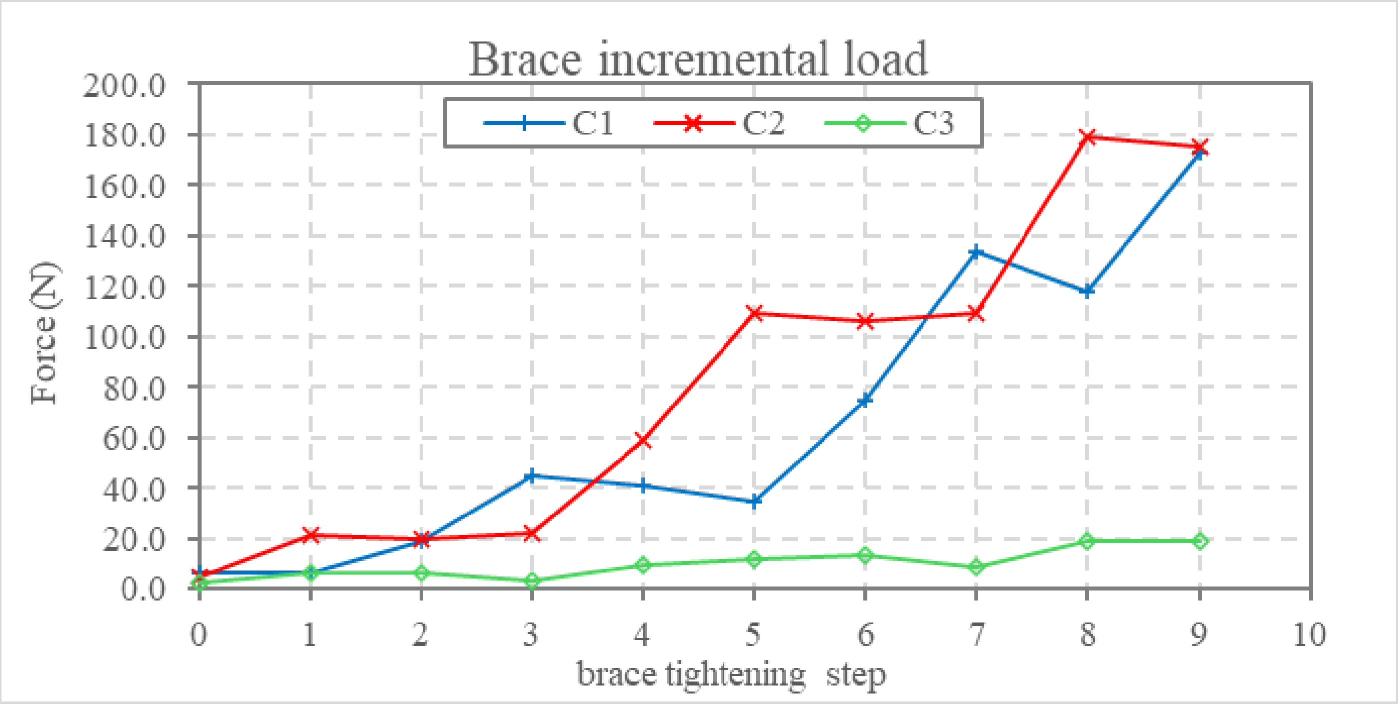

© MaCh3D s.r.l. Proprietary & Confidential

Test without spacers - CCC20-D0-LDB

LDB		Load cell		
		Bottom	Top	Mid
Step	Buckle Id	C1	C2	C3
--	--	*N*	*N*	*N*
0	--	8.04	5.49	2.06
1	2S	7.45	20.79	3.82
2	1D	20.59	20.20	6.28
3	1S	45.90	21.38	6.47
4	2D	40.99	56.49	6.08
5	2S	36.28	108.07	9.12
6	1D	75.32	105.91	10.59
7	1S	132.39	109.83	12.06
8	2D	120.03	183.58	3.82
9	1D	174.75	174.56	15.30

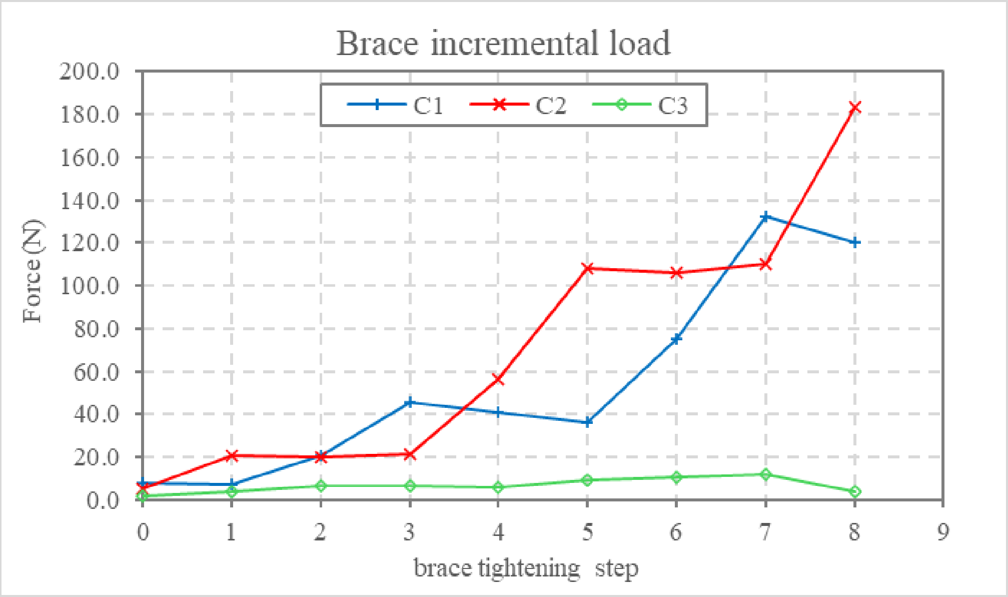

LDB		Load cell		
		Bottom	Top	Mid
Step	Buckle Id	C1	C2	C3
--	--	*N*	*N*	*N*
0	--	9.61	5.49	1.67
1	2S	9.61	22.95	3.24
2	1D	22.56	22.16	5.39
3	1S	47.86	25.30	2.55
4	2D	44.13	61.78	1.86
5	2S	37.27	108.07	10.10
6	1D	75.51	103.36	12.94
7	1S	134.35	113.76	6.77
8	2D	123.56	187.31	10.00
9	1D	181.62	182.40	6.77

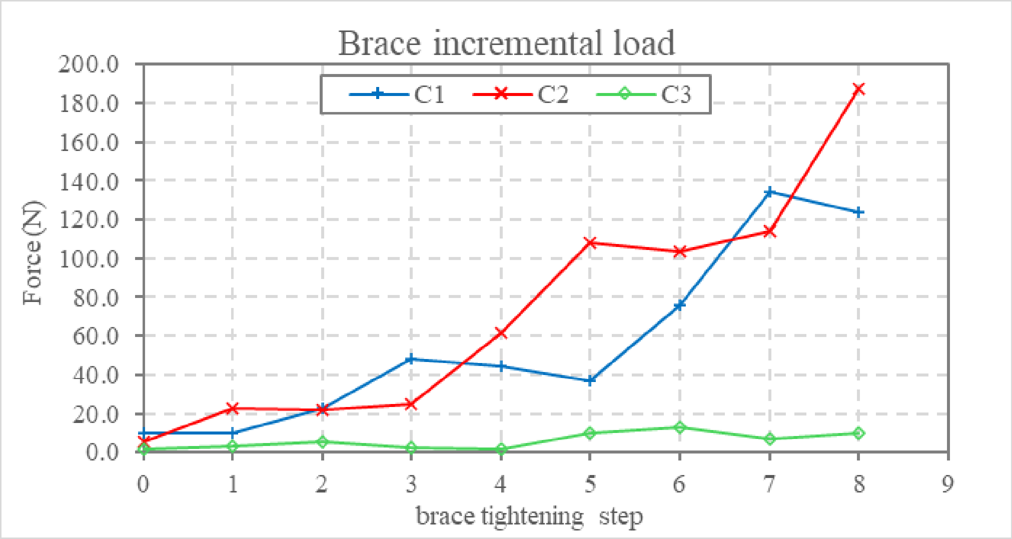

© MaCh3D s.r.l. Proprietary & Confidential

Test without spacers - CCC20-D0-LS

- Three tests were conducted using the same reference setup, without spacers and with Single Laces (LS), in order to assess the influence of different lace configurations on the magnitude of the force applied to the torso.
- Compared to the non-bonded double lace configuration (LD), lower force levels are achieved for the same number of clicks due to the higher compliance of the single lace.

LS		Load cell		
		Bottom	Top	Mid
Step	Buckle Id	C1	C2	C3
--	--	*N*	*N*	*N*
0	--	4.31	3.14	1.67
1	2S	4.90	15.49	4.02
2	1D	14.91	14.91	1.47
3	1S	37.07	17.65	1.57
4	2D	32.95	39.23	11.28
5	2S	28.83	68.74	10.79
6	1D	57.96	67.67	6.77
7	1S	101.60	74.73	7.26
8	2D	90.22	127.68	12.55
9	1D	143.18	119.44	12.55

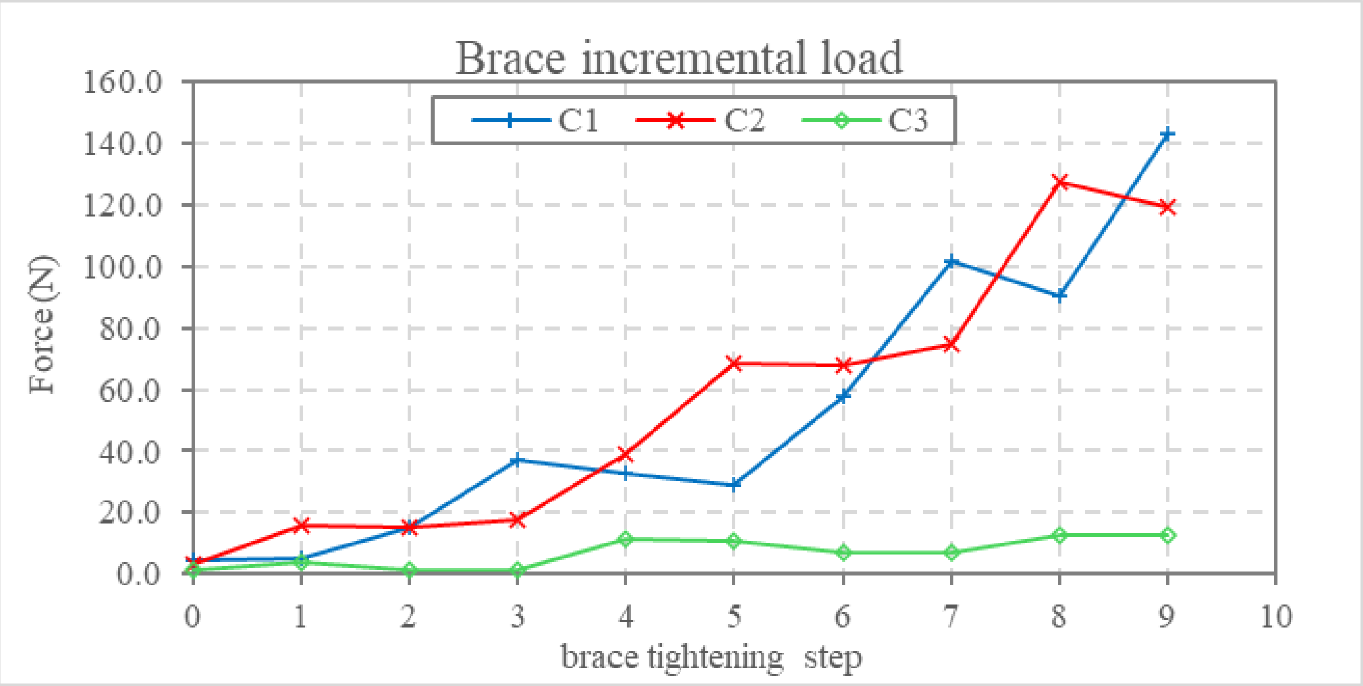

© MaCh3D s.r.l. Proprietary & Confidential

Test without spacers - CCC20-D0-LS

LS		Load cell		
		Bottom	Top	Mid
Step	Buckle Id	C1	C2	C3
--	--	*N*	*N*	*N*
0	--	3.92	3.73	1.47
1	2S	4.12	15.89	2.55
2	1D	12.55	14.12	5.10
3	1S	32.75	15.49	4.71
4	2D	30.01	34.32	4.41
5	2S	26.67	65.90	8.34
6	1D	52.56	64.14	9.61
7	1S	99.24	67.86	10.49
8	2D	89.24	125.72	7.06
9	1D	143.18	123.17	13.63

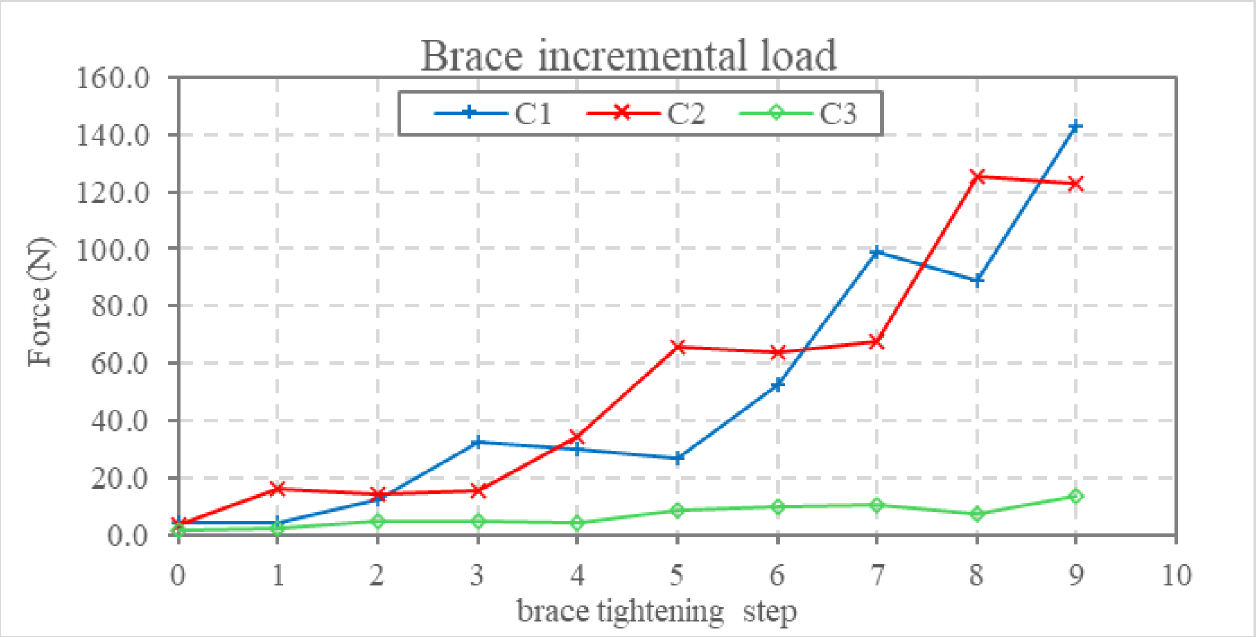

LS		Load cell		
		Bottom	Top	Mid
Step	Buckle Id	C1	C2	C3
--	--	*N*	*N*	*N*
0	--	3.33	3.73	2.06
1	2S	3.33	12.94	3.24
2	1D	10.98	12.36	5.39
3	1S	30.20	14.32	2.55
4	2D	27.85	31.38	1.86
5	2S	25.30	65.70	10.10
6	1D	52.96	65.70	12.94
7	1S	95.61	67.67	6.77
8	2D	87.28	122.58	10.00
9	1D	141.22	119.44	6.77

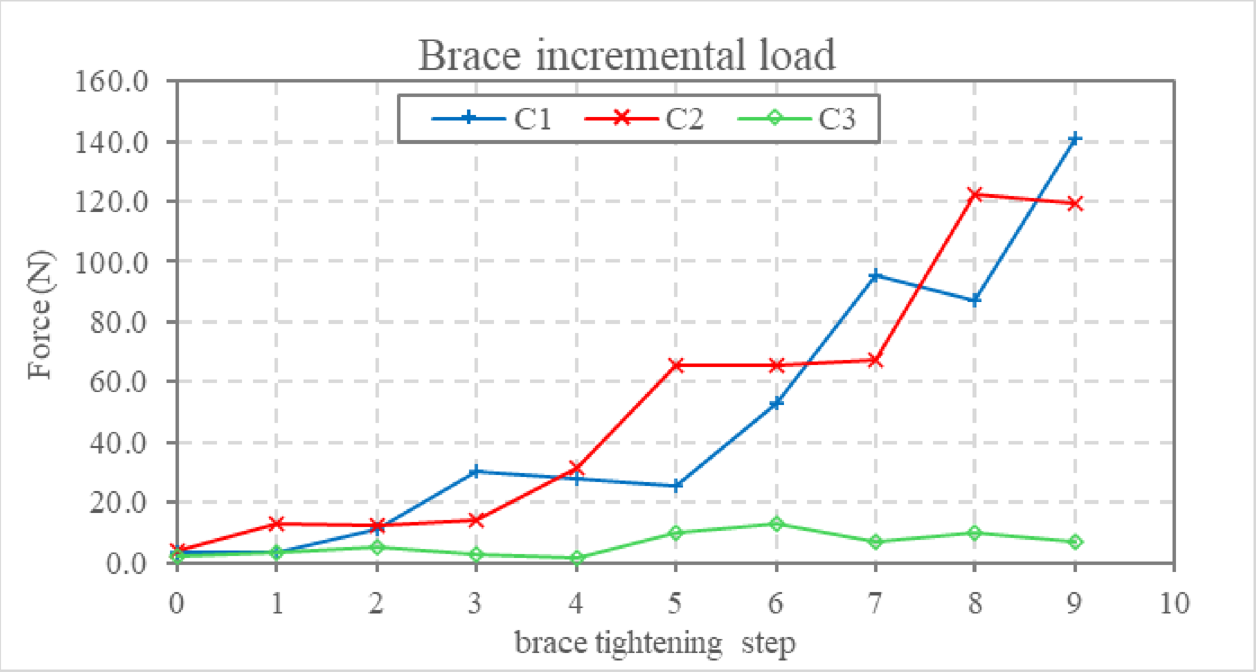

© MaCh3D s.r.l. Proprietary & Confidential

Test without spacers - CCC20-D0-NL

- Tests were carried out with the brace straps removed in order to assess their influence on the forces measured by the load cells.
- The results show that the maximum force levels remain unchanged; however, as expected, in the absence of the straps a higher number of clicks is required due to the increased deformation of the neoprene.

LD		Load cell		
		Bottom	Top	Mid
Step	Buckle Id	C1	C2	C3
--	--	*N*	*N*	*N*
0	--	3.92	3.33	1.08
1	2S	2.75	9.81	3.14
2	1D	9.61	9.61	4.02
3	1S	22.16	11.18	2.65
4	2D	21.77	25.11	0.00
5	2S	20.99	39.23	2.06
6	1D	36.28	38.44	3.04
7	1S	58.45	39.62	3.53
8	2D	53.35	62.76	3.53
9	1D	51.58	78.45	3.63
10	1S	81.20	82.38	2.55
11	2D	104.34	87.48	2.35
12	2S	93.56	113.76	4.51
13	1D	92.18	122.6	5.69
14	1S	134.35	131.21	0.00
15	2D	157.89	132.6	0.00
16	2S	145.14	166.81	7.94
17	1D	141.61	176.13	7.16
18	1S	182.40	181.82	0.00

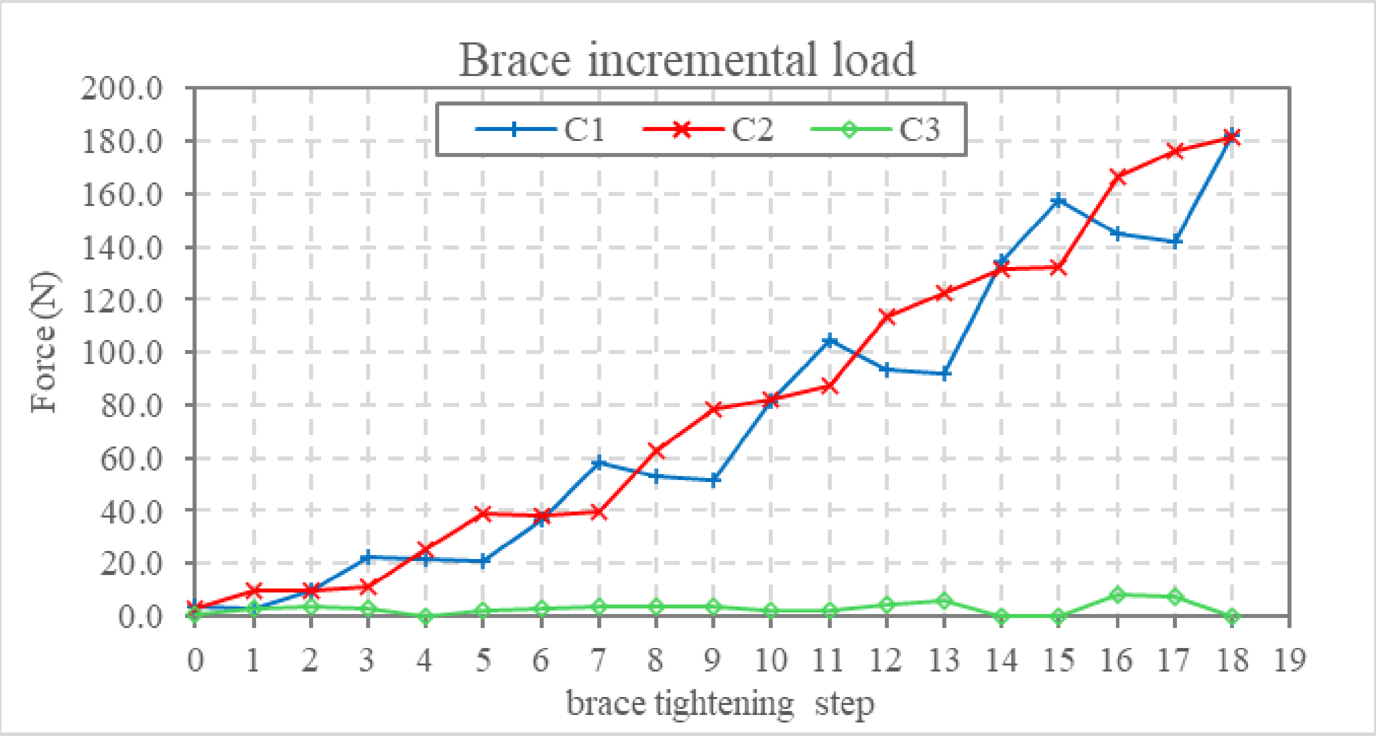

© MaCh3D s.r.l. Proprietary & Confidential

Test with spacers - CCC20-D2-LD

- Three tests were carried out using 3D-printed spacers with a thickness of 2 mm.
- This setup allows for the evaluation of the shear force that the brace can apply to the torso in the presence of a controlled and limited misalignment.
- By comparing the results with the previous diagrams, it becomes evident that, in this case, the shear force assumes a significantly higher value, confirming the influence of misalignment on the distribution of forces among the load cells.
- The trend of the forces is the same as observed in other similar tests with different load cells configuration.

LD		Load cell		
		Bottom	Top	Mid
Step	Buckle Id	C1	C2	C3
--	--	*N*	*N*	*N*
0	--	0.00	0.00	21.97
1	2S	0.00	5.10	28.93
2	1D	0.00	6.86	36.77
3	1S	9.41	6.28	48.05
4	2D	10.79	24.91	48.35
5	2S	0.00	58.84	62.96
6	1D	28.05	56.88	74.53
7	1S	70.80	57.27	84.93
8	2D	63.74	117.88	87.28
9	2S	46.48	163.77	99.24
10	1D	107.68	161.42	116.40
11	1S	153.96	175.54	123.17

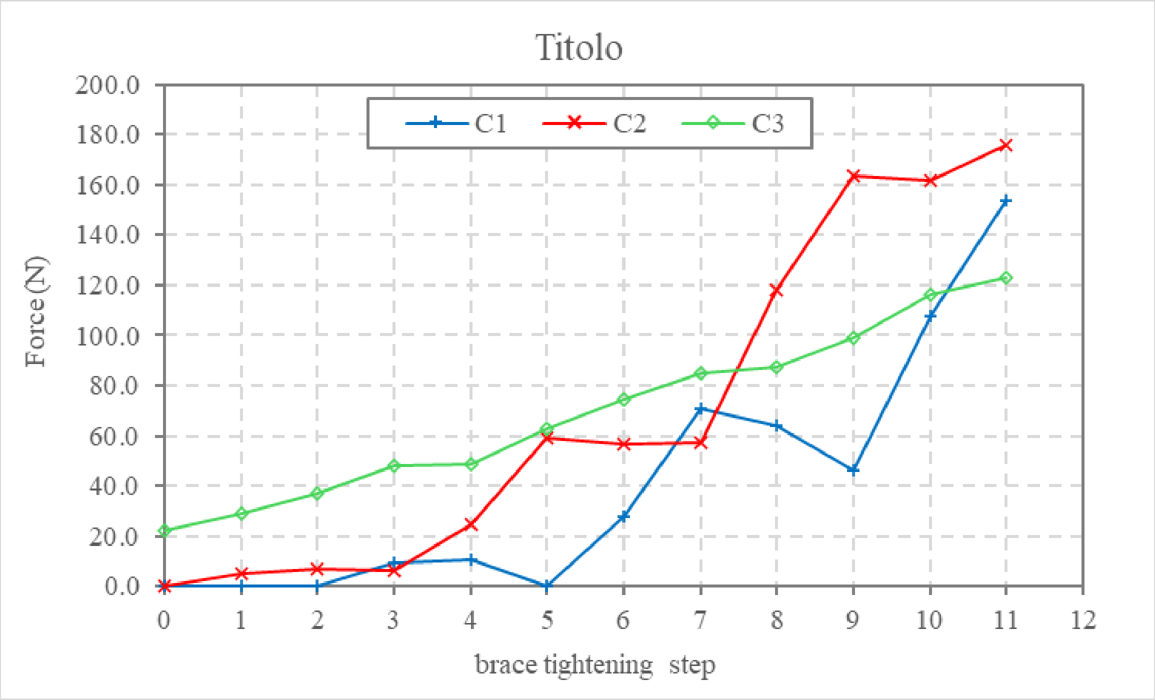

© MaCh3D s.r.l. Proprietary & Confidential

Test with spacers - CCC20-D2-LD

LD		Load cell		
		Bottom	Top	Mid
Step	Buckle Id	C1	C2	C3
--	--	*N*	*N*	*N*
0	--	0.00	0.00	21.87
1	2S	0.00	2.75	26.09
2	1D	0.00	2.55	35.89
3	1S	8.63	0.00	47.86
4	2D	4.71	12.94	53.05
5	2S	0.00	50.01	62.08
6	1D	28.64	49.43	68.16
7	1S	54.72	40.01	89.24
8	2D	56.29	104.44	79.04
9	2S	51.58	170.24	85.02
10	1D	105.91	163.97	103.95
11	1S	153.96	170.64	121.80

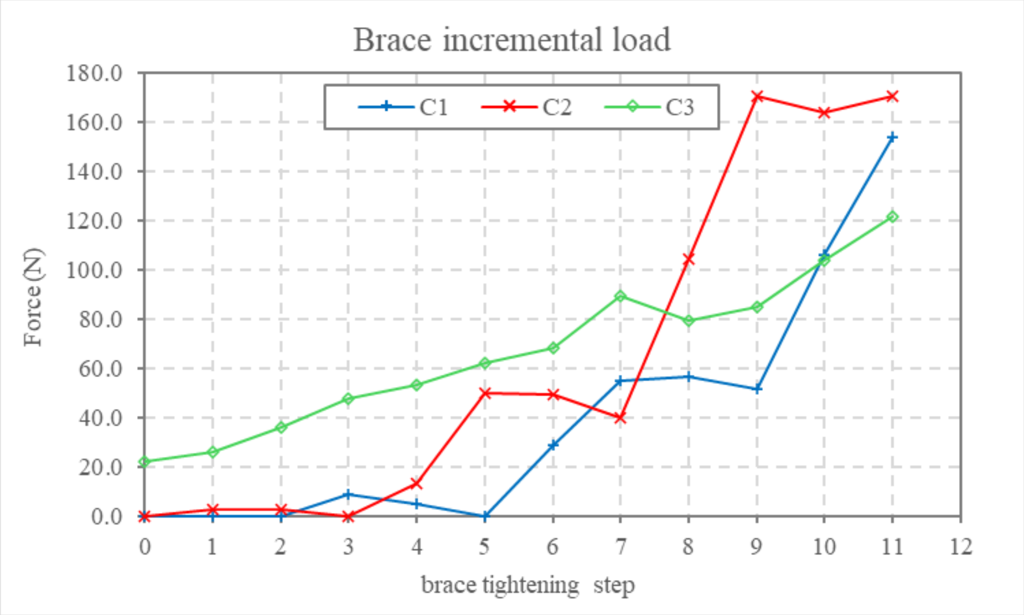

LD		Load cell		
		Bottom	Top	Mid
Step	Buckle Id	C1	C2	C3
--	--	*N*	*N*	*N*
0	--	0.00	0.00	16.67
1	2S	0.00	4.51	20.40
2	1D	0.00	3.14	29.81
3	1S	10.00	0.00	40.01
4	2D	8.63	15.49	43.25
5	2S	0.00	47.27	56.09
6	1D	20.01	40.99	67.47
7	1S	59.43	39.62	74.82
8	2D	58.06	102.97	71.69
9	2S	52.37	163.57	77.86
10	1D	105.52	157.30	98.56
11	1S	150.43	151.41	125.13

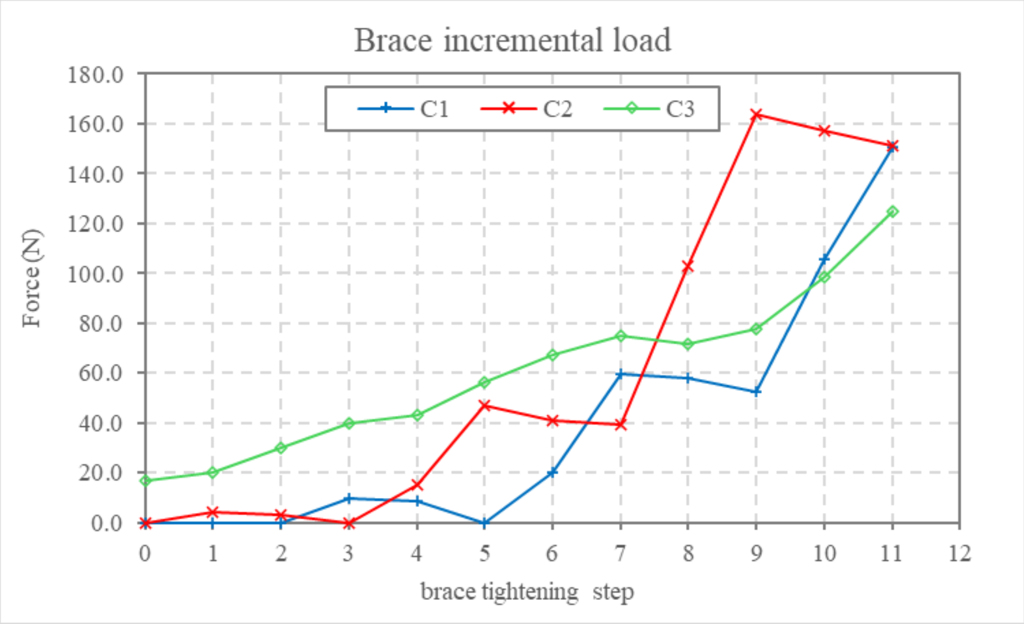

© MaCh3D s.r.l. Proprietary & Confidential

Test with spacers - CCC20-D4-LD

- Three tests were carried out using the same setup, employing 3D-printed spacers with a thickness of 4 mm.
- This configuration allows for the evaluation of the shear force that the brace can apply to the torso under conditions of pronounced misalignment.
- From the analysis of the diagrams, it can be observed that during the first five or six clicks, the bottom and the top load cells do not register any significant reading, whereas the shear load cell records consistently high force values. This behavior confirms the predominance of the shear contribution (corrective force) in the presence of a marked misalignment between the torso sections.
- The trend of the forces is the same as observed in other similar tests with different load cells configuration.

LD		Load cell		
		Bottom	Top	Mid
Step	Buckle Id	C1	C2	C3
--	--	*N*	*N*	*N*
0	--	0.00	0.00	5.10
1	2S	0.00	0.00	10.30
2	1D	0.00	0.00	18.24
3	1S	7.85	0.00	23.73
4	2D	7.85	0.00	30.20
5	2S	5.88	5.88	35.79
6	1D	20.01	2.55	51.58
7	1S	44.13	0.00	66.00
8	2D	41.78	8.63	72.18
9	2S	37.27	35.70	80.61
10	1D	73.55	28.64	103.95
11	1S	104.93	28.24	120.62
12	2D	98.46	64.53	126.70
13	2S	92.77	113.76	147.10
14	1D	147.59	104.15	170.05
15	2D	141.41	163.77	173.68

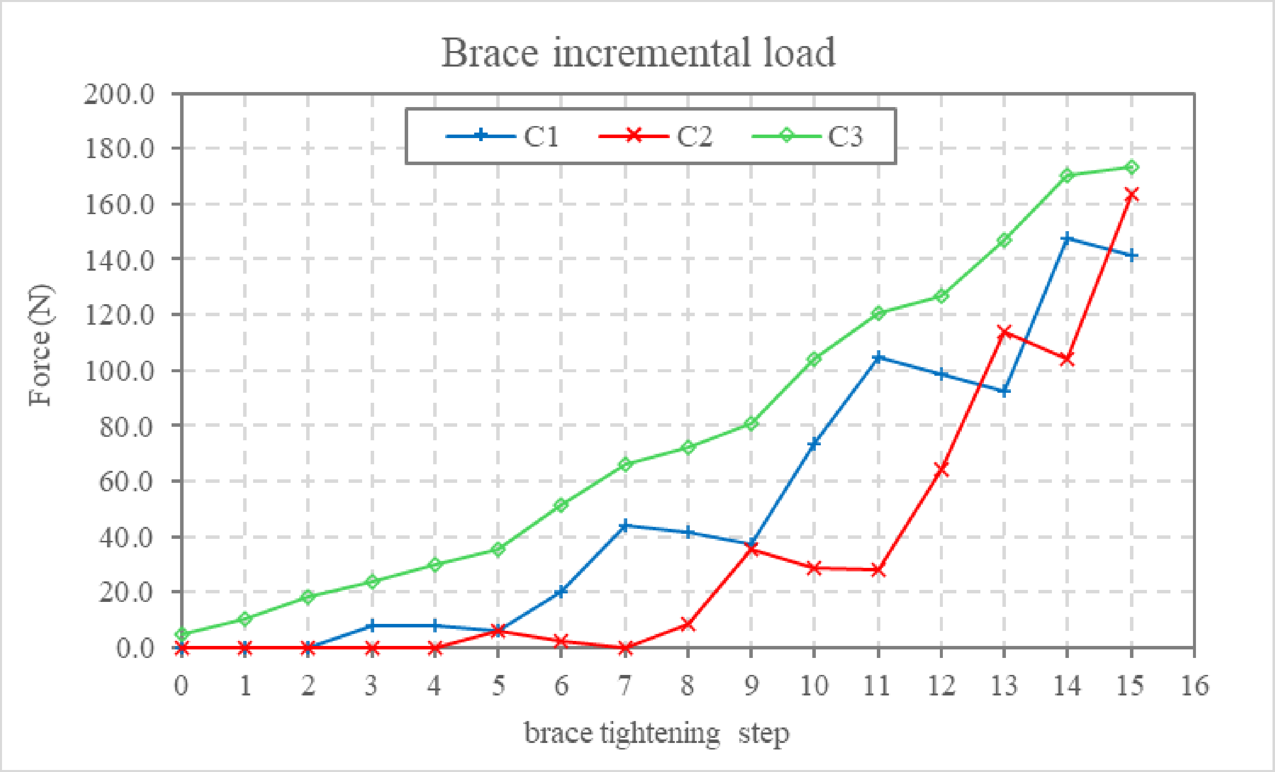

© MaCh3D s.r.l. Proprietary & Confidential

Test with spacers - CCC20-D4-LD

LD		Load cell		
		Bottom	Top	Mid
Step	Buckle Id	C1	C2	C3
--	--	*N*	*N*	*N*
0	--	0.00	0.00	16.18
1	2S	2.35	0.00	10.79
2	1D	7.65	0.00	17.55
3	1S	7.26	0.00	37.95
4	2D	13.53	6.47	40.11
5	2S	12.55	20.40	45.31
6	1D	28.05	11.96	65.80
7	1S	56.88	12.94	77.77
8	2D	52.56	41.97	81.79
9	2S	49.03	79.04	89.83
10	1D	86.69	73.55	112.97
11	1S	127.88	77.86	138.96
12	2D	123.56	131.21	148.67
13	2S	118.46	182.21	154.65
14	1D	132.64	141.71	165.03

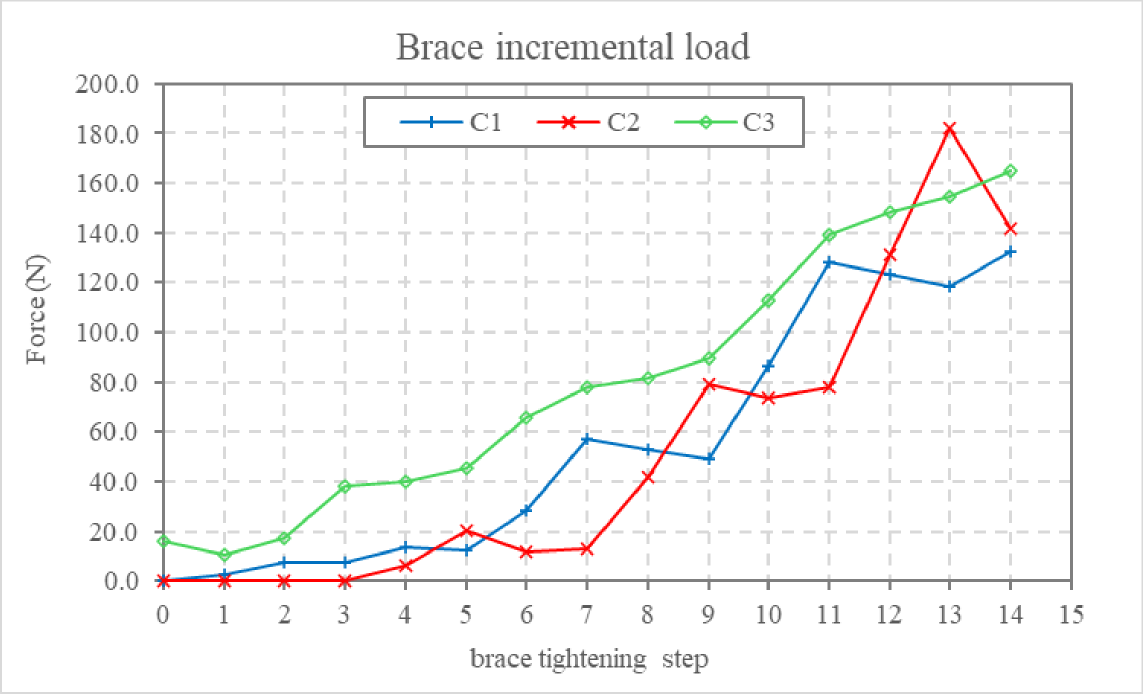

© MaCh3D s.r.l. Proprietary & Confidential

Test with spacers - CCC20-D4-LD

LD		Load cell		
		Bottom	Top	Mid
Step	Buckle Id	C1	C2	C3
--	--	N	N	N
0	--	0.00	0.00	8.53
1	2S	0.00	0.00	9.81
2	1D	0.00	0.00	24.32
3	1S	0.00	0.00	25.50
4	2D	0.00	0.00	29.22
5	2S	0.00	0.00	49.03
6	1D	14.32	0.00	64.33
7	1S	39.81	0.00	75.90
8	2D	41.78	5.69	81.00
9	2S	40.80	39.42	82.38
10	1D	71.00	31.38	101.01
11	1S	117.88	37.66	119.94
12	2D	108.46	72.96	123.56
13	2S	100.81	124.54	145.53
14	1D	147.88	117.48	170.54
15	2D	143.96	180.93	167.50

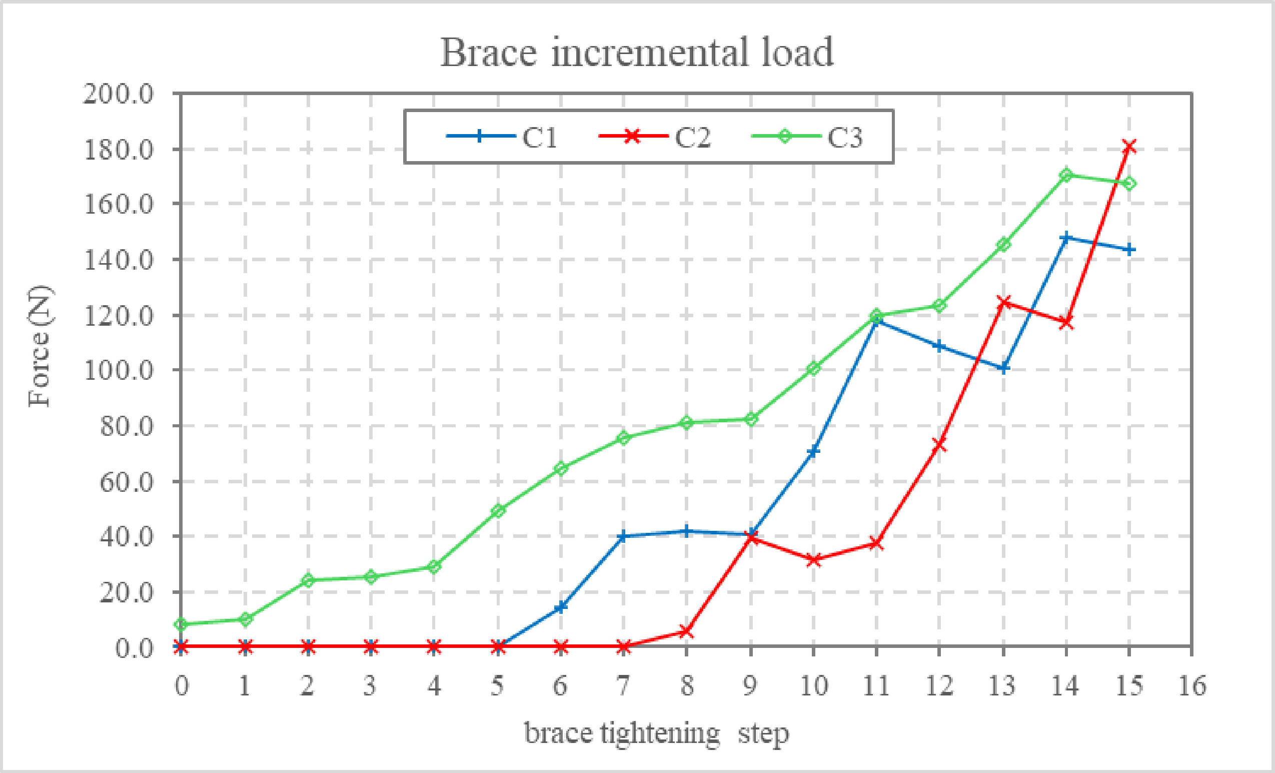

© MaCh3D s.r.l. Proprietary & Confidential

Experimental Result – 50 Kg Load Cells (CCC)

- *Experimental results of the tests conducted on the torso.*

© MaCh3D s.r.l. Proprietary & Confidential

Tests without Spacers – CCC50-D0-LD

- Three tests were carried out using the same setup with Double Laces (LD). This configuration allows the maximum magnitude of the force exerted by the brace on the torso under nominal alignment conditions to be evaluated.
- The forces measured present an oscillating trend like the previous test, resulting from the sequential execution of the clicks, performed one at a time. The mid load cell records lower force values compared to the other load cells. This behavior can be attributed to the absence of misalignment between the torso sections, confirming the correct functioning of the developed device.
- The trend is the same as in similar tests, but with higher load levels.

LD		Load cell		
		Bottom	Top	Mid
Step	Buckle Id	C1	C2	C3
--	--	*N*	*N*	*N*
0	--	4.12	3.73	3.04
1	2S	3.53	14.71	4.41
2	1D	13.14	12.94	6.77
3	1S	32.75	13.73	6.18
4	2D	28.44	31.77	5.98
5	2S	24.71	56.88	13.53
6	1D	48.44	60.02	16.57
7	1S	91.40	60.02	16.48
8	2D	83.94	112.78	21.48
9	2S	79.24	178.68	24.52
10	1D	127.49	176.72	26.09
11	1S	188.48	180.64	22.95
12	2D	176.91	259.48	24.32
13	2S	162.40	297.73	36.28
14	1D	231.63	303.61	39.13
15	1S	300.28	299.89	27.65
16	2D	286.158	377.556	40.109
17	1D	354.61	356.18	38.34

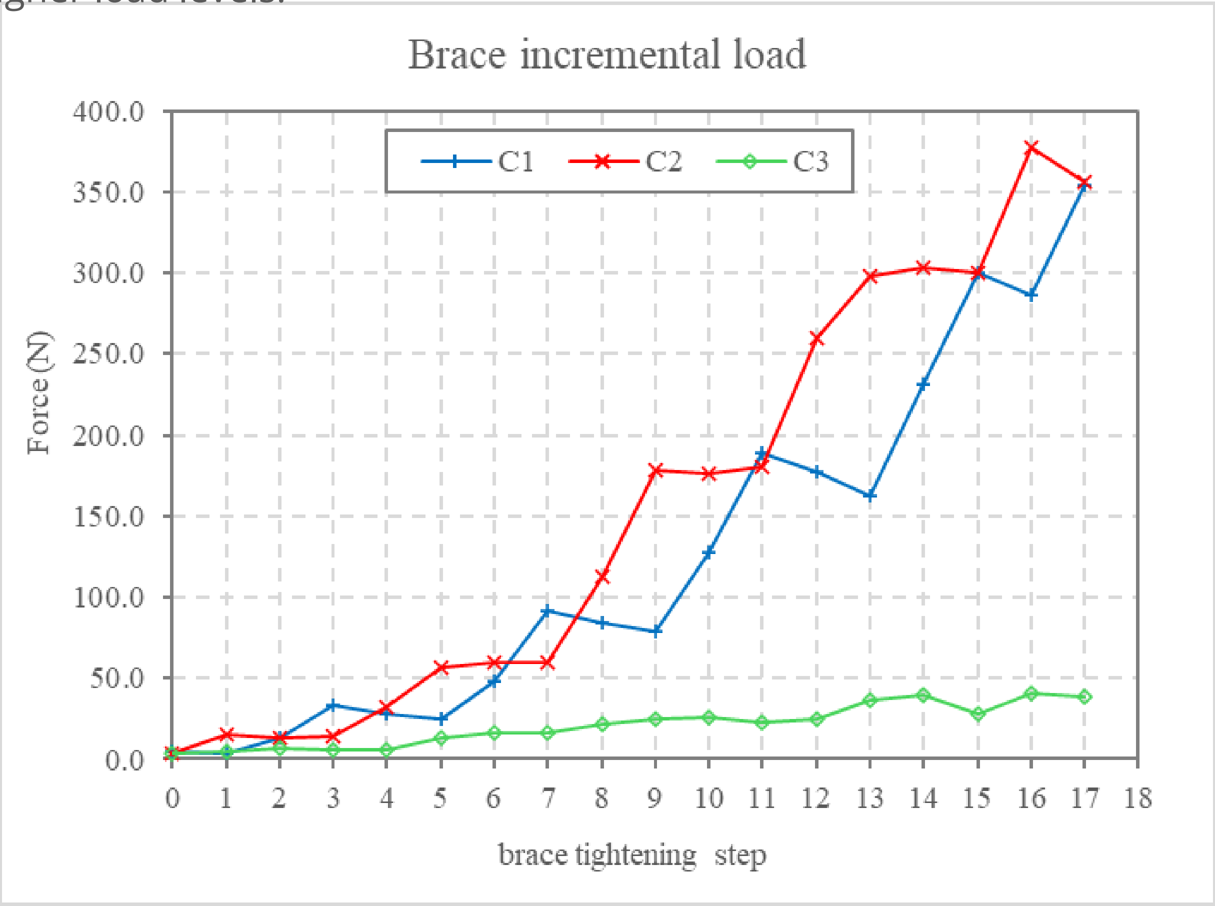

© MaCh3D s.r.l. Proprietary & Confidential

Tests without Spacers – CCC50-D0-LD

LD		Load cell		
		Bottom	Top	Mid
Step	Buckle Id	C1	C2	C3
--	--	*N*	*N*	*N*
0	--	2.35	1.96	1.67
1	2S	0.00	7.45	4.31
2	1D	6.86	5.88	6.28
3	1S	23.34	6.08	5.88
4	2D	19.81	20.20	6.77
5	2S	16.67	40.01	14.61
6	1D	39.42	43.35	17.06
7	1S	77.47	45.11	12.85
8	2D	65.70	89.44	17.06
9	2S	60.02	148.08	22.65
10	1D	104.15	147.30	26.87
11	1S	171.13	153.18	24.03
12	2D	163.77	239.48	1.96
13	2S	145.33	294.40	33.05
14	1D	216.73	297.34	38.15
15	1S	289.49	290.47	23.63
16	2D	271.6442	373.4372	34.912
17	1D	337.15	334.80	42.46

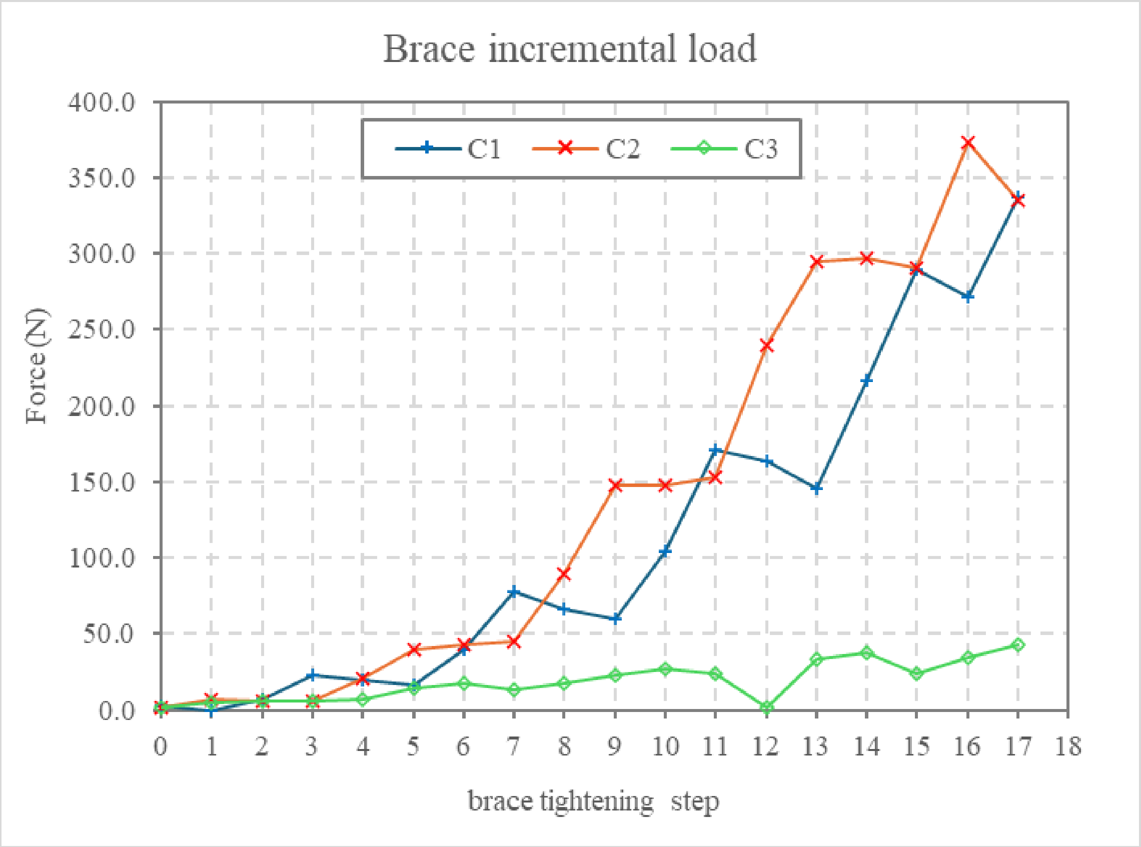

© MaCh3D s.r.l. Proprietary & Confidential

Tests without Spacers – CCC50-D0-LD

LD		Load cell		
		Bottom	Top	Mid
Step	Buckle Id	C1	C2	C3
--	--	*N*	*N*	*N*
0	--	2.55	0.00	0.49
1	2S	2.35	5.88	2.65
2	1D	8.83	4.31	4.71
3	1S	23.73	4.90	4.41
4	2D	23.34	19.81	0.00
5	2S	20.59	40.60	6.67
6	1D	42.56	41.58	8.53
7	1S	85.51	48.44	0.00
8	2D	76.69	87.87	6.96
9	2S	69.63	147.30	13.04
10	1D	112.58	143.37	15.98
11	1S	175.74	148.28	12.65
12	2D	161.03	235.56	0.00
13	2S	152.20	298.32	19.81
14	1D	219.28	298.51	28.34
15	1S	292.43	287.92	14.32
16	2D	284.79	367.95	23.24
17	1D	351.27	350.69	33.93

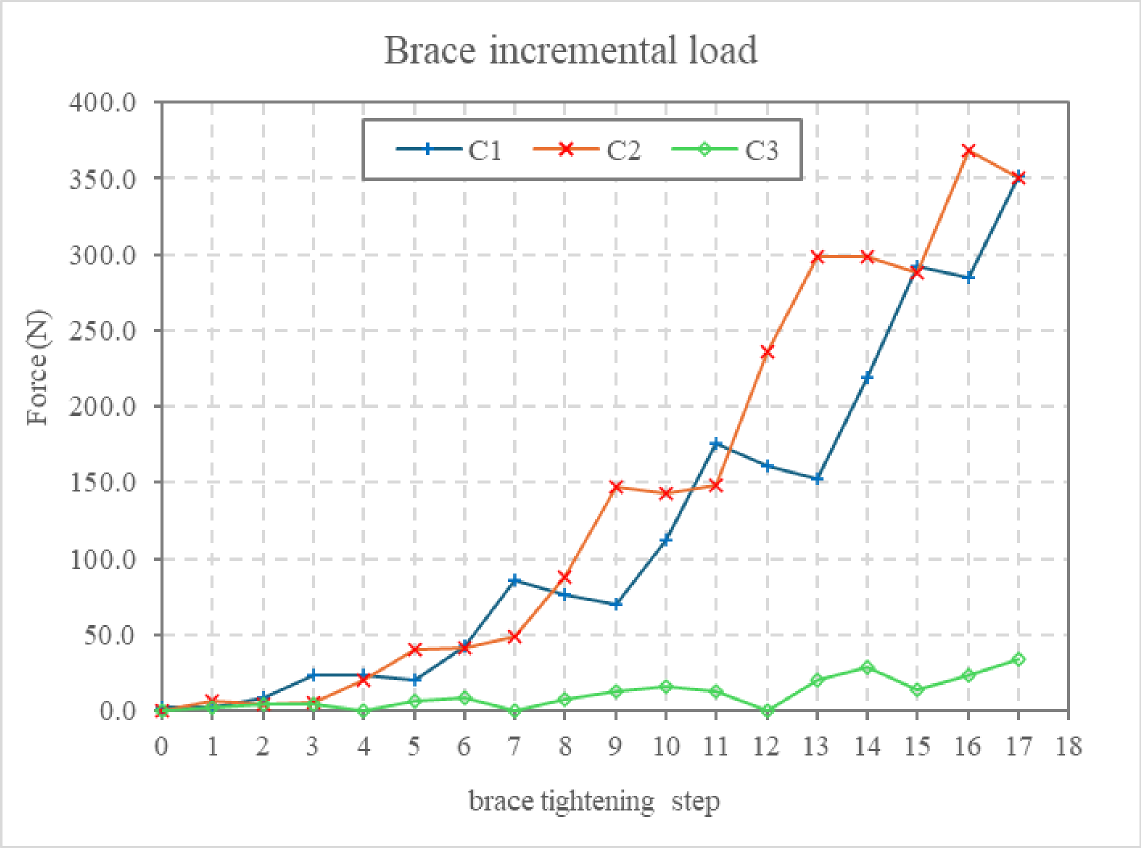

© MaCh3D s.r.l. Proprietary & Confidential

Tests without Spacers – CCC50-D0-LDB

- Three tests were carried out using the same setup with Double Bonded Laces (LDB). This configuration allows the maximum magnitude of the force exerted by the brace on the torso under nominal alignment conditions to be evaluated.
- The forces measured present an oscillating trend like the previous test, resulting from the sequential execution of the clicks, performed one at a time. The bonded laces allows higher force levels to be achieved at the same number of clicks compare to the non-bonded laces (LD), owing to its lower compliance.
- The mid load cell records lower force values compared to the other load cells. This behavior can be attributed to the absence of misalignment between the torso sections, confirming the correct functioning of the developed device.

LDB		Load cell		
		Bottom	Top	Mid
Step	Buckle Id	C1	C2	C3
--	--	*N*	*N*	*N*
0	--	4.71	3.53	0.88
1	2S	4.12	12.75	3.33
2	1D	14.71	11.18	6.47
3	1S	36.48	13.14	5.49
4	2D	34.13	38.25	0.00
5	2S	30.60	73.55	6.47
6	1D	55.90	76.10	10.49
7	1S	100.03	77.67	4.02
8	2D	91.79	136.51	10.49
9	2S	85.71	207.12	13.24
10	1D	142.98	203.39	17.26
11	1S	208.88	201.23	13.14
12	2D	193.19	283.41	0.00
13	2S	180.25	330.48	24.03
14	1D	252.82	336.37	26.87

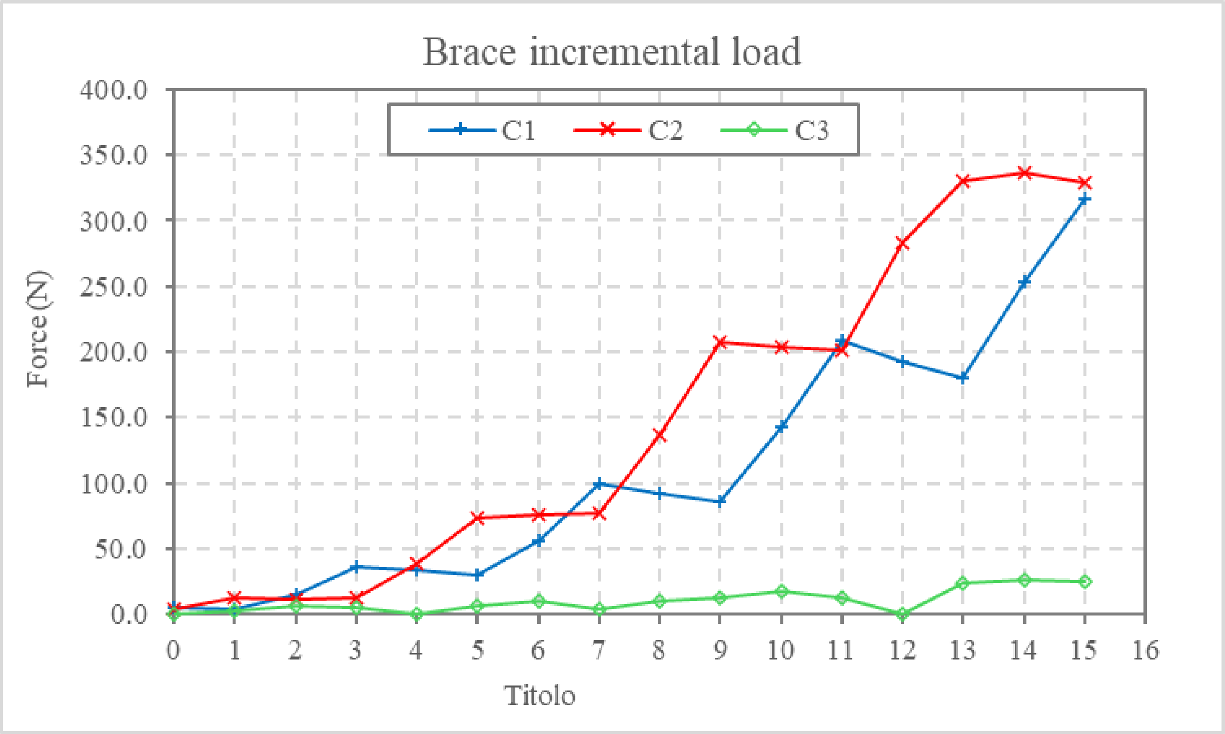

© MaCh3D s.r.l. Proprietary & Confidential

Tests without Spacers – CCC50-D0-LDB

LDB		Load cell		
		Bottom	Top	Mid
Step	Buckle Id	C1	C2	C3
--	--	*N*	*N*	*N*
0	--	4.71	3.92	1.96
1	2S	4.90	14.12	3.82
2	1D	15.89	12.36	7.06
3	1S	38.64	13.53	6.86
4	2D	38.25	40.40	0.20
5	2S	32.95	68.84	8.73
6	1D	62.96	67.47	13.14
7	1S	111.01	69.04	11.38
8	2D	103.95	121.21	15.30
9	2S	95.52	184.95	20.79
10	1D	152.59	179.27	24.32
11	1S	211.24	186.33	18.93
12	2D	195.74	252.23	23.05
13	2S	175.15	320.68	33.54
14	1D	263.21	318.91	37.76
15	2D	319.89	310.87	31.28

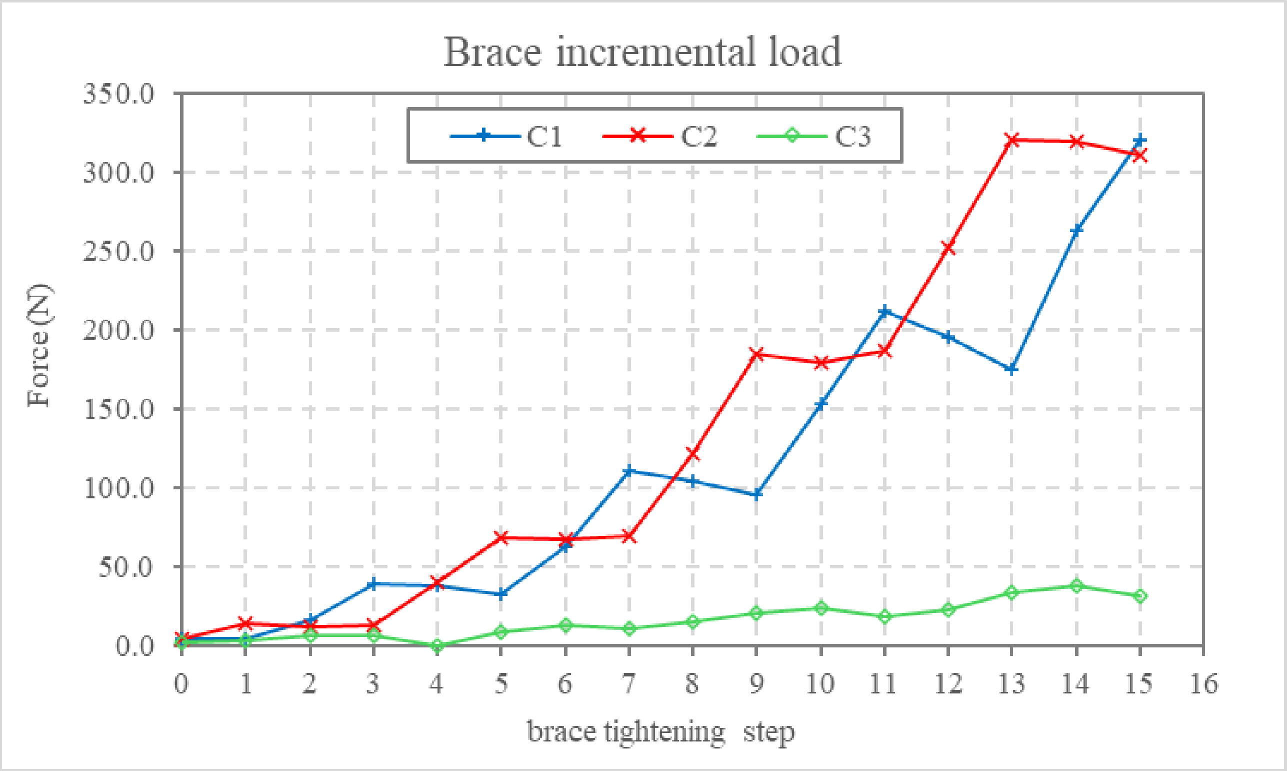

© MaCh3D s.r.l. Proprietary & Confidential

Tests without Spacers – CCC50-D0-LDB

LDB		Load cell		
		Bottom	Top	Mid
Step	Buckle Id	C1	C2	C3
--	--	*N*	*N*	*N*
0		4.71	7.45	3.43
1	2S	3.92	18.63	5.88
2	1D	9.02	16.28	8.73
3	1S	23.54	16.28	8.63
4	2D	26.09	44.52	0.00
5	2S	18.83	74.14	9.90
6	1D	41.19	79.24	12.16
7	1S	78.45	78.65	11.67
8	2D	71.00	131.61	13.73
9	2S	64.33	193.78	20.99
10	1D	105.72	188.88	26.58
11	1S	161.22	192.99	25.89
12	2D	153.57	275.57	16.87
13	2S	142.78	329.50	31.28
14	1D	207.12	339.51	39.23
15	2D	278.51	325.78	37.17

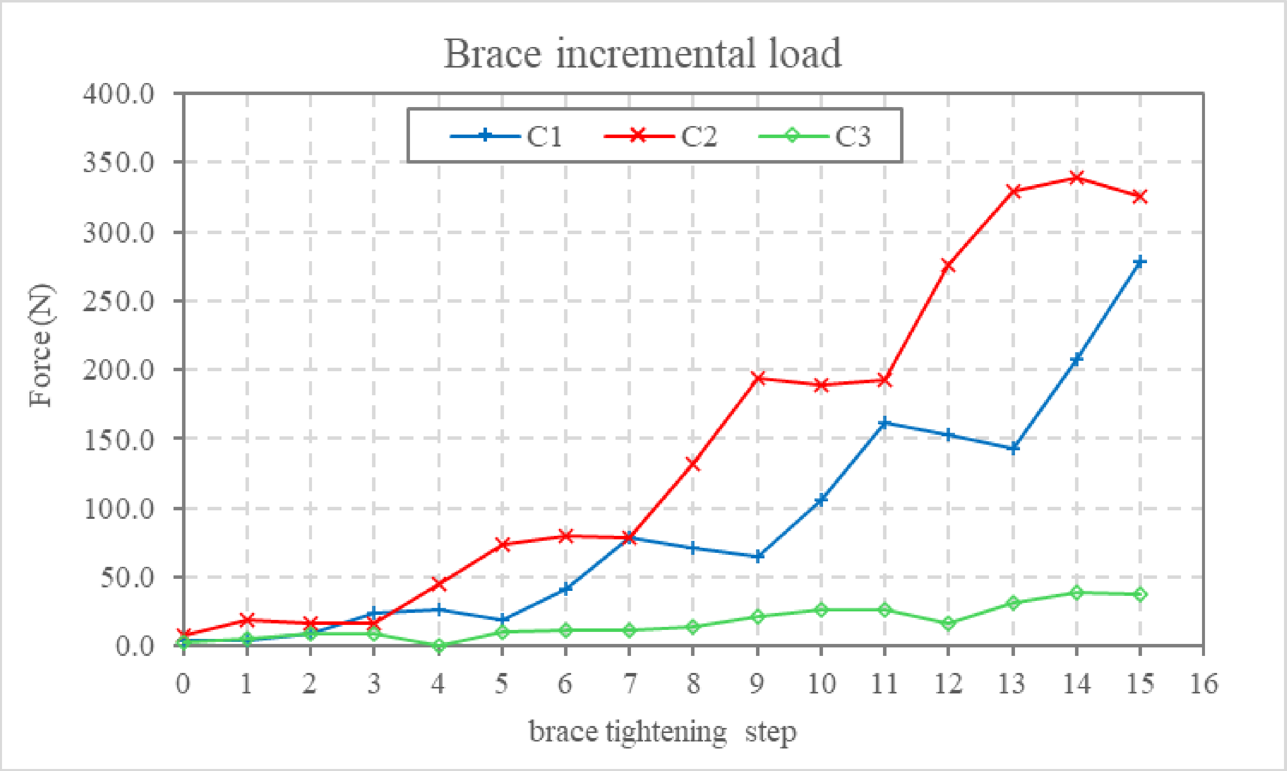

© MaCh3D s.r.l. Proprietary & Confidential

Tests without Spacers – CCC50-D0-LS

- Three tests were carried out using the same setup with single Laces (LS). This configuration allows the maximum magnitude of the force exerted by the brace on the torso under nominal alignment conditions to be evaluated.
- The forces measured present an oscillating trend like the previous test, resulting from the sequential execution of the clicks, performed one at a time. The Single Laces allow lower force levels to be achieved at the same number of clicks compared to Double Bonded laces (LDB), owing to its higher compliance.
- The mid load cell records lower force values compared to the other load cells. This behavior can be attributed to the absence of misalignment between the torso sections, confirming the correct functioning of the developed device.

LS		Load cell		
		Bottom	Top	Mid
Step	Buckle Id	C1	C2	C3
--	--	*N*	*N*	*N*
0	--	2.94	4.12	1.67
1	2S	3.14	10.79	3.43
2	1D	10.79	8.63	6.08
3	1S	28.05	9.81	5.49
4	2D	24.52	24.03	5.88
5	2S	22.95	46.09	11.18
6	1D	45.90	49.23	13.53
7	1S	85.12	55.70	6.67
8	2D	78.26	99.64	12.45
9	2S	70.80	154.95	16.87
10	1D	119.25	152.59	26.28
11	1S	174.36	158.08	24.32
12	2D	171.62	236.54	0.20
13	2S	157.10	279.88	24.22
14	1D	217.32	282.43	30.60
15	1S	268.31	280.67	28.05
16	2D	255.5613	335.3874	30.891
17	1D	324.60	331.46	35.01

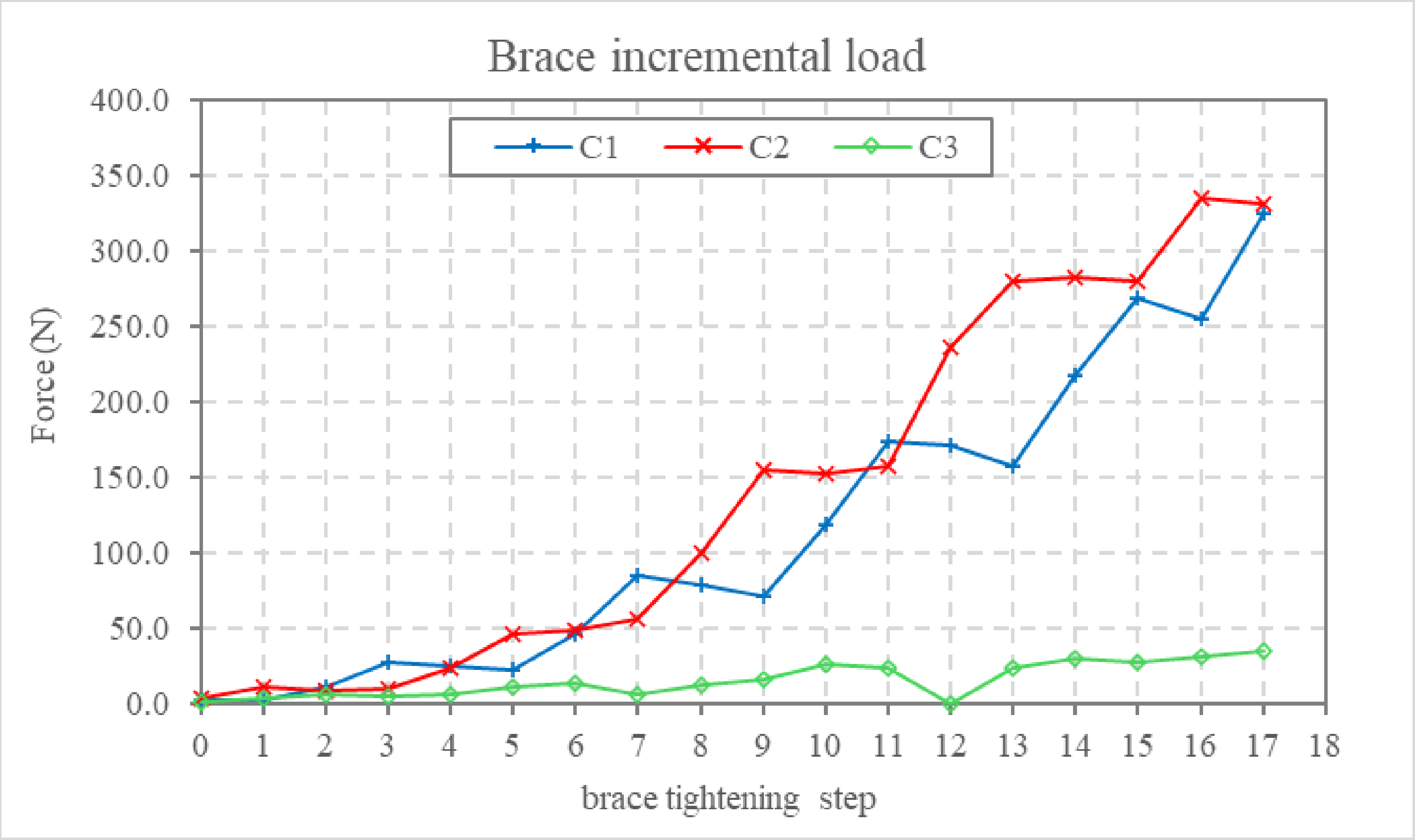

© MaCh3D s.r.l. Proprietary & Confidential

Tests without Spacers – CCC50-D0-LS

LS		Load cell		
		Bottom	Top	Mid
Step	Buckle Id	C1	C2	C3
--	--	*N*	*N*	*N*
0	--	2.75	2.94	1.77
1	2S	0.00	10.79	4.02
2	1D	6.28	9.02	5.88
3	1S	20.20	9.81	5.20
4	2D	16.87	20.40	6.18
5	2S	13.93	36.87	13.44
6	1D	34.72	35.50	12.16
7	1S	68.84	40.01	8.92
8	2D	61.39	80.81	15.89
9	2S	55.90	142.59	21.28
10	1D	93.36	133.57	24.32
11	1S	149.45	135.53	21.18
12	2D	147.10	225.75	0.00
13	2S	132.78	262.82	26.48
14	1D	191.43	267.13	35.30
15	1S	257.91	269.49	26.87
16	2D	246.54	331.46	32.85
17	1S	312.64	301.85	36.48

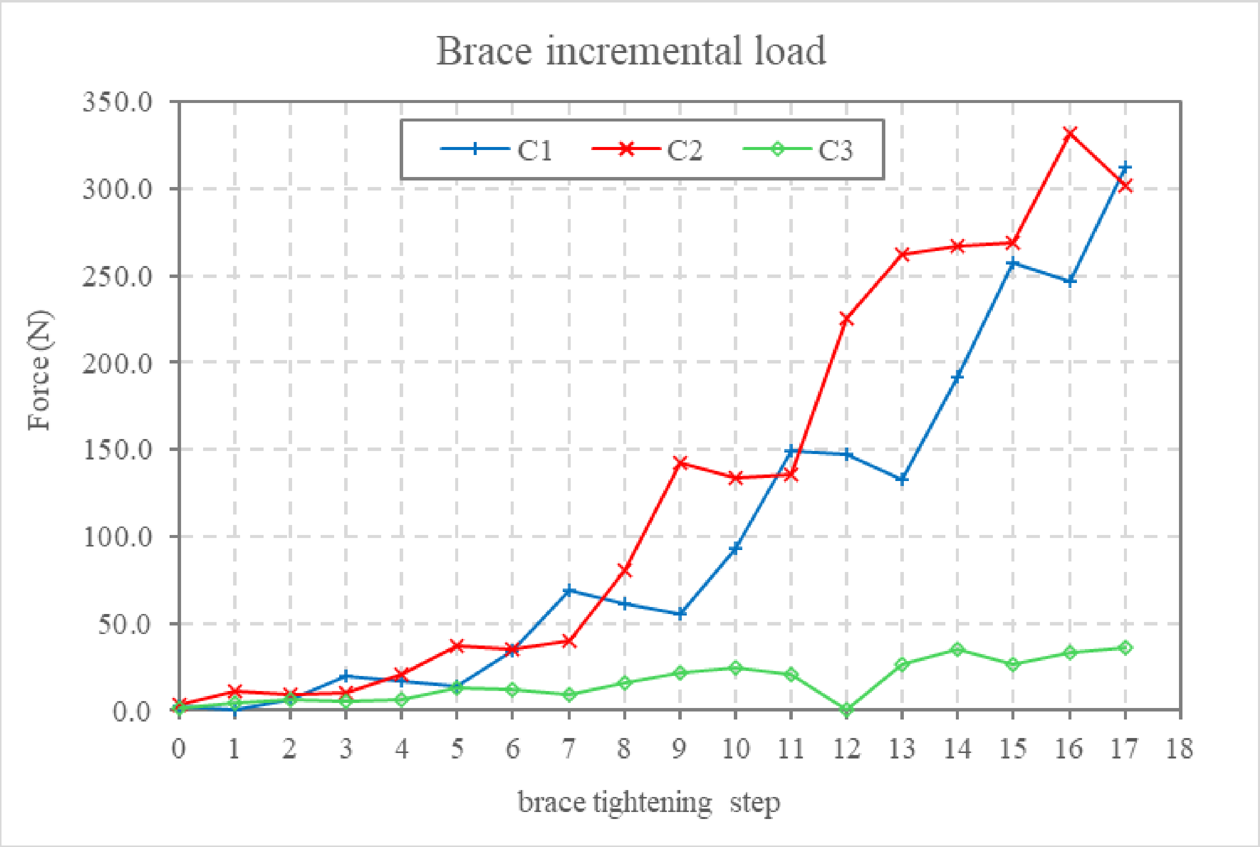

Tests without Spacers – CCC50-D0-LS

LS		Load cell		
		Bottom	Top	Mid
Step	Buckle Id	C1	C2	C3
--	--	N	N	N
0	--	2.75	0.00	0.88
1	2S	0.00	6.08	2.94
2	1D	4.31	4.31	5.39
3	1S	16.67	5.10	4.51
4	2D	17.46	16.87	0.00
5	2S	12.36	29.81	10.00
6	1D	29.03	30.60	14.32
7	1S	62.96	31.97	10.98
8	2D	52.96	66.29	14.81
9	2S	52.96	124.74	14.12
10	1D	89.24	120.62	18.24
11	1S	144.55	126.51	15.30
12	2D	139.25	199.47	0.00
13	2S	126.11	246.15	22.95
14	1D	188.29	253.01	32.07
15	1S	252.62	252.03	30.40
16	2D	245.36	326.95	31.38
17	2S	238.50	307.54	30.30
18	1S	293.22	301.46	33.34

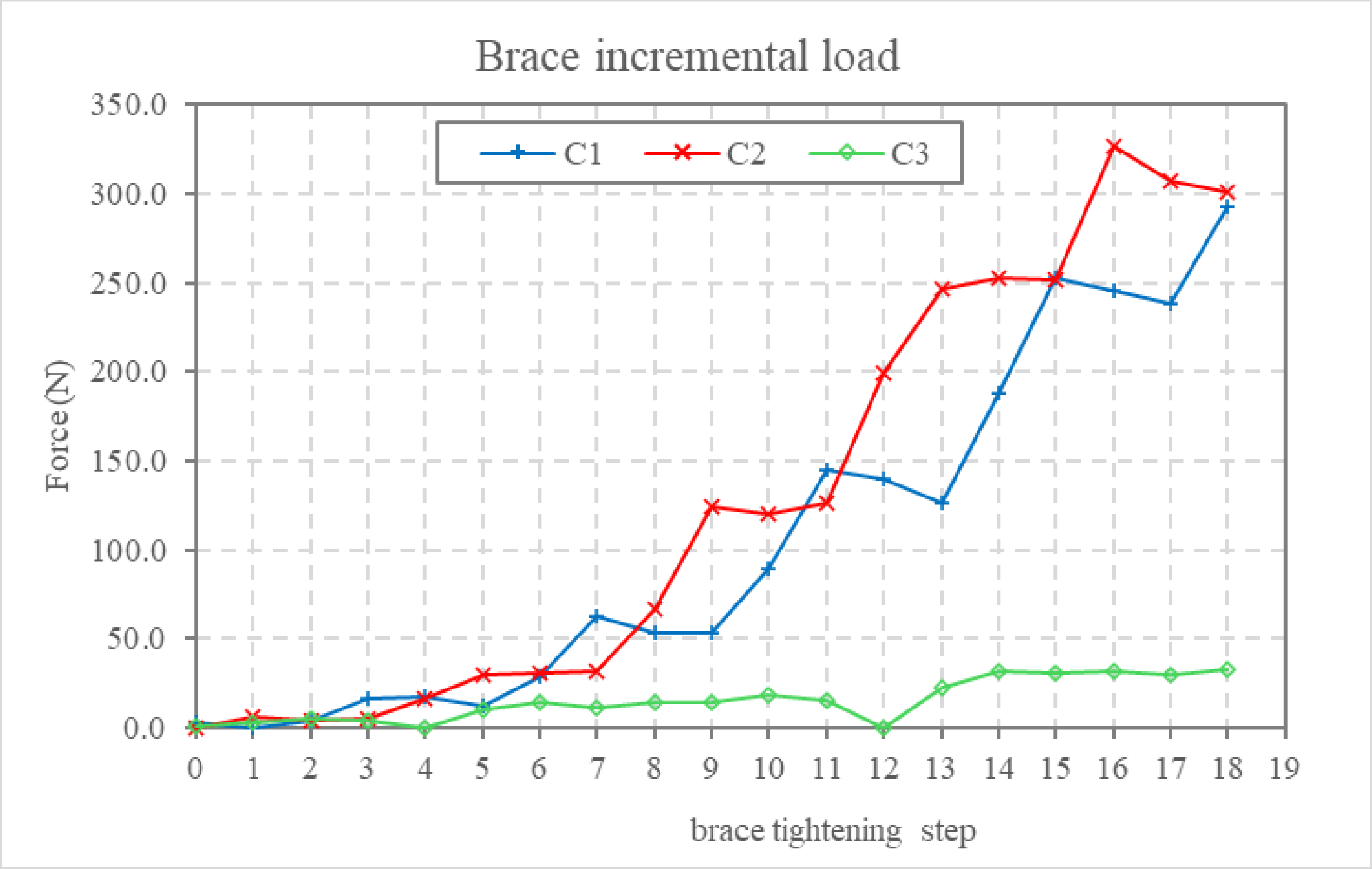

© MaCh3D s.r.l. Proprietary & Confidential

© MaCh3D s.r.l. Proprietary & Confidential

Tests with Spacers – CCC50-D4-LD

- Three tests were carried out using 3D-printed spacers with a thickness of 4 mm and Double Laces (LD).
- This setup allows for the evaluation of the maximun shear force that the brace can apply to the torso in the presence of a controlled and limited misalignment.
- By comparing the results with the previous diagrams, it becomes evident that, in this case, the shear force assumes a significantly higher value, confirming the influence of misalignment on the distribution of forces among the load cells.
- The trend is the same as in similar tests, but with higher load levels.

LD		Load cell		
		Bottom	Top	Mid
Step	Buckle Id	C1	C2	C3
--	--	*N*	*N*	*N*
0	--	0.00	0.00	12.65
1	2S	0.00	3.53	17.65
2	1D	2.55	2.35	25.60
3	1S	9.81	3.53	31.38
4	2D	7.45	11.18	35.40
5	2S	4.51	26.28	42.66
6	1D	10.79	28.24	51.68
7	1S	24.32	32.75	66.10
8	2D	21.77	57.47	76.30
9	2S	22.56	96.30	81.49
10	1D	41.58	92.18	102.68
11	1S	76.69	93.36	117.48
12	2D	72.37	138.47	118.76
13	2S	65.90	182.60	135.72
14	1D	96.50	191.82	154.65
15	2D	140.04	192.99	162.99
16	2D	130.23	242.42	181.32
17	2S	137.49	311.66	176.91
18	1D	187.31	296.75	200.25
19	1S	233.79	289.69	212.41

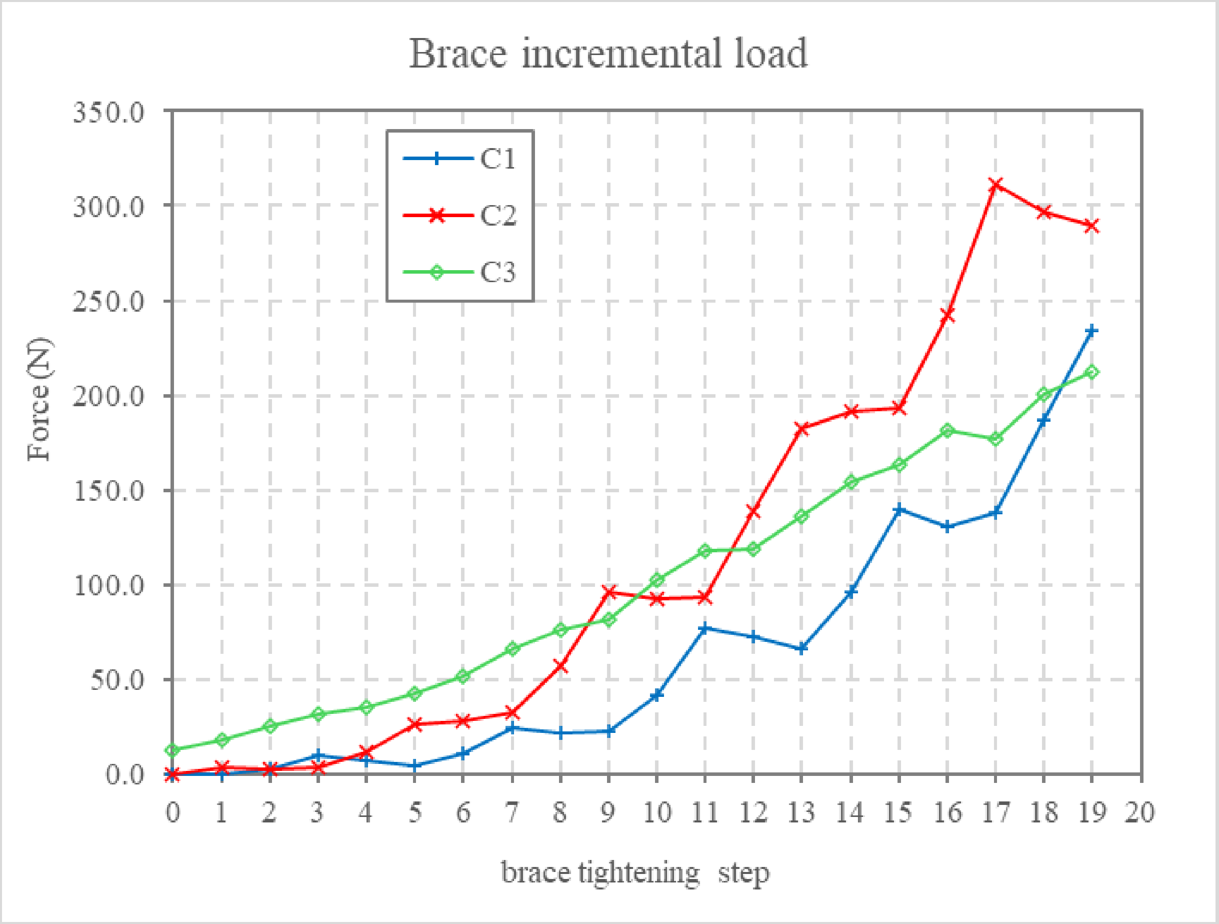

© MaCh3D s.r.l. Proprietary & Confidential

Tests without Spacers – CCC50-D4-LD

LD		Load cell		
		Bottom	Top	Mid
Step	Buckle Id	C1	C2	C3
--	--	*N*	*N*	*N*
0	--	0.00	0.00	5.10
1	2S	0.00	0.00	8.92
2	1D	0.00	0.00	14.91
3	1S	3.33	0.00	21.67
4	2D	2.75	0.00	27.65
5	2S	0.00	2.94	35.79
6	1D	8.24	4.31	44.72
7	1S	19.61	7.45	54.13
8	2D	17.06	16.08	66.19
9	2S	17.65	46.29	69.82
10	1D	38.25	39.03	90.42
11	1S	63.35	36.28	110.62
12	2D	60.21	77.28	112.09
13	2S	53.74	123.17	133.66
14	1D	91.20	123.17	148.86
15	1S	134.35	121.01	159.85
16	2D	126.90	168.67	178.19
17	2S	124.94	234.38	181.52
18	1D	184.37	225.75	202.90
19	1S	216.53	227.51	206.43
20	2D	211.43	286.75	212.41

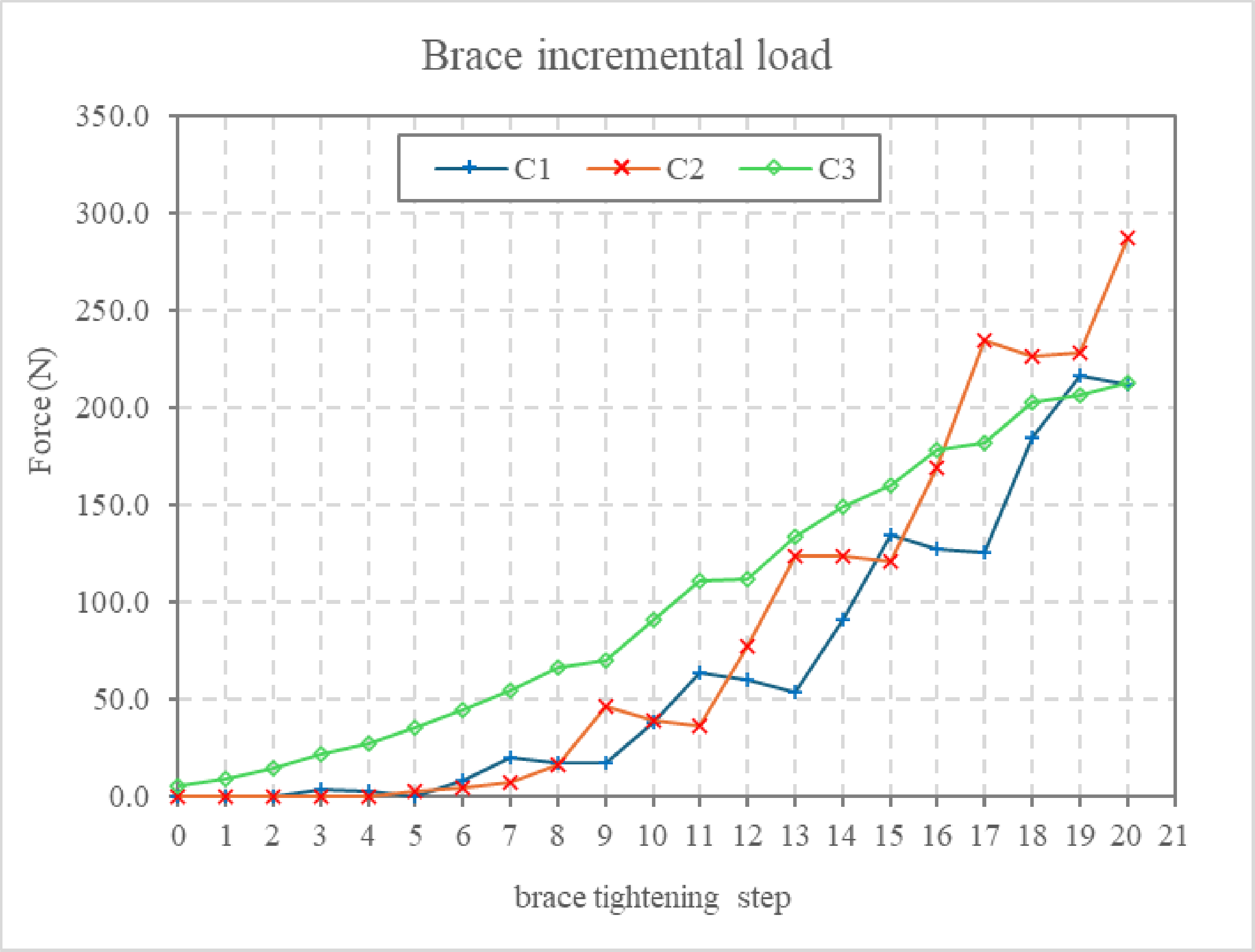

© MaCh3D s.r.l. Proprietary & Confidential

Tests without Spacers – CCC50-D4-LD

LD		Load cell		
		Bottom	Top	Mid
Step	Buckle Id	C1	C2	C3
--	--	*N*	*N*	*N*
0	--	0.00	0.00	4.51
1	2S	0.00	0.00	5.98
2	1D	2.55	0.00	9.81
3	1S	6.86	0.00	15.59
4	2D	4.71	0.00	21.08
5	2S	4.51	0.00	28.24
6	1D	11.77	0.00	35.30
7	1S	24.52	0.00	41.97
8	2D	22.56	3.33	52.96
9	2S	17.46	15.30	62.86
10	1D	41.38	10.40	79.14
11	1S	73.75	11.38	94.14
12	2D	70.80	39.03	96.99
13	2S	64.33	69.63	114.74
14	1D	102.97	71.00	129.64
15	1S	152.20	69.04	145.73
16	2D	141.80	112.78	162.30
17	2S	138.08	176.72	166.52
18	1D	183.38	164.95	188.68
19	1S	237.52	162.99	198.49
20	2D	222.81	217.12	214.28
21	2S	216.33	277.72	211.33

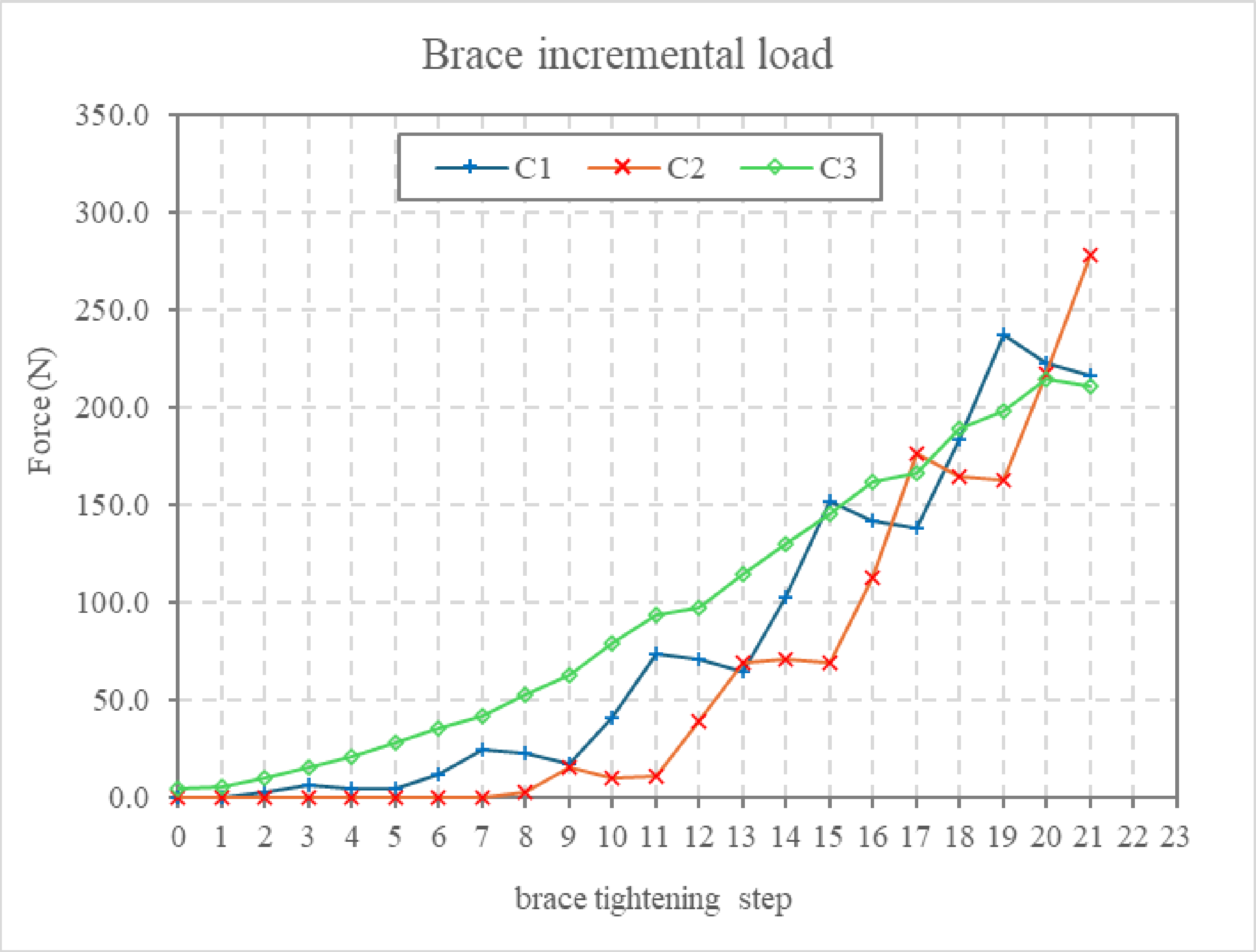

© MaCh3D s.r.l. Proprietary & Confidential

Tests without Spacers – CCC50-D2-LD

- Three tests were carried out using 3D-printed spacers with a thickness of 2 mm and Double Laces (LD).
- This setup allows for the evaluation of the maximun shear force that the brace can apply to the torso in the presence of a controlled and limited misalignment.
- By comparing the results with the previous diagrams, it becomes evident that, in this case, the shear force assumes a significantly higher value, confirming the influence of misalignment on the distribution of forces among the load cells.

LD		Load cell		
		Bottom	Top	Mid
Step	Buckle Id	C1	C2	C3
--	--	*N*	*N*	*N*
0	--	2.55	0.00	11.96
1	2S	0.00	3.53	14.81
2	1D	9.22	0.00	20.30
3	1S	22.75	0.00	25.11
4	2D	20.20	11.38	29.22
5	2S	15.89	26.09	38.25
6	1D	34.72	27.85	47.37
7	1S	65.12	31.38	54.92
8	2D	58.64	54.13	66.39
9	2S	56.09	100.22	72.08
10	1D	95.91	95.32	91.59
11	1S	144.35	96.30	101.50
12	2D	138.47	149.45	101.30
13	2S	127.29	194.37	114.93
14	1D	185.15	197.31	121.21
15	2D	231.44	188.88	127.29
16	2D	220.85	257.91	136.12
17	2S	215.55	312.44	131.70
18	1D	270.08	297.34	146.90
19	1S	241.44	371.67	141.71

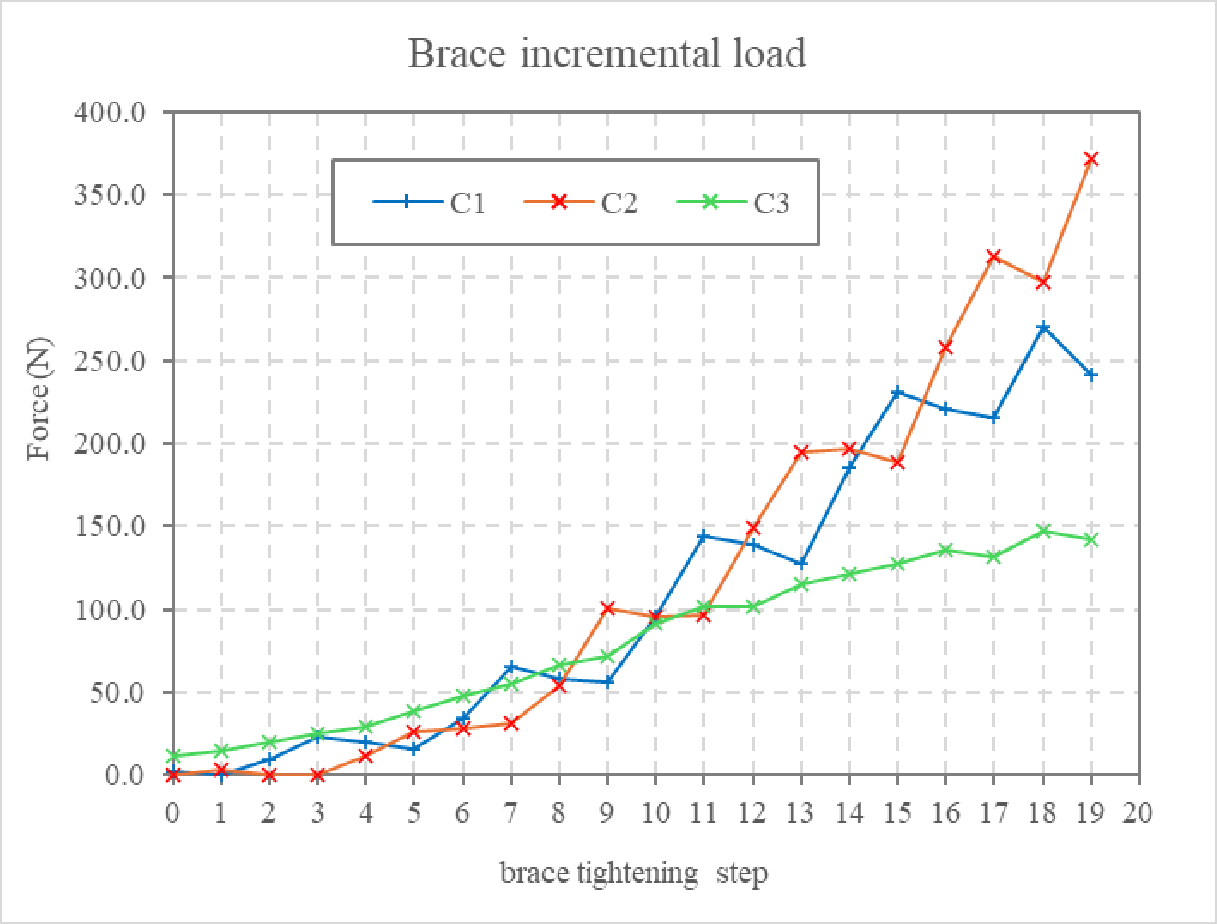

© MaCh3D s.r.l. Proprietary & Confidential

Tests without Spacers – CCC50-D2-LD

LD		Load cell		
		Bottom	Top	Mid
Step	Buckle Id	C1	C2	C3
--	--	*N*	*N*	*N*
0	--	0.00	0.00	6.28
1	2S	0.00	3.33	7.45
2	1D	5.69	0.00	12.55
3	1S	15.89	0.00	16.28
4	2D	13.14	6.08	20.50
5	2S	10.79	18.24	26.48
6	1D	26.87	19.42	31.77
7	1S	51.39	19.81	38.64
8	2D	46.48	41.38	47.56
9	2S	45.31	81.59	49.92
10	1D	81.59	72.77	65.90
11	1S	127.88	74.14	78.45
12	2D	121.60	129.64	81.69
13	2S	109.83	174.36	85.61
14	1D	170.64	180.83	100.52
15	2D	236.54	181.62	103.46
16	2D	221.63	255.95	110.82
17	2S	211.04	312.05	112.68
18	1D	274.78	289.79	130.33
19	1S	268.31	374.61	126.11

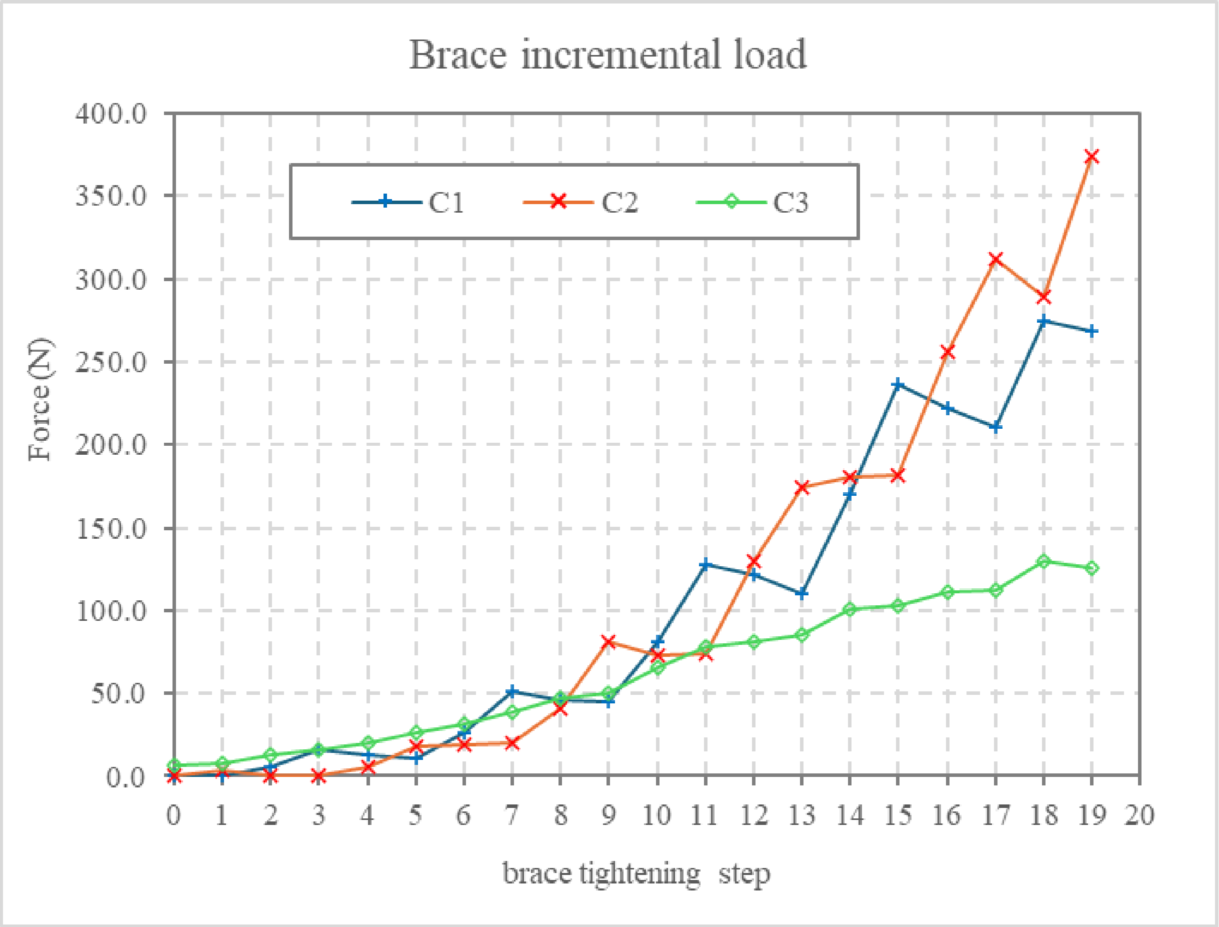

© MaCh3D s.r.l. Proprietary & Confidential

Tests without Spacers – CCC50-D2-LD

LD		Load cell		
		Bottom	Top	Mid
Step	Buckle Id	C1	C2	C3
--	--	*N*	*N*	*N*
0	--	0.00	0.00	4.81
1	2S	0.00	0.00	7.65
2	1D	2.16	0.00	12.36
3	1S	10.40	0.00	15.20
4	2D	8.43	2.75	20.89
5	2S	7.06	11.38	26.48
6	1D	19.22	12.36	32.36
7	1S	40.01	13.34	38.74
8	2D	37.85	31.38	47.95
9	2S	33.93	64.92	56.88
10	1D	64.72	60.02	72.77
11	1S	108.85	58.06	80.51
12	2D	104.34	113.36	84.83
13	2S	95.52	157.89	101.01
14	1D	152.20	159.06	113.66
15	2D	208.10	155.14	116.80
16	2D	189.27	211.04	127.58
17	2S	186.52	276.74	127.68
18	1D	249.09	261.25	141.61
19	2D	234.38	329.11	139.35
20	2S	220.85	372.85	148.47

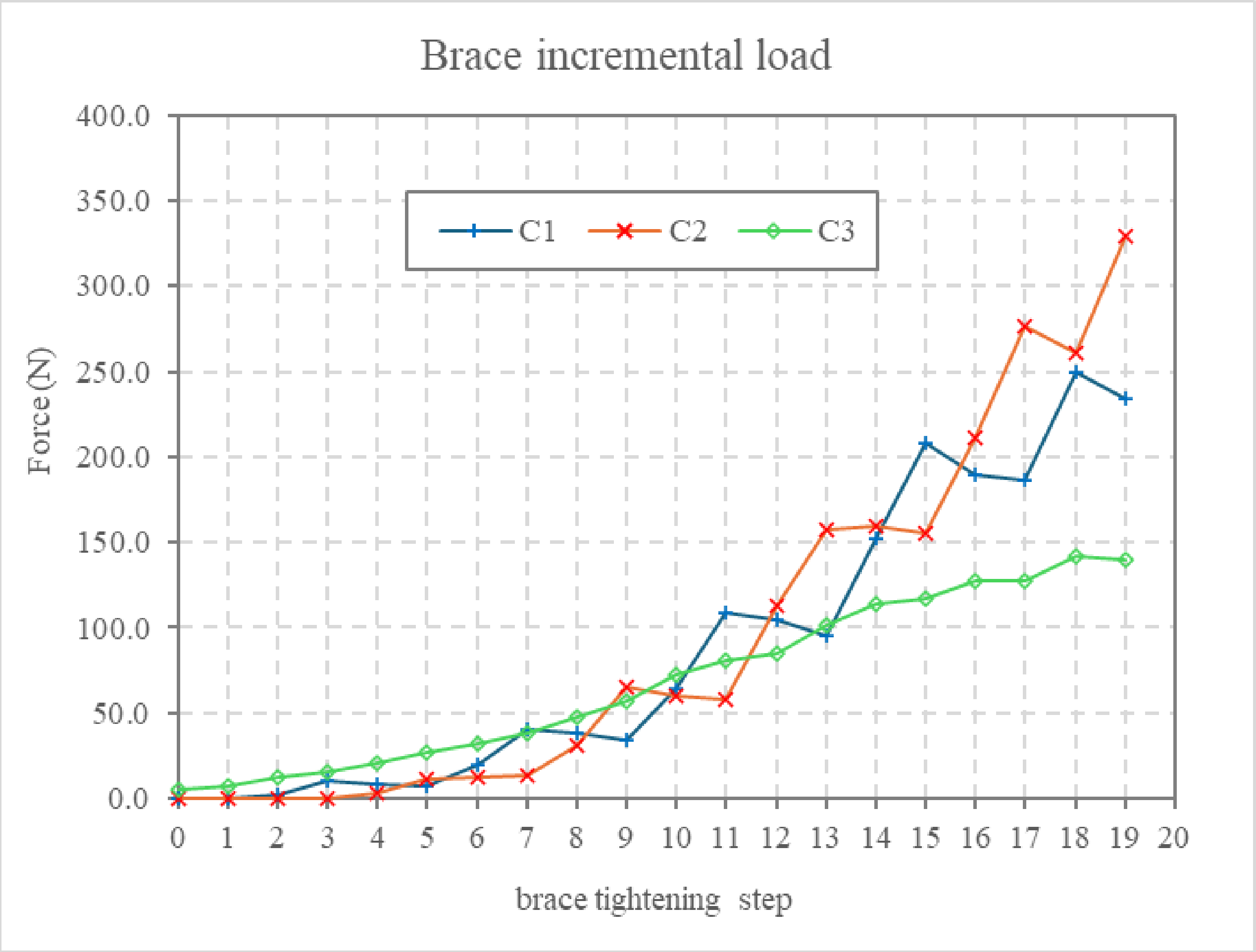

© MaCh3D s.r.l. Proprietary & Confidential

Tests without Spacers – CCC50-D2-LDB

- Three tests were carried out using 3D-printed spacers with a thickness of 2 mm and Double Bonded Laces (LDB).
- This setup allows for the evaluation of the maximun shear force that the brace can apply to the torso in the presence of a controlled and limited misalignment.
- By comparing the results with the previous diagrams, it becomes evident that, in this case, the shear force assumes a significantly higher value, confirming the influence of misalignment on the distribution of forces among the load cells.

LDB		Load cell		
		Bottom	Top	Mid
Step	Buckle Id	C1	C2	C3
--	--	*N*	*N*	*N*
0	--	4.71	0.00	6.86
1	2S	0.00	4.90	13.83
2	1D	6.08	2.55	21.87
3	1S	18.04	4.71	24.81
4	2D	16.08	14.51	27.75
5	2S	11.77	26.67	38.34
6	1D	27.65	27.46	49.33
7	1S	55.90	30.01	55.60
8	2D	45.90	59.04	68.65
9	2S	45.70	113.36	74.82
10	1D	83.16	100.42	93.46
11	1S	132.59	104.15	101.60
12	2D	123.17	157.69	104.15
13	2S	111.60	212.80	120.43
14	1D	166.12	212.41	128.57
15	1S	223.98	208.69	128.96
16	2D	211.0391	280.4702	138.86

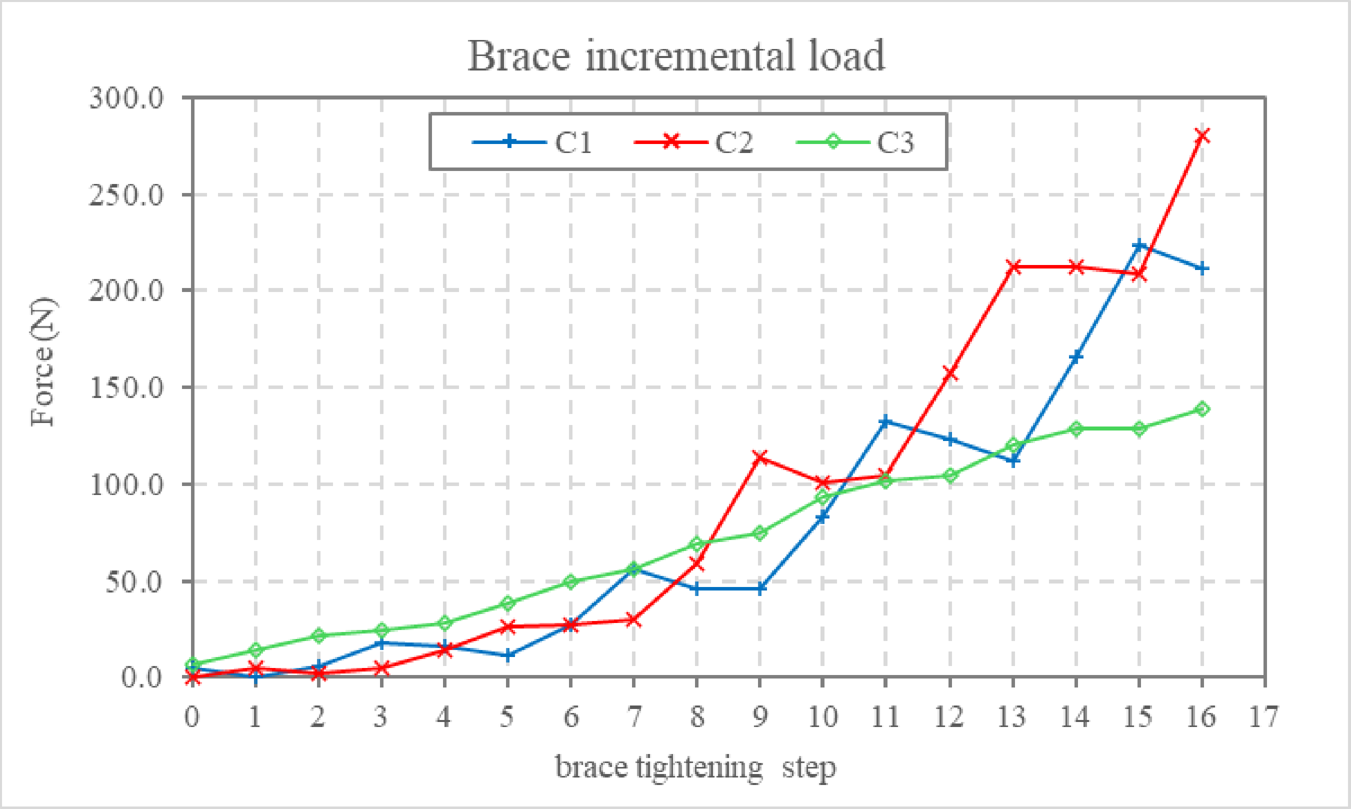

© MaCh3D s.r.l. Proprietary & Confidential

Tests without Spacers – CCC50-D2-LDB

LDB		Load cell		
		Bottom	Top	Mid
Step	Buckle Id	C1	C2	C3
--	--	*N*	*N*	*N*
0	--	0.00	0.00	5.98
1	2S	0.00	0.00	10.89
2	1D	6.28	0.00	13.34
3	1S	18.83	0.00	15.98
4	2D	16.28	8.24	18.93
5	2S	12.36	22.75	28.54
6	1D	27.07	22.95	37.56
7	1S	53.54	23.73	43.54
8	2D	45.90	55.11	56.39
9	2S	41.78	101.40	63.15
10	1D	80.22	93.36	82.08
11	1S	131.02	93.95	93.56
12	2D	119.84	158.08	94.83
13	2S	110.03	206.14	110.23
14	1D	163.28	207.90	119.84
15	1S	225.75	210.35	122.78
16	2D	219.08	281.647	126.90

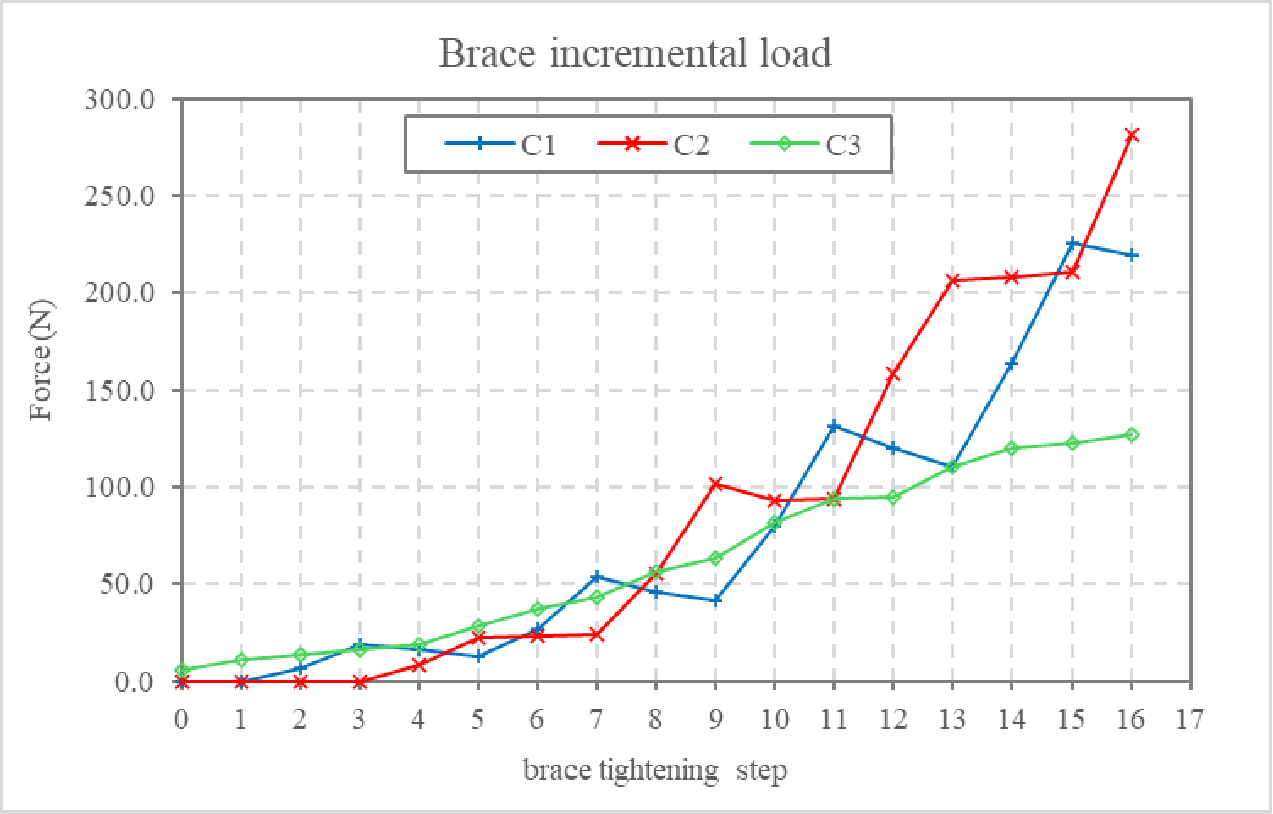

© MaCh3D s.r.l. Proprietary & Confidential

Tests without Spacers – CCC50-D2-LDB

LDB		Load cell		
		Bottom	Top	Mid
Step	Buckle Id	C1	C2	C3
--	--	*N*	*N*	*N*
0	--	2.55	0.00	5.69
1	2S	0.00	0.00	9.12
2	1D	4.31	0.00	14.81
3	1S	15.10	0.00	18.63
4	2D	11.77	4.31	23.05
5	2S	9.22	17.06	30.30
6	1D	23.73	16.87	38.64
7	1S	50.01	18.24	44.82
8	2D	44.13	45.11	56.29
9	2S	43.35	98.26	57.86
10	1D	78.85	88.06	79.83
11	1S	134.55	87.67	90.52
12	2D	123.76	147.30	93.85
13	2S	110.23	204.57	108.85
14	1D	165.73	198.88	122.68
15	1S	230.65	194.56	121.11
16	2D	216.727	268.7022	130.0362

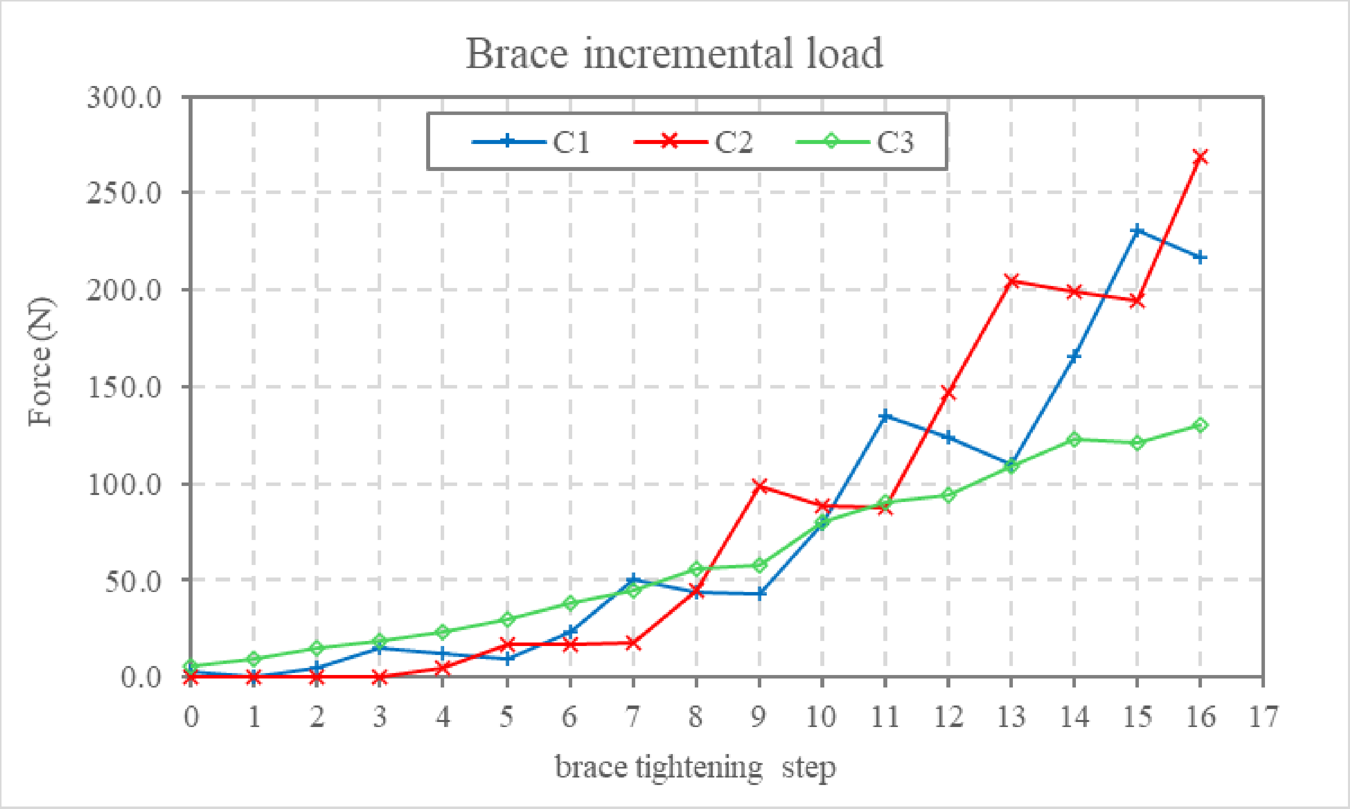

© MaCh3D s.r.l. Proprietary & Confidential

Tests without Spacers – CCC50-D4-LDB

- Three tests were carried out using 3D-printed spacers with a thickness of 4 mm and Double Bonded Laces (LDB).
- This setup allows for the evaluation of the maximun shear force that the brace can apply to the torso in the presence of a controlled and limited misalignment.
- By comparing the results with the previous diagrams, it becomes evident that, in this case, the shear force assumes a significantly higher value, confirming the influence of misalignment on the distribution of forces among the load cells.

LDB		Load cell		
		Bottom	Top	Mid
Step	Buckle Id	C1	C2	C3
--	--	*N*	*N*	*N*
0	--	0.00	0.00	13.04
1	2S	0.00	3.33	18.53
2	1D	2.16	0.00	25.30
3	1S	8.63	0.00	31.77
4	2D	6.08	6.08	35.89
5	2S	4.71	15.69	47.66
6	1D	14.32	17.85	60.02
7	1S	34.13	19.02	67.37
8	2D	27.85	37.27	80.81
9	2S	27.07	82.77	84.24
10	1D	56.88	72.77	112.97
11	1S	98.26	70.02	131.61
12	2D	93.16	130.43	134.84
13	2S	81.98	167.69	157.89
14	1D	122.98	172.20	180.05
15	2D	171.62	172.40	187.99
16	2D	161.81	234.58	206.14
17	2S	148.47	275.57	216.24
18	1D	206.14	268.70	225.55
19	1S	266.15	272.23	233.01

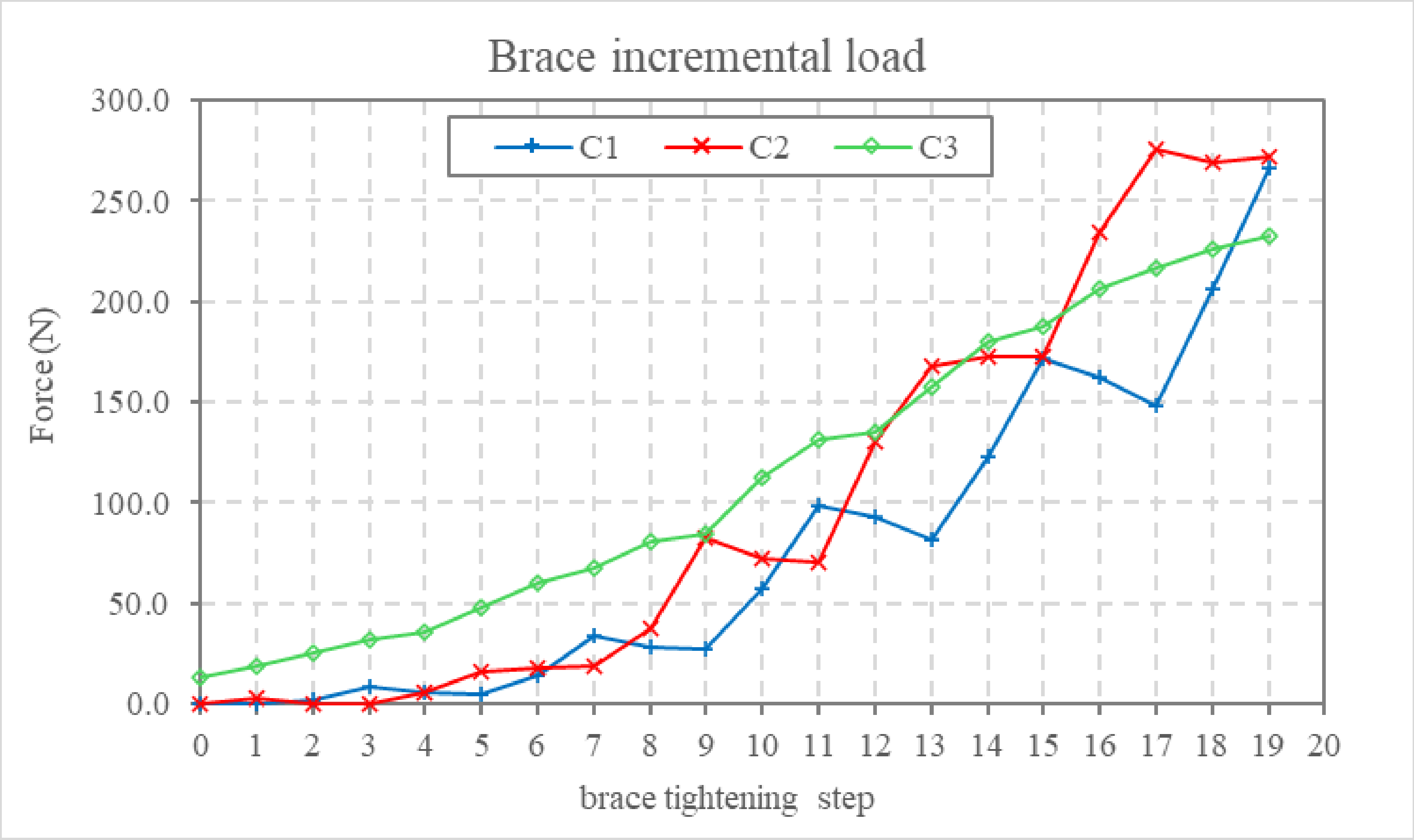

© MaCh3D s.r.l. Proprietary & Confidential

Tests without Spacers – CCC50-D4-LDB

LDB		Load cell		
		Bottom	Top	Mid
Step	Buckle Id	C1	C2	C3
--	--	*N*	*N*	*N*
0	--	0.00	0.00	7.16
1	2S	0.00	0.00	11.87
2	1D	2.55	0.00	16.18
3	1S	6.08	0.00	23.24
4	2D	4.12	3.53	28.83
5	2S	0.00	7.06	36.58
6	1D	7.06	8.43	46.68
7	1S	21.18	9.02	58.35
8	2D	17.65	22.56	67.57
9	2S	15.30	47.27	74.14
10	1D	40.40	42.56	94.34
11	1S	76.49	44.33	110.13
12	2D	71.00	88.65	114.54
13	2S	61.78	125.13	133.96
14	1D	100.22	130.43	155.93
15	1S	152.00	130.23	162.99
16	2D	140.43	196.33	182.70
17	2S	117.88	230.46	178.68
18	1D	181.23	235.75	196.33
19	1S	239.28	232.42	204.27
20	2D	217.71	290.28	222.12

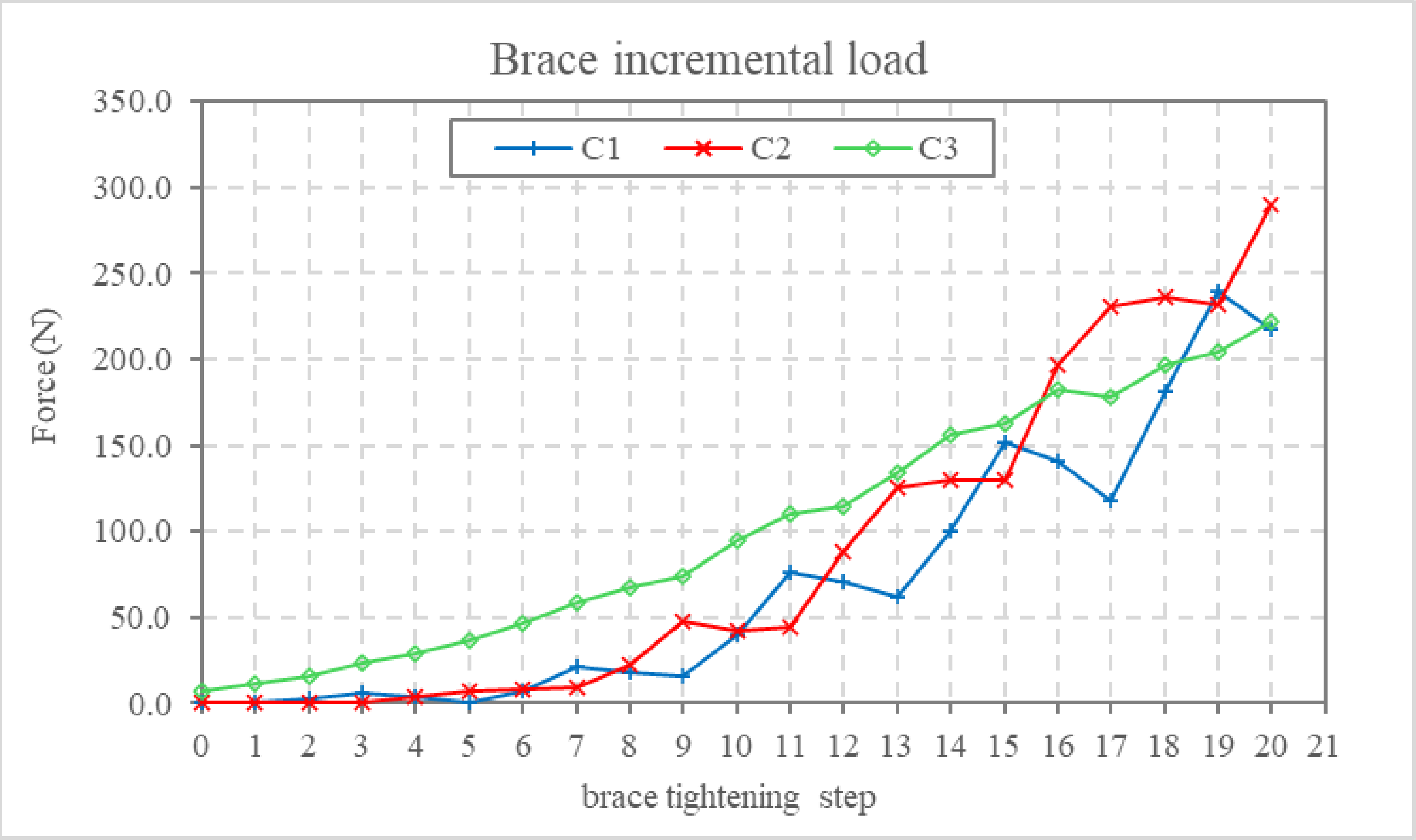

© MaCh3D s.r.l. Proprietary & Confidential

Tests without Spacers – CCC50-D4-LDB

LDB		Load cell		
		Bottom	Top	Mid
Step	Buckle Id	C1	C2	C3
--	--	*N*	*N*	*N*
0	--	0.00	0.00	7.55
1	2S	0.00	0.00	12.65
2	1D	3.53	0.00	20.40
3	1S	11.96	0.00	26.67
4	2D	9.02	2.75	31.97
5	2S	5.88	9.02	43.35
6	1D	20.59	10.20	51.48
7	1S	39.42	11.18	62.57
8	2D	34.91	26.48	74.53
9	2S	35.11	58.45	78.16
10	1D	65.12	50.60	100.22
11	1S	108.07	49.62	115.23
12	2D	100.22	96.11	118.76
13	2S	93.16	142.39	140.33
14	1D	137.49	139.45	156.51
15	1S	188.48	140.04	165.34
16	2D	179.27	199.07	184.27
17	2S	169.66	233.69	189.07
18	1D	223.40	224.57	201.72
19	1S	264.98	227.12	206.43
20	2D	262.82	260.46	212.80

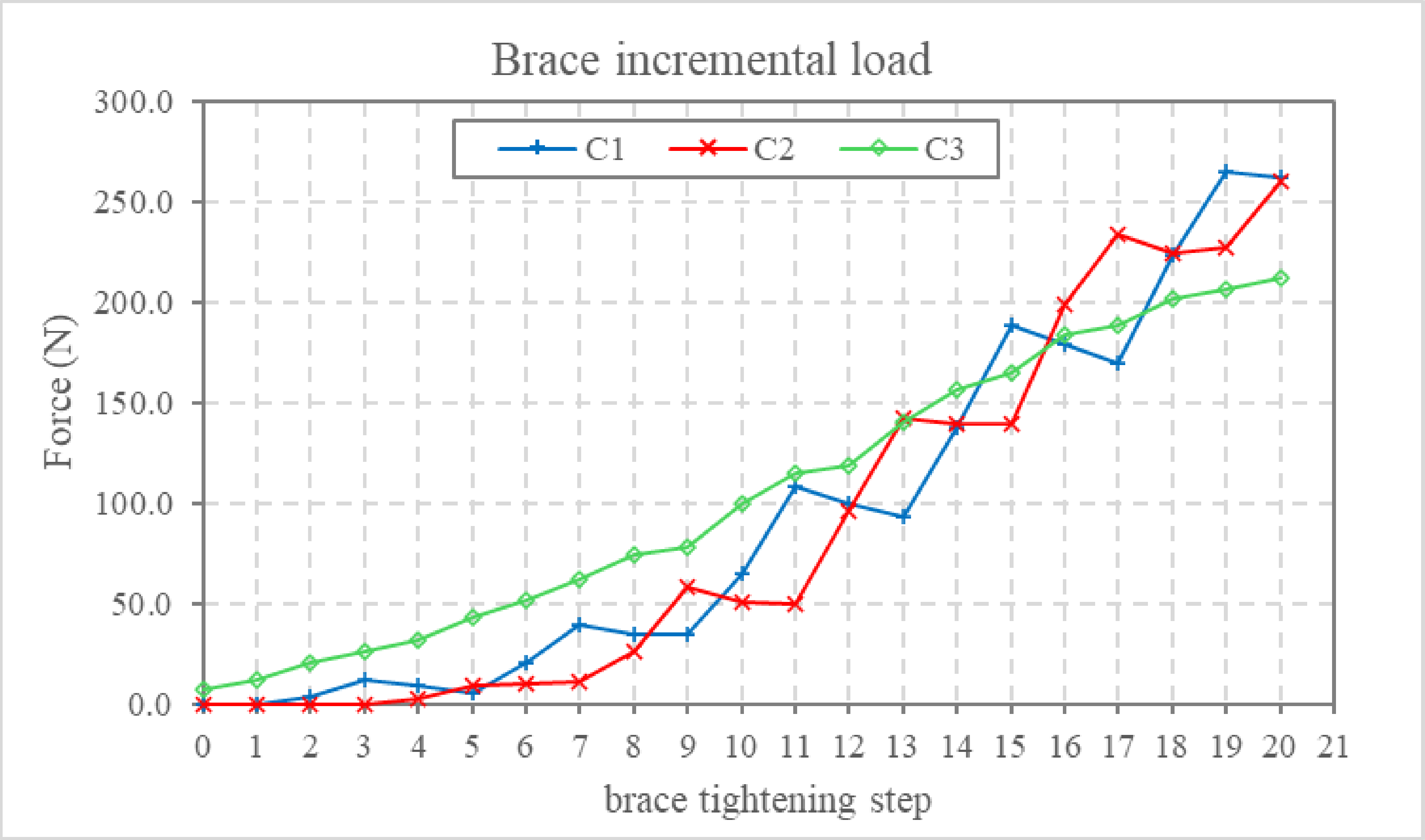

© MaCh3D s.r.l. Proprietary & Confidential

Tests without Spacers – CCC50-D0-NL

NL		Load cell		
		Bottom	Top	Mid
Step	Buckle Id	C1	C2	C3
--	--	N	N	N
0	--	10.40	3.33	0.78
1	2S	10.59	10.20	0.69
2	1D	20.59	9.22	2.94
3	1S	33.93	11.77	0.10
4	2D	31.77	23.93	0.00
5	2S	32.75	33.15	1.77
6	1D	49.23	34.52	3.63
7	1S	65.90	33.54	2.35
8	2D	64.14	46.48	4.71
9	2S	65.90	74.73	1.47
10	1D	91.40	76.88	1.77
11	1S	123.56	79.43	3.43
12	2D	116.70	99.83	0.00
13	2S	117.29	109.83	0.00
14	1D	149.85	115.91	0.00
15	1S	158.67	126.70	2.65
16	2D	157.10	129.45	5.30
17	2S	154.36	149.65	1.57
18	1D	197.31	164.75	0.69
19	1S	215.94	153.18	9.41
20	2D	213.20	183.09	0.00
21	2S	214.18	176.72	0.59
22	1D	240.26	189.27	1.67
23	1S	248.11	192.41	11.18
24	2D	246.54	206.92	14.81
25	2S	246.93	233.89	4.41
26	1D	285.37	241.83	0.98
27	1S	305.38	235.75	12.75
28	2D	308.32	257.91	0.00
29	2S	300.08	264.39	8.83

- Test was carried out with the brace straps removed in order to assess their influence on the forces measured by the load cells.
- Lower force levels are achieved for the same number of clicks due to due to the increased deformation of the neoprene.

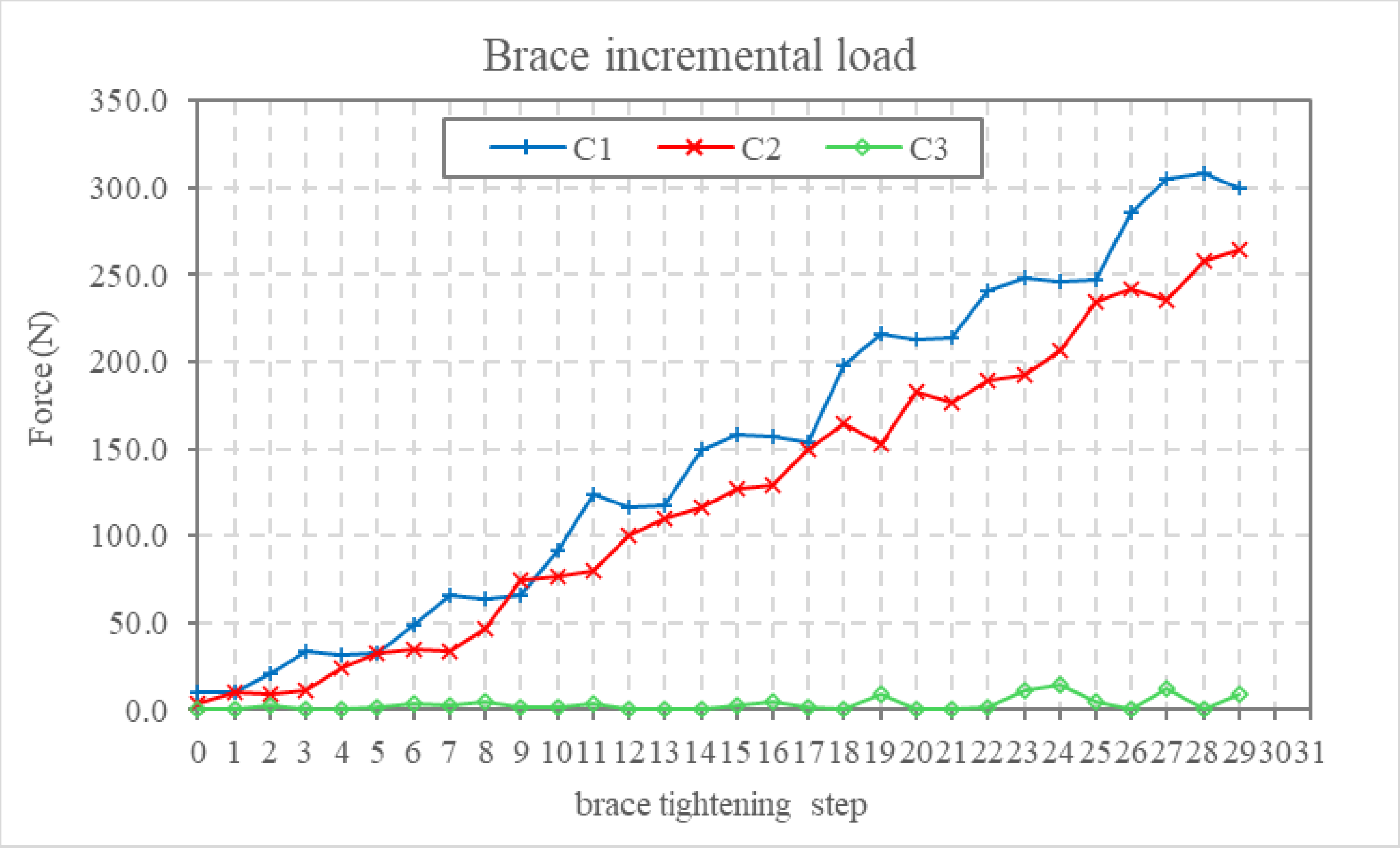

© MaCh3D s.r.l. Proprietary & Confidential

Experimental Test for FE Calibration

Tests on Low Racks-Only Actuation – CTC20-D0

- Tests were conducted to calibrate the FEM model.
  Only the lower racks was actuated, applying alternating clicks.
- Actuation of the lower latches alone generates an increase in force only in the lower part of the brace.

LD		Load cell	
		Bottom	Top
Step	Buckle Id	C1	C2
--	--	*N*	*N*
0	--	4.707	0.000
1	1D	16.671	0.000
2	1S	38.638	0.000
3	1D	68.450	0.000
4	1S	103.950	2.746
5	1D	143.177	4.315

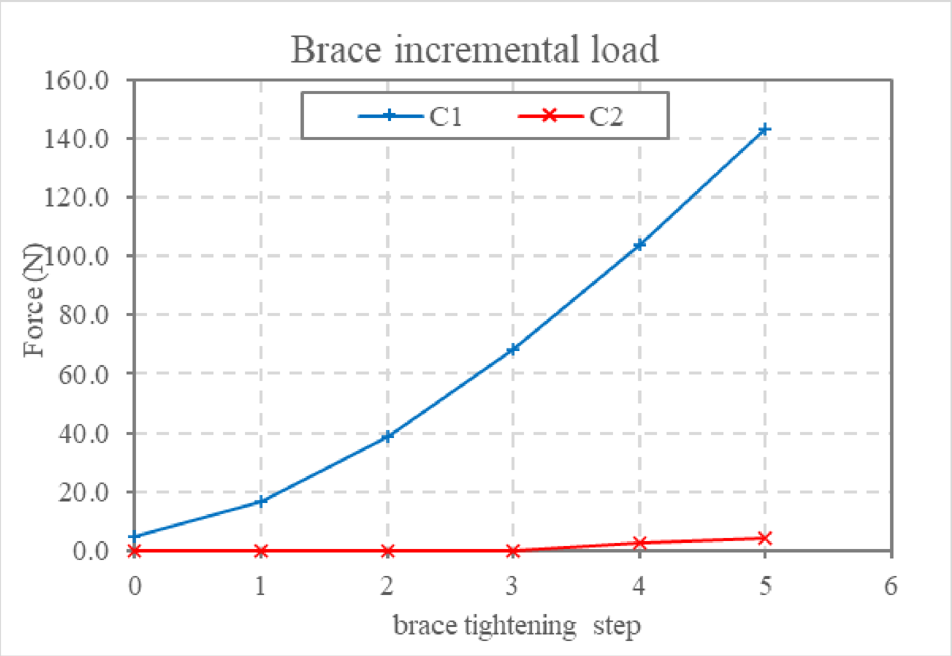

© MaCh3D s.r.l. Proprietary & Confidential

© MaCh3D s.r.l. Proprietary & Confidential

Tests on Low Rack-Only Actuation – CTC20-D0

LD		Load cell	
		Bottom	Top
Step	Buckle Id	C1	C2
--	--	*N*	*N*
0	--	3.923	0.000
1	1D	13.337	0.000
2	1S	33.343	0.000
3	1D	60.017	0.000
4	1S	94.144	0.000
5	1D	142.785	1.961

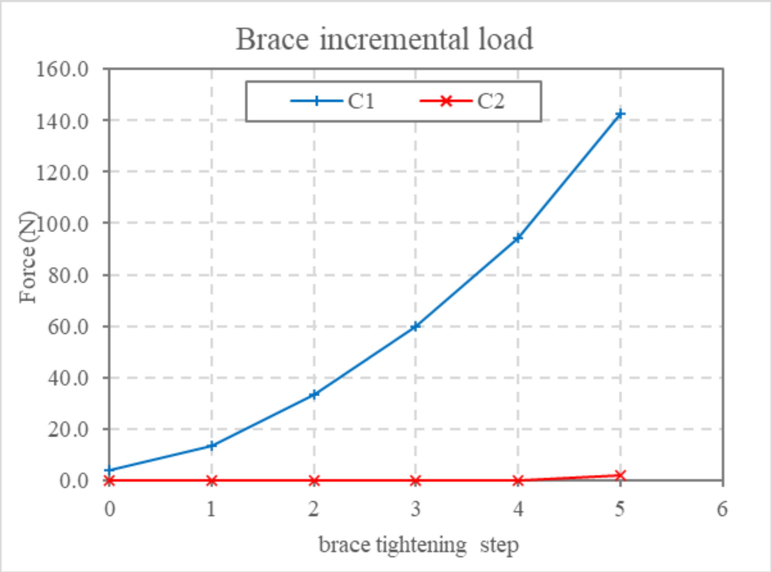

LD		Load cell	
		Bottom	Top
Step	Buckle Id	C1	C2
--	--	*N*	*N*
0	--	3.727	0.000
1	1D	12.749	0.000
2	1S	30.989	0.000
3	1D	56.486	0.000
4	1S	93.359	0.000
5	1D	140.235	2.354

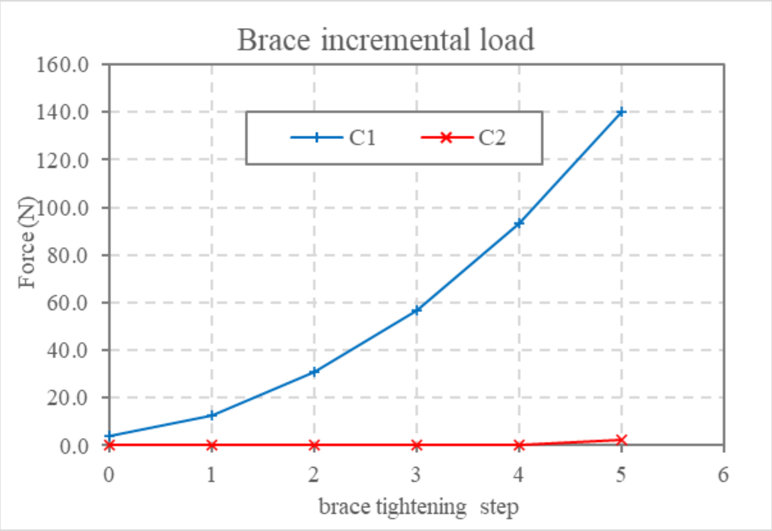

© MaCh3D s.r.l. Proprietary & Confidential

Tests on Upper Racks-Only Actuation – CTC20-D0

- Tests were conducted to calibrate the FEM model.
  Only the upper racks was actuated, applying alternating clicks.
- Actuation of the upper latches alone generates an increase in force only in the upper part of the brace.

LD		Load cell	
		Bottom	Top
Step	Buckle Id	C1	C2
--	--	*N*	*N*
0	--	7.257	3.334
1	2S	9.022	12.356
2	2D	8.826	32.166
3	2S	8.826	69.235
4	2D	10.395	115.915
5	2S	10.983	163.379

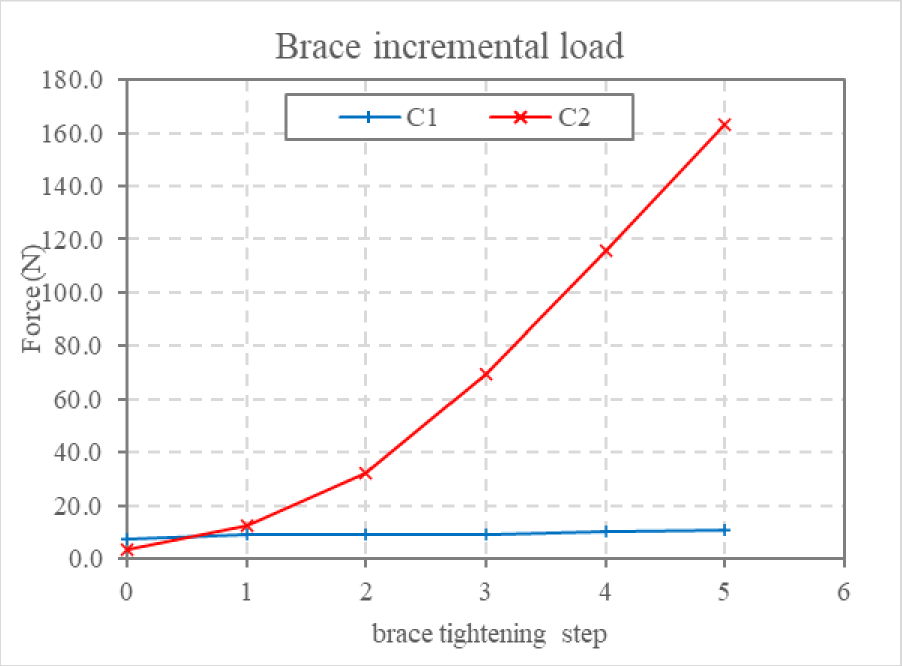

© MaCh3D s.r.l. Proprietary & Confidential

Tests on Low Rack-Only Actuation – CTC20-D0

LD		Load cell	
		Bottom	Top
Step	Buckle Id	C1	C2
--	--	*N*	*N*
0	--	9.218	0.000
1	2S	9.218	6.276
2	2D	8.826	22.948
3	2S	9.022	57.075
4	2D	9.218	101.499
5	2S	10.003	157.103

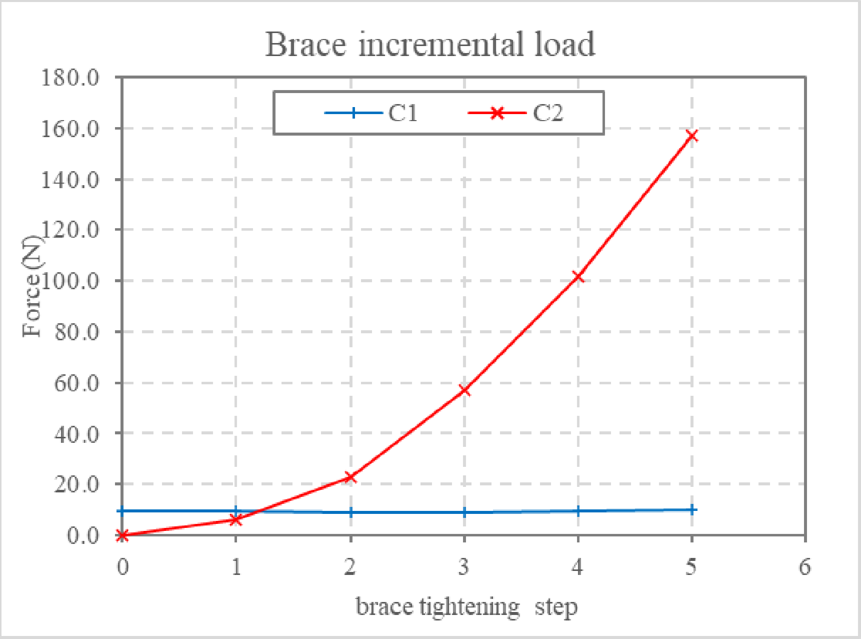

LD		Load cell	
		Bottom	Top
Step	Buckle Id	C1	C2
--	--	*N*	*N*
0	--	9.022	0.000
1	2S	9.022	6.080
2	2D	10.003	21.771
3	2S	10.003	53.937
4	2D	10.003	98.851
5	2S	11.768	157.004

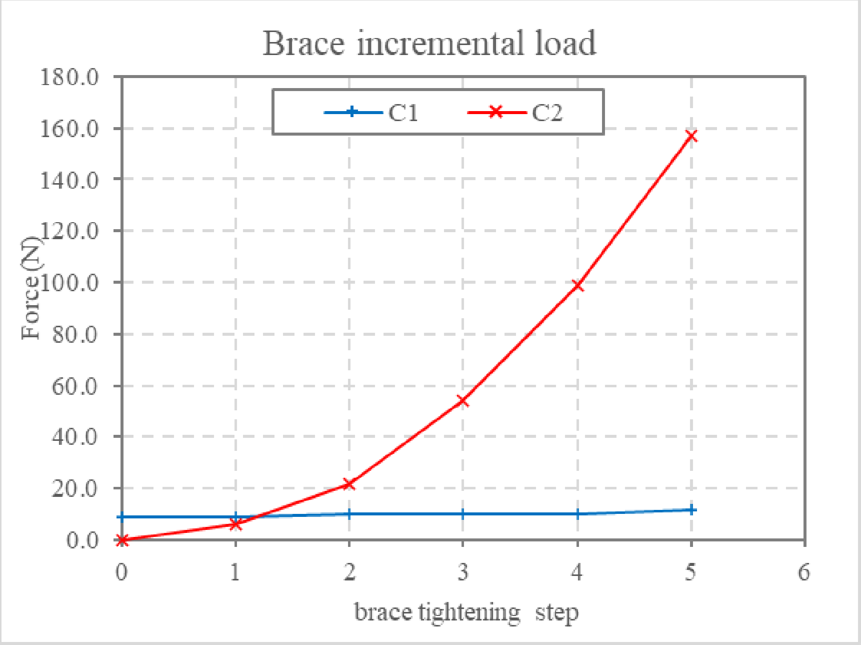

© MaCh3D s.r.l. Proprietary & Confidential

Development of the Numerical Torso Model (FEM)

– *Preparation of the CAD model for performing a FEM analysis of the brace mounted on the stylized torso, developed to validate the experimental results and, subsequently, to allow for various additional measurements and assessments.*

© MaCh3D s.r.l. Proprietary & Confidential

Introduction and aim of the activity

A complementary Finite Element (FE) model was developed to simulate the torso's behaviour observed in the tests:

- The core aim of this research is to correlate and validate the numerical outcomes against the experimental data.

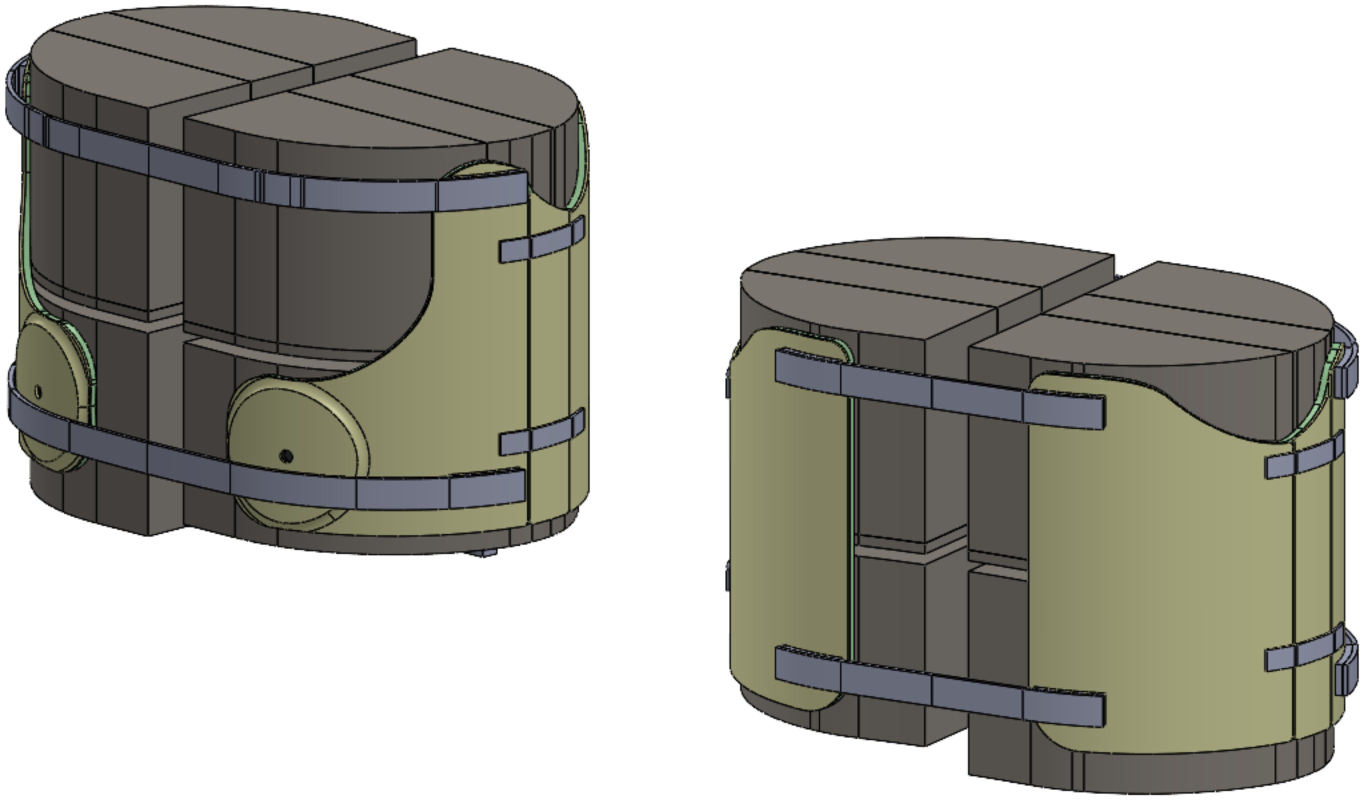

© MaCh3D s.r.l. Proprietary & Confidential

CAD Model - Composite Plates Detail

The model of the composite plates was created from a scan of the physical torso:

- A uniform thickness of 1.5 mm was assigned, matching the theoretical design specification.
- the pressure pads, are modeled to fit into the housing on the composite pads.

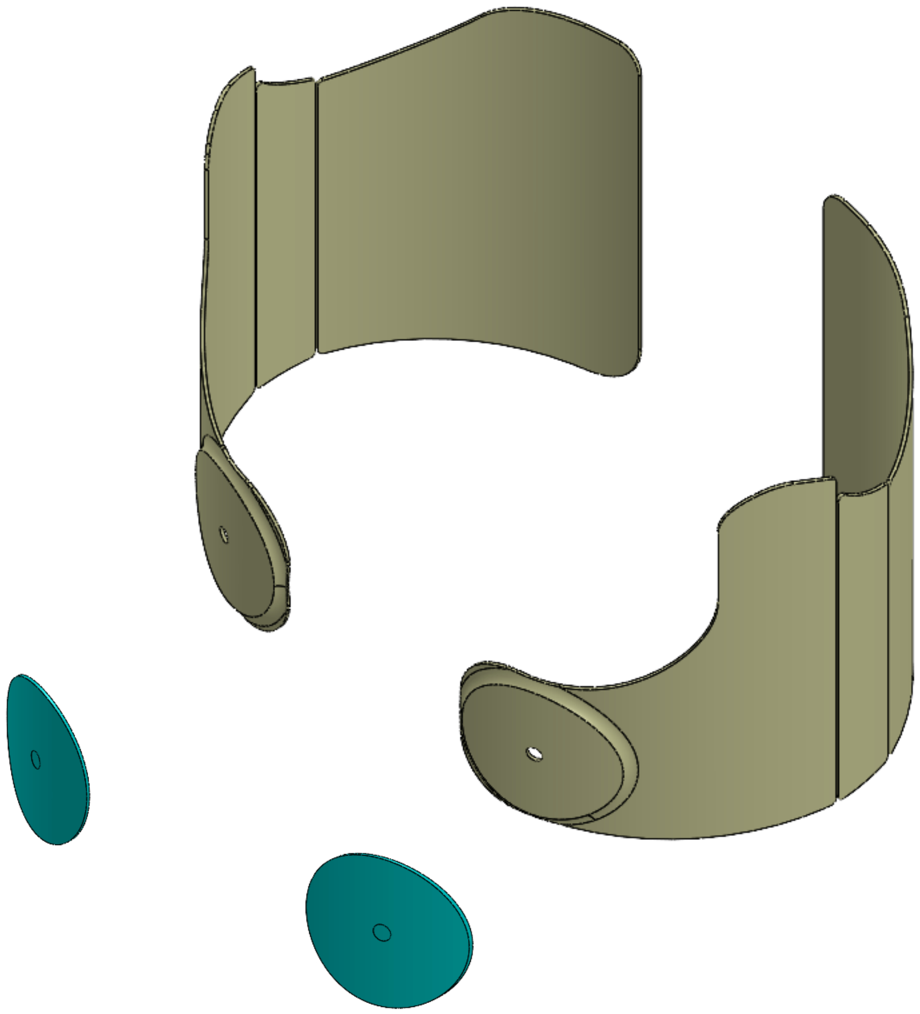

© MaCh3D s.r.l. Proprietary & Confidential

CAD Model - Neoprene detail

The neoprene layer was generated from the plate geometry.:

- A thickness of 3.5 mm was assigned, representing the gap between the plates and the mannequin.

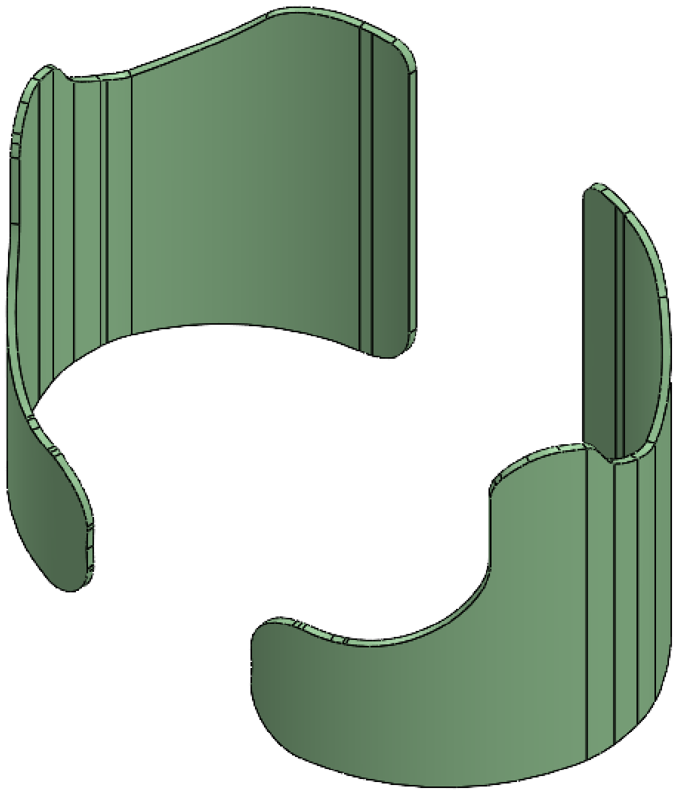

© MaCh3D s.r.l. Proprietary & Confidential

CAD Model - Mannequin Detail

The mannequin model was derived from the assembly of plates and neoprene:

- Its geometry was simplified to facilitate the FEM analysis
- Specifically, unnecessary fillets and features were removed, and it was split into blocks to simplify the meshing process.

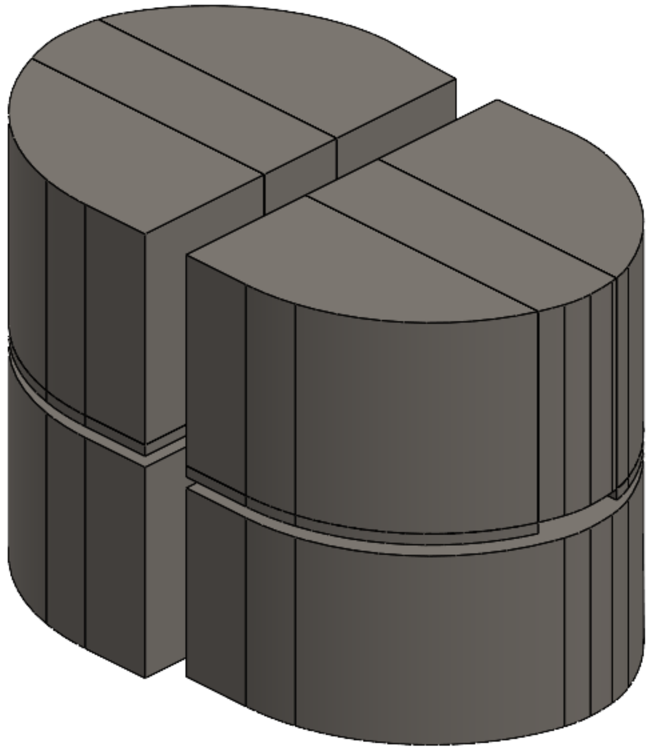

© MaCh3D s.r.l. Proprietary & Confidential

CAD Model - Guide Rails Detail

To simplify the FEM analysis, the guide rail / carriage systems were modeled as simple block geometries:

- A sliding contact was defined between the parts to simulate the real behavior of the rails.

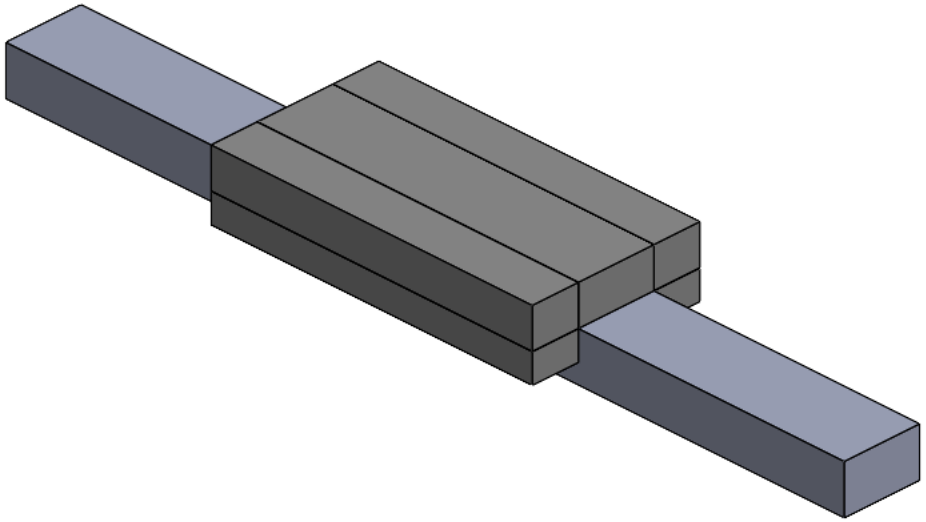

© MaCh3D s.r.l. Proprietary & Confidential

CAD Model - Rack Detail

The clamping racks were modeled following the geometry of the torso and mannequin:

- The hooks, modeled as part of the racks, are used to secure the racks to the torso, simulating their real function.
- In the FEM model, the hooks are bonded to the torso.
- The hook placement was done according to measurements taken on the physical torso

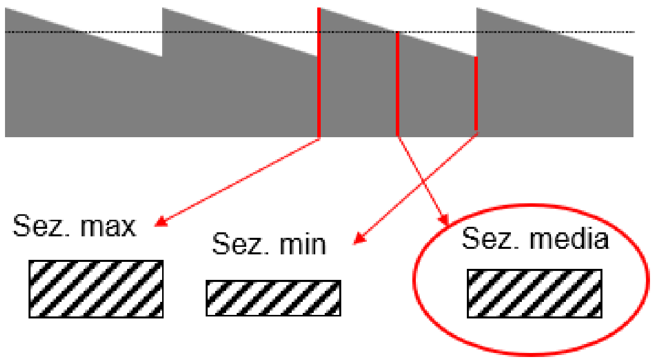

Equivalent Section of the FEM Model

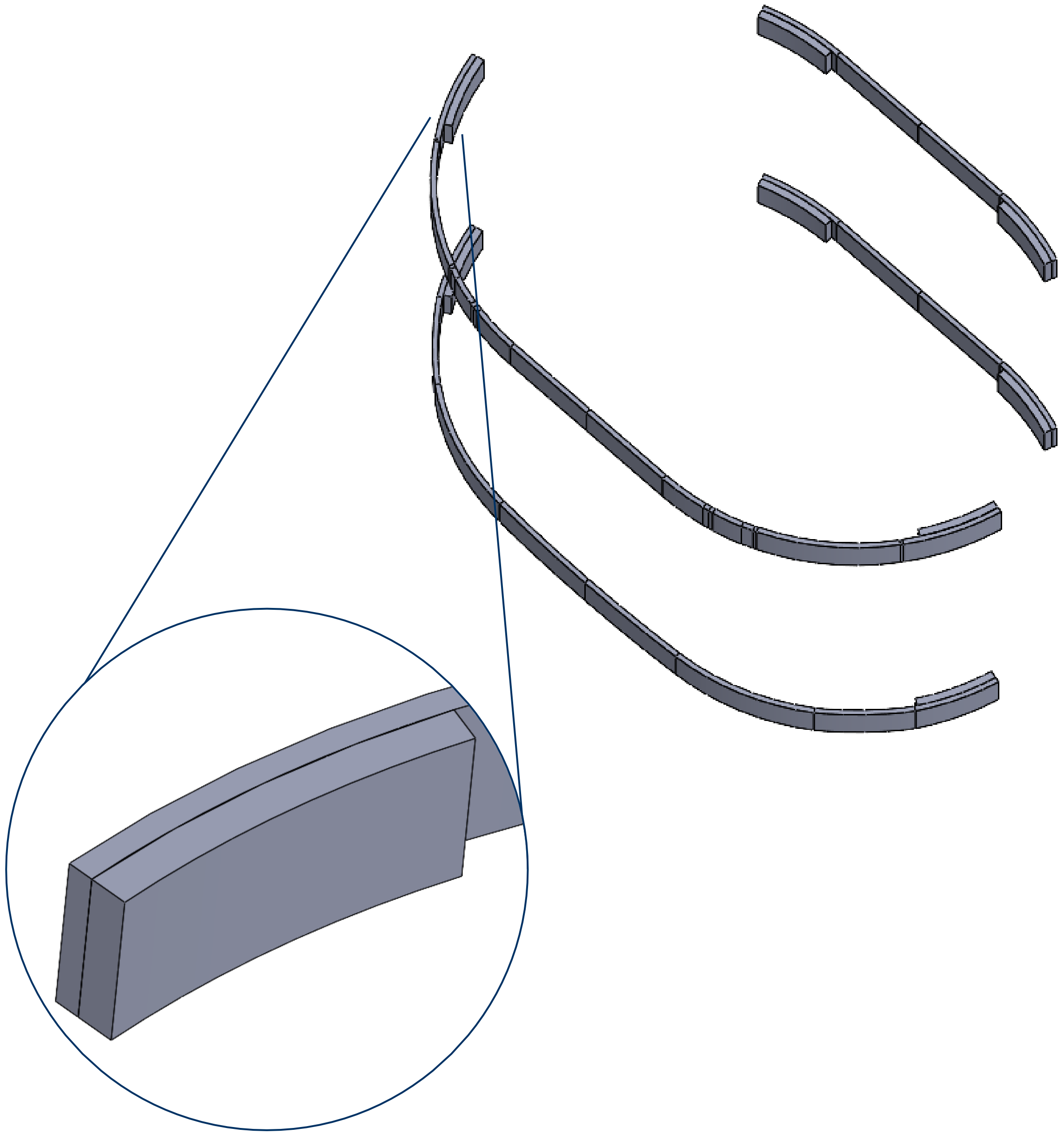

Rack Hook Detail

© MaCh3D s.r.l. Proprietary & Confidential

CAD Model - Lacing Cords Detail

The lacing cords were modeled based on the position of the tightening screws measured on the physical torso.

- Multiple versions were created:
  - One perfectly in contact with the torso from the start.
  - Multiple versions initially loose, to evaluate how to best simulate their effect.

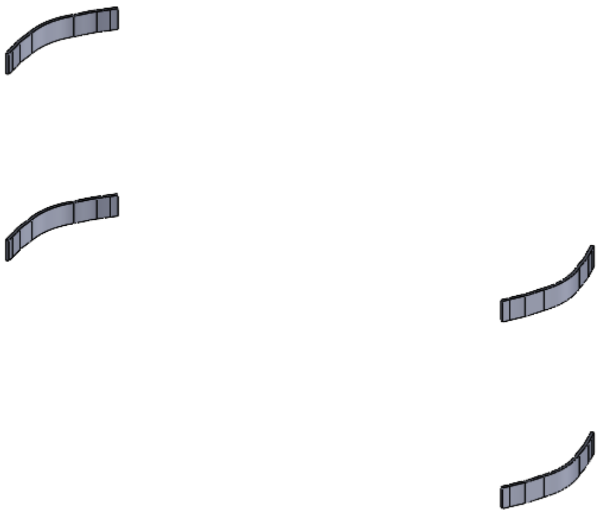

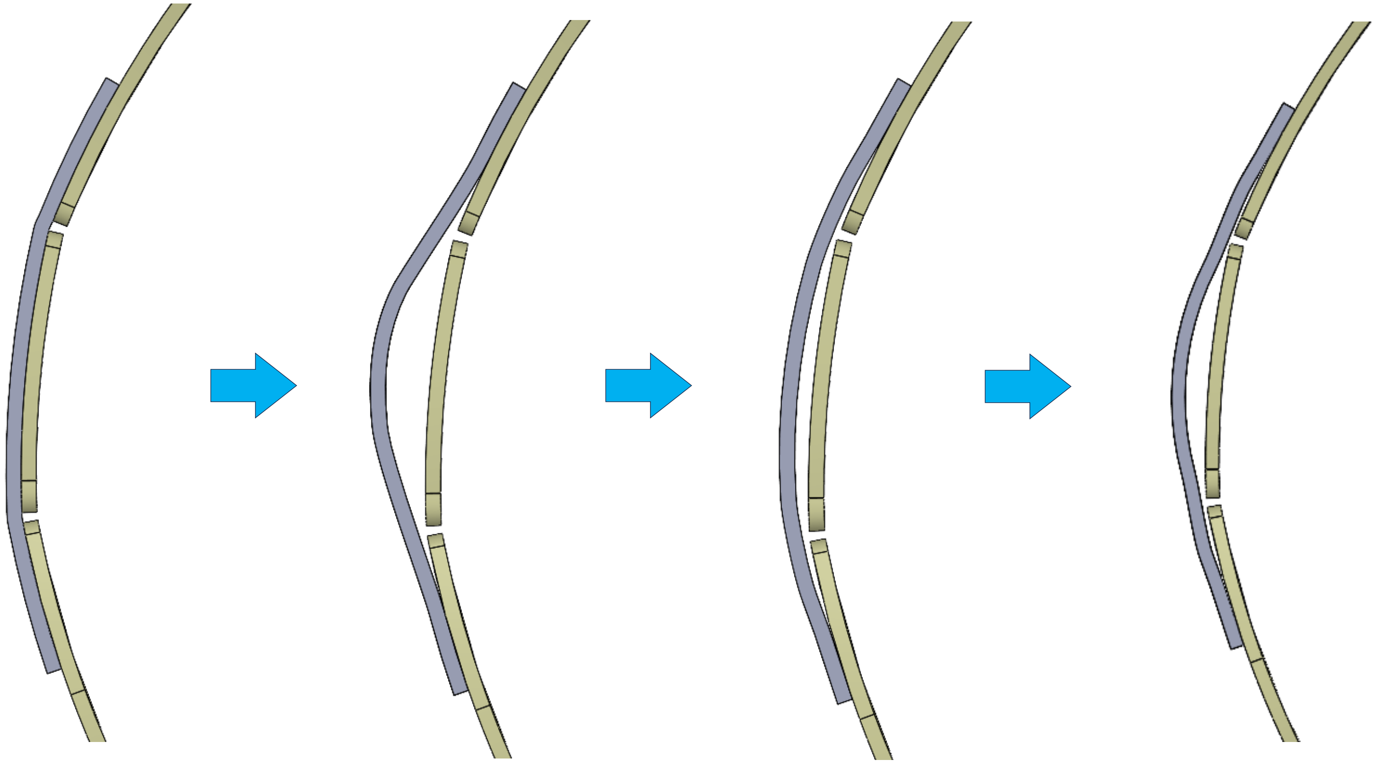

FEM Model - Load Cells

© MaCh3D s.r.l. Proprietary & Confidential

The load cells were modeled using 1D elements:

- For the button load cells, 1D "rod" elements were used to simulate their compressive action. This setup allows for the extraction of the force measured by the cells during the numerical test.
- For the shear load cell, a 1D "beam" element was used, connected to other beam elements to simulate its linkages to the mannequin.
- The properties of all load cells were defined according to the stiffness provided by the manufacturer.

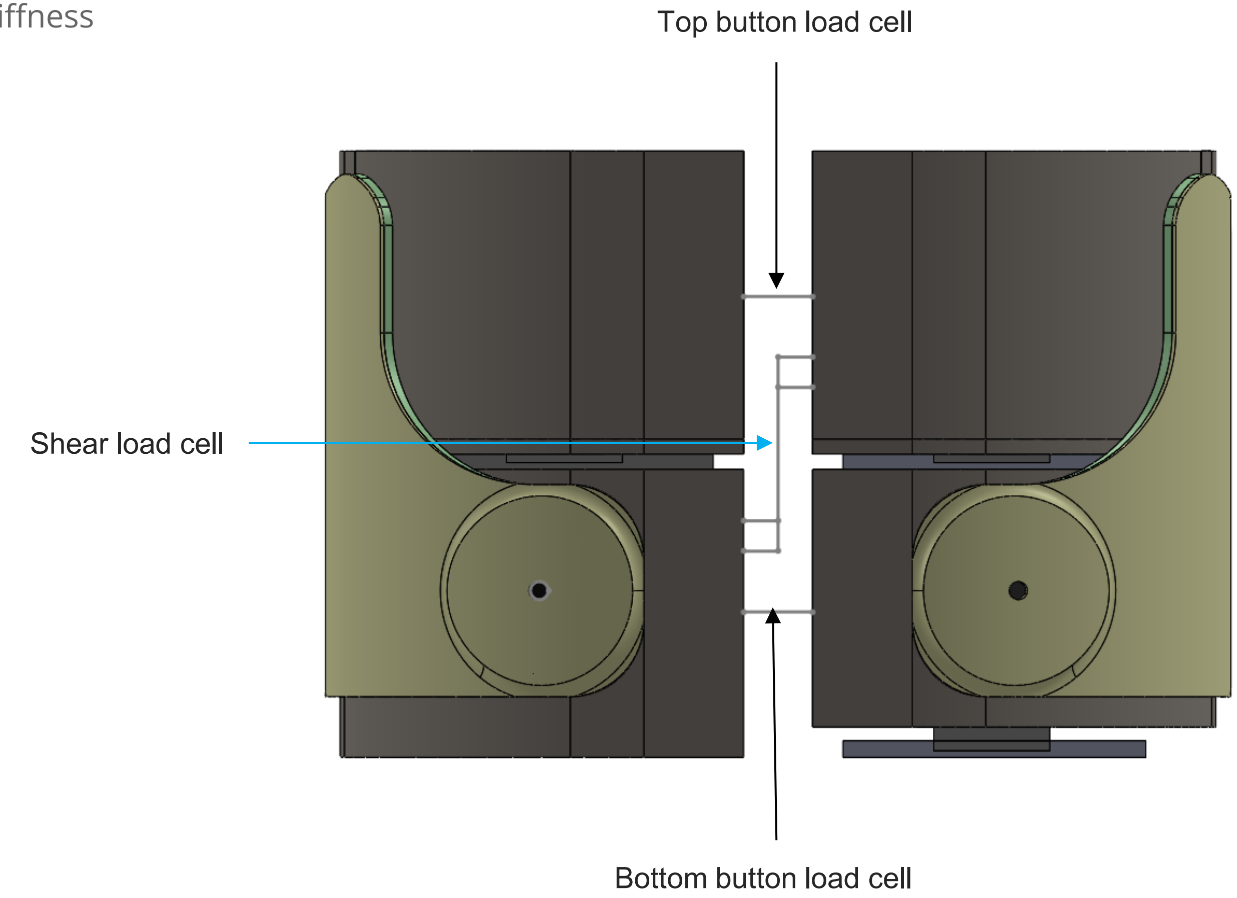

© MaCh3D s.r.l. Proprietary & Confidential

FEM Model - Loads and Constraints

This slide presents the constraints and loads applied to the FEM model.

- The model was constrained at the base with fixed supports on the lower guide and the left mannequin.
- The tightening action of the racks, caused by the "clicks" of the mechanism, was simulated by imposing a contraction on the racks.
- This was achieved by assigning a thermal expansion coefficient to the racks.
- The tightening is activated by applying a calculated temperature difference (details in the next slide).
- In line with the experimental setup, "clicks" were applied only to the front racks.

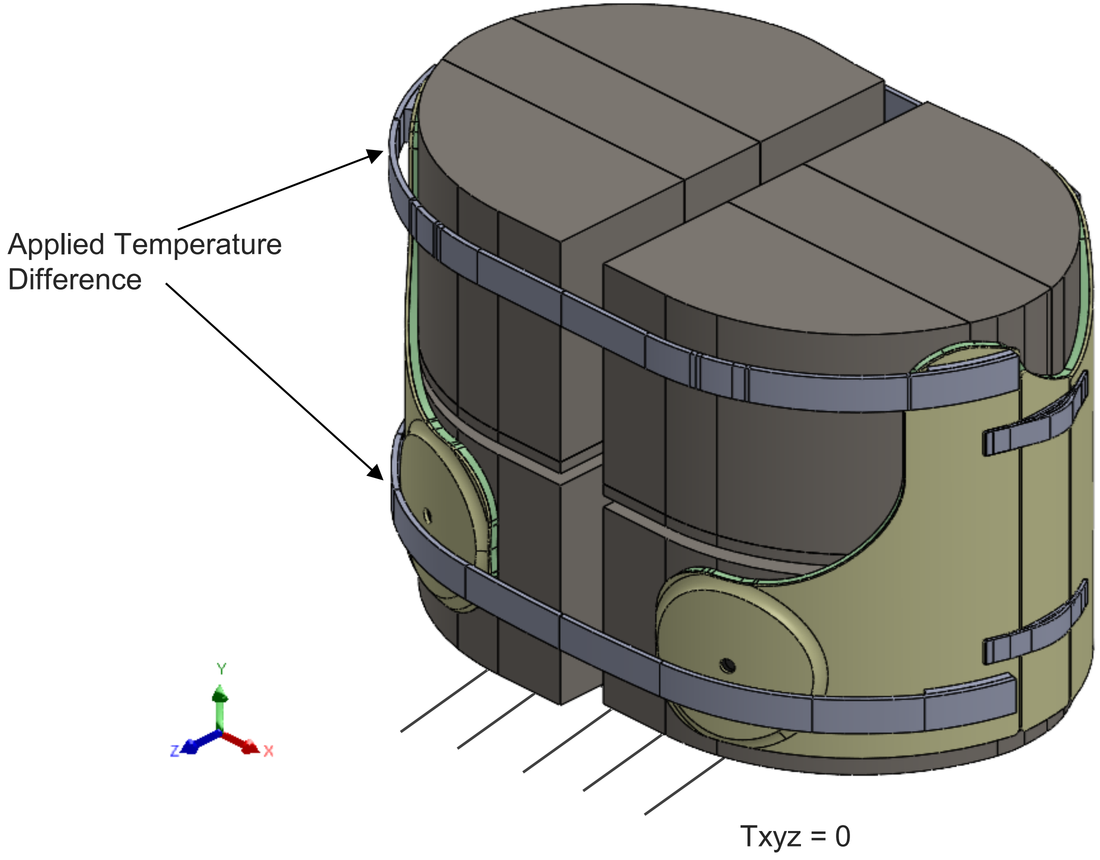

© MaCh3D s.r.l. Proprietary & Confidential

FEM Model - Load Application Detail

The tightening action was simulated thermally using this method:

- A thermal expansion coefficient (α) was assigned to the racks
- The imposed temperature difference (ΔT) was calculated analytically, based on the initial rack length (L_0) and the shortening corresponding to one "click" on the physical torso (ΔL).
- A load history was finally defined to simulate the experimental campaign

Definition of the Thermal Expansion Coefficient:

$$\alpha = \frac{1}{L_0} * \frac{\Delta L}{\Delta T}$$

$\alpha\ [°C^{-1}]$ *Thermal expansion coefficient*

ΔL [mm] *Change in rack length (equal to the displacement per one "click")*

ΔT [°C] *Imposed temperature change in the FEM model*

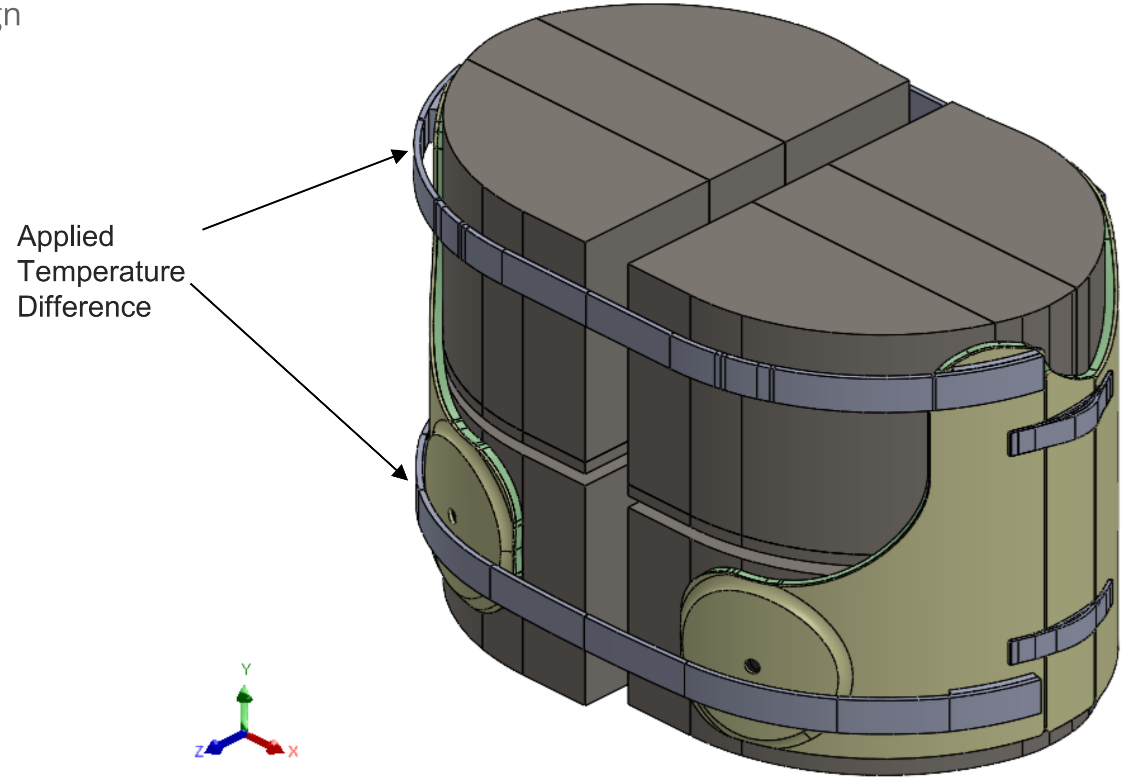

© MaCh3D s.r.l. Proprietary & Confidential

FEM Model – Results extraction

To compare the numerical and experimental results, the axial force is extracted from the button load cells, which are simulated using 1D rod elements:

- This is done for both the upper and lower cells
- The detected forces are then plotted against the number of clicks, in accordance with the experimental procedure.
- These numerical curves are finally compared with the data from the experimental load cells.

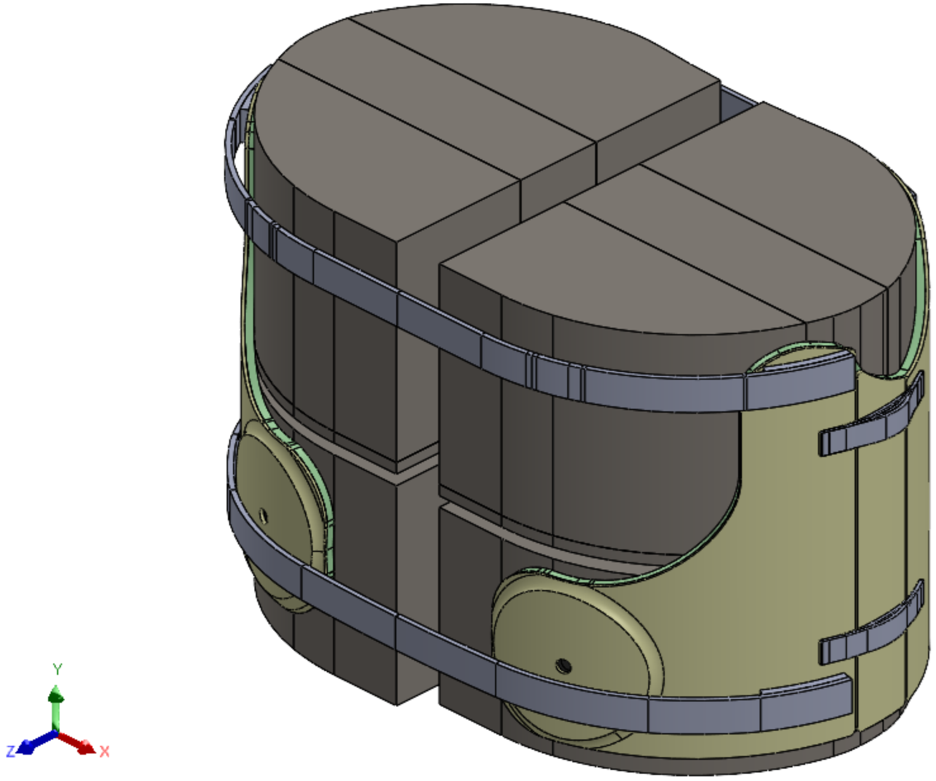

© MaCh3D s.r.l. Proprietary & Confidential

Complementary Numerical Analysis

© MaCh3D s.r.l. Proprietary & Confidential

FEM Model – Calibration

To calibrate the numerical model against the experimental data, several iterative runs were performed, involving:

- Adjustment of material properties.
- Modification of the lacing cord geometry (see Slide 19).

The calibration test involves applying 2.5 clicks to the lower front rack (with the upper front rack absent), in accordance with the experimental setup. The rear racks are present but unloaded.

The curve obtained from the FEM analysis shows the compressive force measured by the lower button load cell as a function of the rack's shortening.

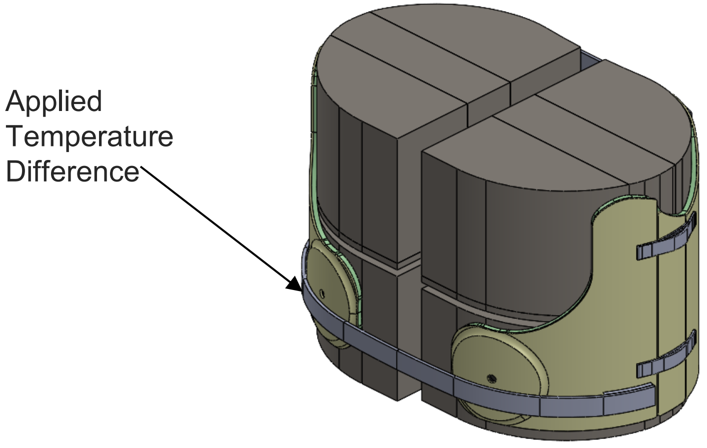

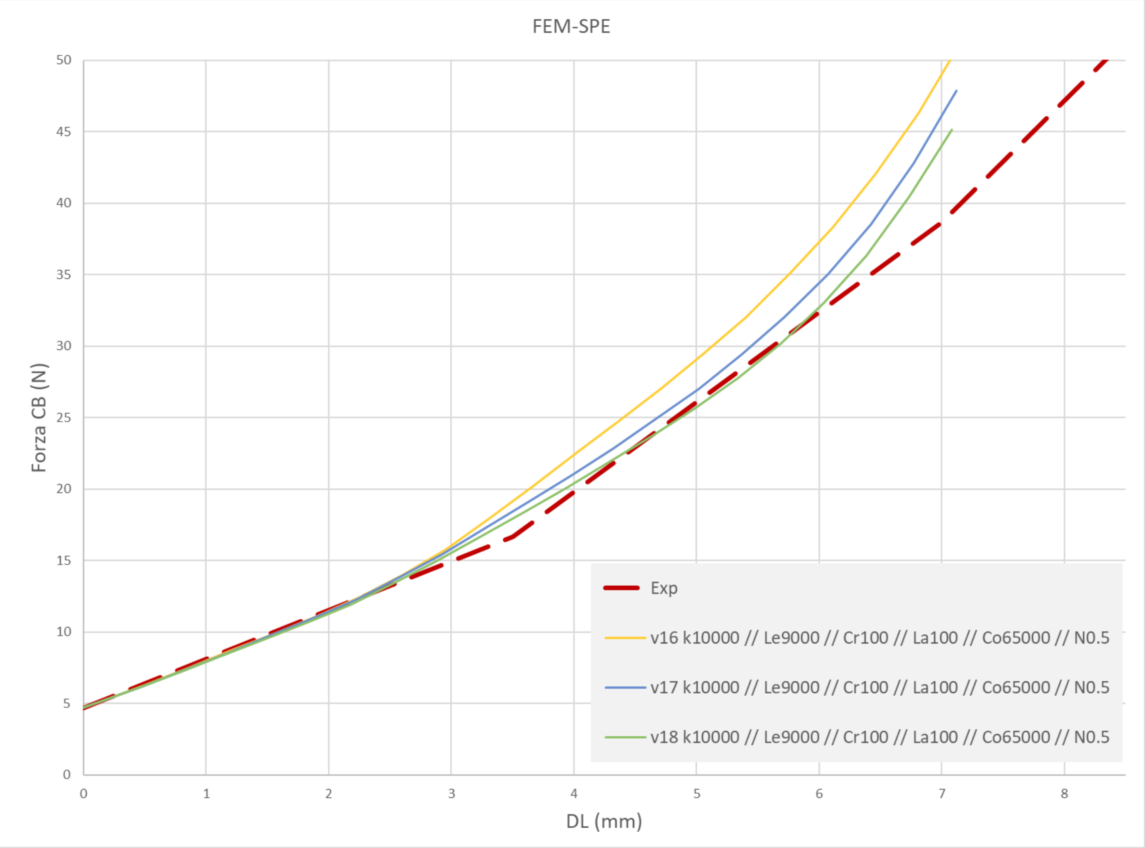

© MaCh3D s.r.l. Proprietary & Confidential

FEM Model, Calibration conclusion

Comparison of Numerical and Experimental Results

This graph shows the numerical curve that best approximates the experimental behavior.

The numerical curve matches the experimental data reasonably well, except for the final section.

In this final part, the lacing cords become fully engaged, leading to a sharp increase in the system's overall stiffness.

K bottom load cell	10000	N/mm
E wood	9000	MPa
E crack	100	MPa
E cords	70	MPa
E composite plates	65000	MPa
E neoprene	0.5	MPa

Material Properties (Young's Moduli / Stiffness)

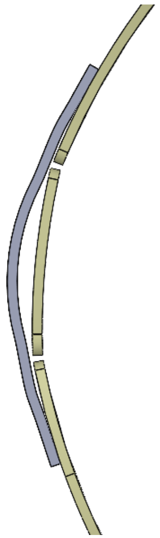

Geometry of the lacing cords

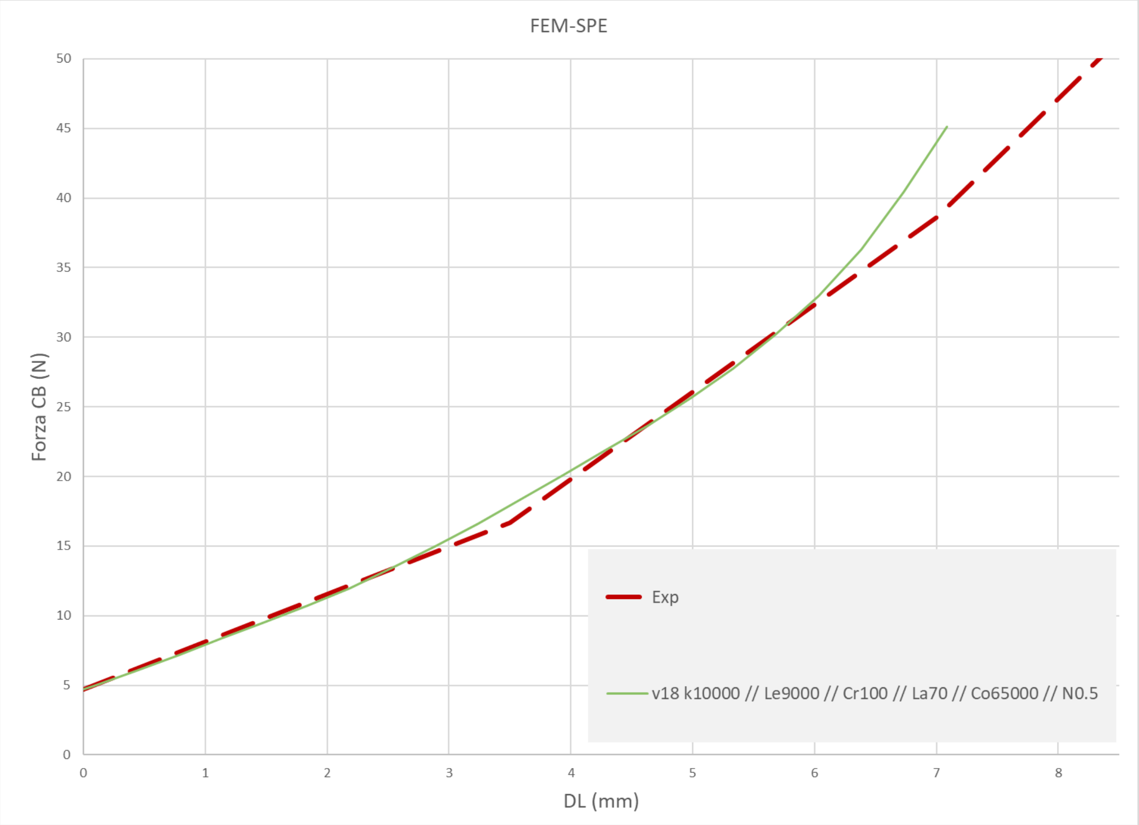

© MaCh3D s.r.l. Proprietary & Confidential

FEM Model – Load Story

For the final FEM characterization test, the load history was created to simulate the sequence of "clicks" applied during the experimental tests.

- The initial temperature of the system is 0°C.
- The preload is the same for both cells
- Before following the main "click" load sequence, a temperature difference corresponding to the predetermined preload was applied
- The "clicks" are divided between the upper and lower racks (no distinction is made between left and right).
- For each "click," the temperature of the racks is increased to simulate a free shortening of the rack equal to one click's displacement.

The load sequence is defined as follows

	Rack Temperature [°C]	
Step	Upper	Lower
Initial	0.000	0.000
Preload	0.073	0.074
Click01_upper	0.184	0.074
Click02_lower	0.184	0.186
Click03_lower	0.184	0.298
Click04_upper	0.294	0.298
Click05_uper	0.405	0.298
Click06_lower	0.405	0.410
Click07_lower	0.405	0.522
Click08_upper	0.516	0.522
Click09_lower	0.516	0.635

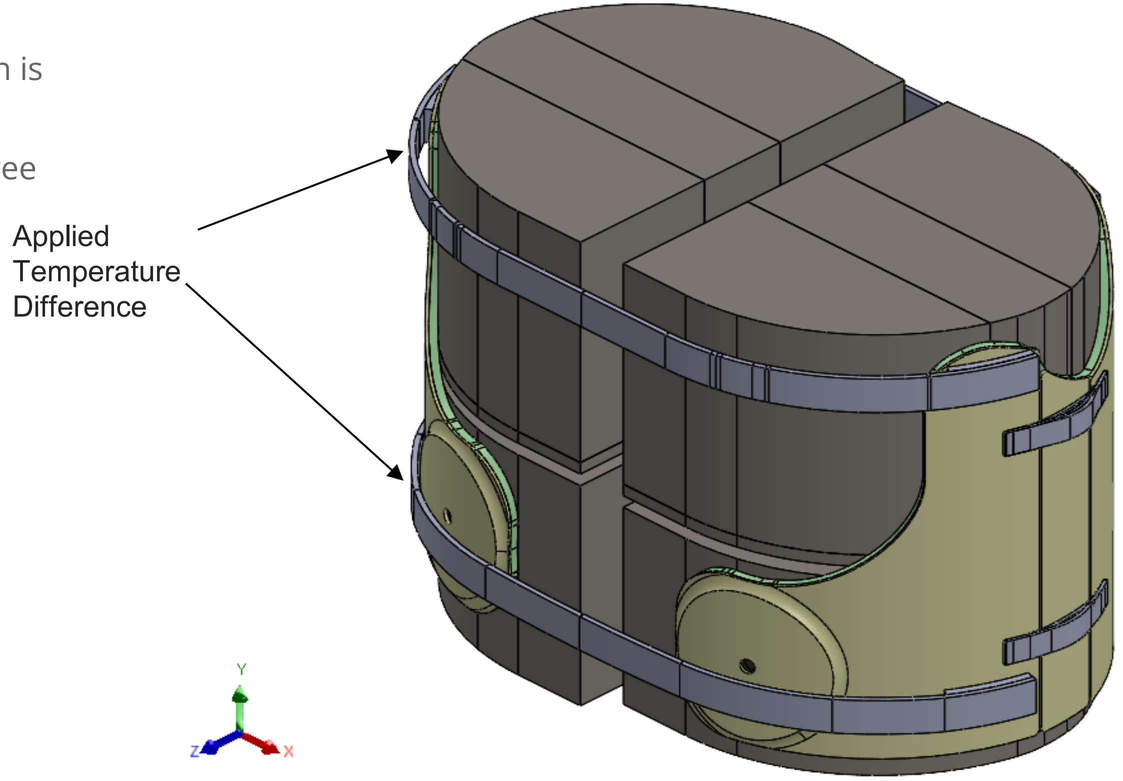

FEM Model – Results

The results of the final characterization test are reported.

- The load history was applied as defined in the previous slides.
- The results show that the torso-manikin system is much stiffer compared to the experimental tests.
- In the FEM model, it is observed that the action on the racks significantly influences both cells, unlike the experimental results.
- The FEM curves show a parabolic behavior.

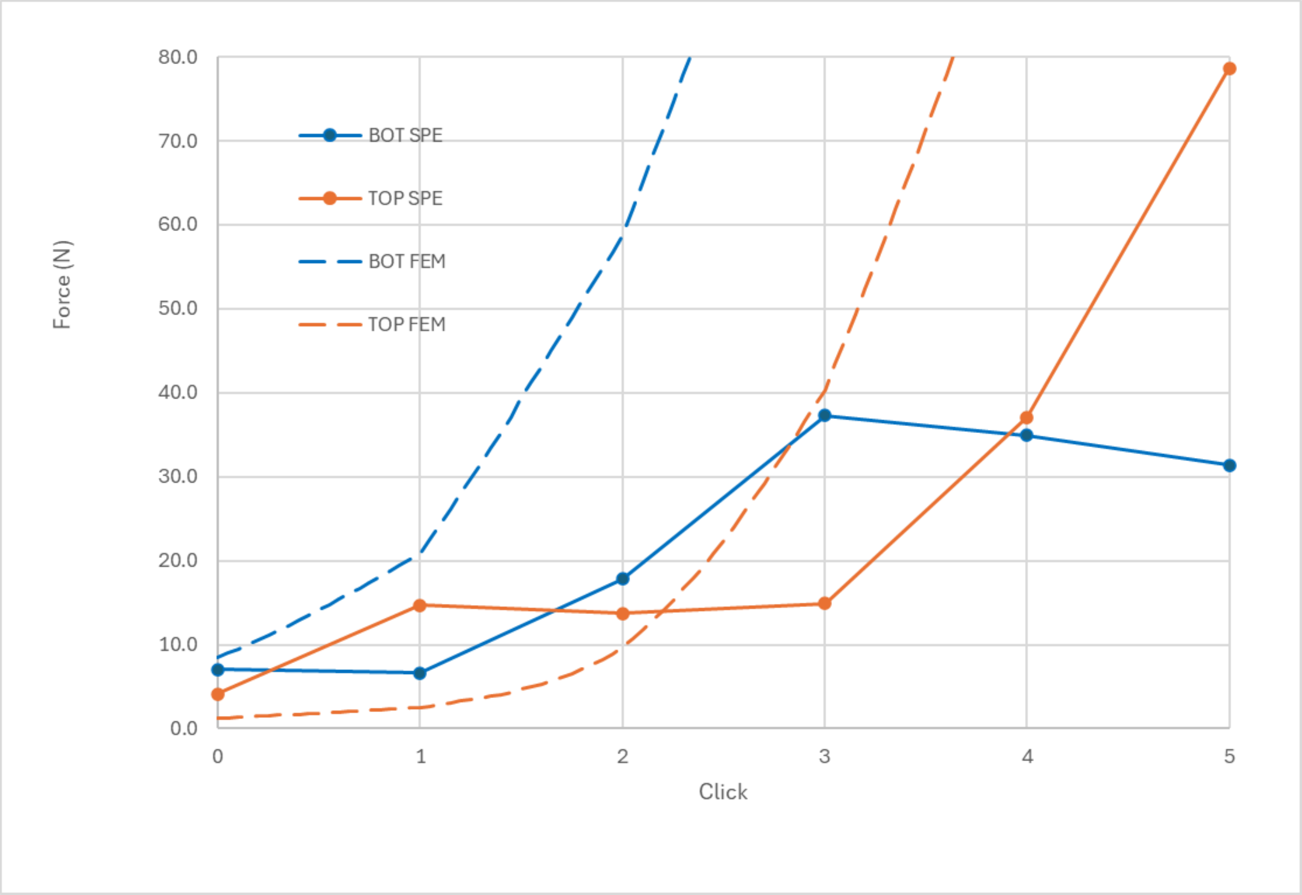

© MaCh3D s.r.l. Proprietary & Confidential
